# Genome-Wide Identification and Expression Profile of Dof Transcription Factor Gene Family in Pepper (*Capsicum annuum* L.)

**DOI:** 10.3389/fpls.2016.00574

**Published:** 2016-04-29

**Authors:** Zhiming Wu, Jiaowen Cheng, Junjie Cui, Xiaowan Xu, Guansheng Liang, Xirong Luo, Xiaocui Chen, Xiangqun Tang, Kailin Hu, Cheng Qin

**Affiliations:** ^1^College of Horticulture and Landscape Architecture, Zhongkai University of Agriculture and EngineeringGuangzhou, China; ^2^College of Horticulture, South China Agricultural UniversityGuangzhou, China; ^3^Vegetable Research Institute, Guangdong Academy of Agricultural SciencesGuangzhou, China; ^4^Pepper Institute, Zunyi Academy of Agricultural SciencesZunyi, China; ^5^Guizhou Provincial College-based Key Lab for Tumor Prevention and Treatment with Distinctive Medicines, Zunyi Medical UniversityZunyi, China

**Keywords:** pepper, DNA-binding one zinc finger, phylogenetic analysis, expression analysis, heat stress, salinity stress

## Abstract

Dof (DNA-binding One Zinc Finger) transcription factor family is unique to plants and has diverse roles associated with plant-specific phenomena, such as light, phytohormone and defense responses as well as seed development and germination. Although, genome-wide analysis of this family has been performed in many species, information regarding Dof genes in the pepper, *Capsicum annuum* L., is extremely limited. In this study, exhaustive searches of pepper genome revealed 33 potential *CaDofs* that were phylogenetically clustered into four subgroups. Twenty-nine of the 33 *Dof* genes could be mapped on 11 chromosomes, except for chromosome 7. The intron/exon organizations and conserved motif compositions of these genes were also analyzed. Additionally, phylogenetic analysis and classification of the Dof transcription factor family in eight plant species revealed that *S. lycopersicum* and *C. annuum* as well as *O. sativa* and *S. bicolor* Dof proteins may have evolved conservatively. Moreover, comprehensive expression analysis of *CaDofs* using a RNA-seq atlas and quantitative real-time polymerase chain reaction (qRT-PCR) revealed that these genes exhibit a variety of expression patterns. Most of the *CaDofs* were expressed in at least one of the tissues tested, whereas several genes were identified as being highly responsive to heat and salt stresses. Overall, this study describes the first genome-wide analysis of the pepper Dof family, whose genes exhibited different expression patterns in all primary fruit developmental stages and tissue types, as in response to abiotic stress. In particular, some *Dof* genes might be used as biomarkers for heat and salt stress. The results could expand our understanding of the roles of *Dof* genes in pepper.

## Introduction

The DNA-binding one zinc finger (Dof) protein is a representative of the plant-specific members of transcription factors (TFs). All Dof transcription factors share a common DNA-binding domain (C_2_C_2_-Dof) that is highly conserved in the N-terminal region composed of approximately 52 amino acid residues. It was predicted that the C_2_C_2_-Dof motif may form a single zinc finger that is essential for binding a conversed target DNA sequence with a 5′-(T/A)AAAG-3′ core (Yanagisawa and Schmidt, 1999). The C-terminal region of Dof proteins is highly variable. This unstable C-terminal domain could act as either a transcriptional activator or repressor in the control of the expression of many structural genes, and execute different regulatory functions (Guo et al., 2009; Cominelli et al., 2011; Corrales et al., 2014).

The Dof TFs have been reported to be involved in a wide spectrum of biological processes, such as light-responsiveness (Yanagisawa and Sheen, 1998; Park et al., 2003; Ward et al., 2005), seed development, maturation, and germination (Diaz et al., 2002, 2005; Gualberti et al., 2002; Papi et al., 2002; Isabel-LaMoneda et al., 2003; Dong et al., 2007; Gabriele et al., 2010; Gaur et al., 2011; Santopolo et al., 2015). Meanwhile there were evidenced that some Dof TFs participated in plant hormone and stress responses as well (Skirycz et al., 2006; Corrales et al., 2014; Xu et al., 2014; Jiang et al., 2015; Song et al., 2016). In *Arabidopsis*, more than 10 Dof proteins, including the OBF BINDING PROTEIN (OBP1, OBP2, and OBP3), Dof Affecting Germination (DAG1 and DAG2), and Cycling Dof Factors (CDF1-5) (Imaizumi et al., 2005; Skirycz et al., 2008; Fornara et al., 2009) have been functionally characterized. Among them, OBP1 might be involved in glutathione S-transferases 6 (GST6) expression and respond to plant hormones and stress (auxin, salicylic acid, and H_2_O_2_) signals (Pan et al., 2014). OBP1 could also control cell division by regulating the expression of several cell cycle-associated genes (Skirycz et al., 2008). OBP2 (*AtDof1.1*) is involved in the regulation of glucosinolate biosynthesis in Arabidopsis (Skirycz et al., 2006). OBP3 plays important roles in plant growth and development (Kang and Singh, 2000) and is characterized as a novel component of light signaling (Ward et al., 2005). The DAG1 and DAG2 proteins are also involved in light-dependent seed germination in *Arabidopsis* (Gualberti et al., 2002; Papi et al., 2002; Gabriele et al., 2010). CDFs play an important role in photoperiodic flowering in *Arabidopsis* through by binding directly to the C2C2-Dof sites in the CONSTANS (*CO*) promoter to repress *CO* transcription (Imaizumi et al., 2005). Combining loss-of-function mutations in four of these genes (*CDF1, 2, 3*, and *5*) causes photoperiod-insensitive early flowering by increasing the *CO* mRNA level (Fornara et al., 2009; Corrales et al., 2014).

The Dof gene family has also been comprehensively identified in several plants based on the completion of an increasing number of genome sequencing projects. Thirty-six and 30 *Dof* genes have been found in the model plant *Arabidopsis* and rice, respectively (Yanagisawa, 2002; Lijavetzky et al., 2003), 27 in *Brachypodium distachyon* (Hernando-Amado et al., 2012), 31 in wheat (Shaw et al., 2009), 26 in barley (Moreno-Risueno et al., 2007), 28 in sorghum (Kushwaha et al., 2011), 78 in soybeans (Guo and Qiu, 2013), 34 in tomatoes (Cai et al., 2013), 35 in potatoes (Venkatesh and Park, 2015), 76 in Chinese cabbage (Ma et al., 2015), 38 in pigeonpeas (Malviya et al., 2015), and 36 in cucumber (Wen et al., 2016). According to the sequence similarity, the Dofs could be organized into four groups or subfamilies (A, B, C, and D), and groups B, C, and D could be further subdivided into subgroups (Lijavetzky et al., 2003; Ma et al., 2015; Feng et al., 2016; Wen et al., 2016).

Pepper (*Capsicum* spp.) is one of the most important and widely cultivated vegetable crops belonging to the family Solanaceae, which also includes potatoes, tomatoes, and tobacco, eggplants, etc. Lately, the pepper genome was sequenced (Kim et al., 2014; Qin et al., 2014). In addition, a large number of RNA sequencing reads derived from several tissues such as root, shoot, leaf, flower, and fruit are also available. These datasets provide a framework for the identification and functional characterization of gene family from a global view for pepper improvement and basic research. This study aimed to identify all potential *Dof* genes encoded in the pepper genome. Further, some routine bioinformatics analyses were performed including gene structures, chromosomal distribution and phylogenetic analysis. Finally, functional prediction was performed based on the gene expression analysis in different organs and developmental stages, and in response to heat and salinity stresses. The results gained herein will provide an important foundation for future studies on gene cloning and functional characterization of Dofs in pepper.

## Materials and methods

### Plant materials and stress treatments

Seeds of the pepper cultivar “Zunla-1” (*Capsicum annuum* L.) were provided by the Pepper Institute, the Zunyi Academy of Agricultural Sciences in China. The seeds were first sterilized and germinated in an incubator (28°C), as previously described (Qin et al., 2014). The germinated seeds were then sown in pots and grown under a 16 h day/8 h night cycle (28°C/21°C day/night temperature cycle) until the seedlings developed six leaves. Uniformly developed plants were then exposed to heat (38°C) and NaCl (300 mM) treatments for 3, 6, and 12 h. For all treatments, leaves were harvested from the same position of the seedlings with three biological replicates. For each replicate, leaves from eight plants were put together and rapidly frozen in liquid nitrogen and stored at −80°C until RNA extraction.

### Identification of *Dof* genes in pepper

The conserved Dof domain (PF02701) based on a Hidden Markov Model (HMM) was firstly downloaded from the Pfam database (Pfam 27.0, http://Pfam.sanger.ac.uk/). Then the HMM profile of the Dof domain was used to do BLASTP search in two pepper genome databases (http://peppersequence.genomics.cn/page/species/index.jsp, *release 2.0* cv. Zunla-1; http://peppergenome.snu.ac.kr/, cv. CM334) (Kim et al., 2014; Qin et al., 2014) with an expected value (*e*-value) cut-off of 0.01. All protein sequences obtained were confirmed for the presence of an intact C_2_C_2_-Dof domain by ScanProsite (http://www.expasy.ch/tools/scanprosite/) and SUPERFAMILY 1.75 (http://supfam.org/SUPERFAMILY/hmm.html). In order to obtain the integrated catalog of *Dof* genes in pepper, the output results from two databases were combined and filtered the redundant sequences. The isoelectric points and protein molecular weights of all non-redundant sequences were obtained with the help of the proteomics and sequence analysis tools on the ExPASy proteomics server (http://expasy.org/).

### Chromosomal location and gene structure analysis

The chromosomal location information of each pepper *Dof* was obtained via BLASTP search against the pepper genome database which has been built by our group (Qin et al., 2014) (cv. Zunla-1) with default parameters. The exon and intron structures of individual *Dof* gene were illustrated using the Gene Structure Display Server (GSDS 2.0, http://gsds.cbi.pku.edu.cn/index.php) by aligning the cDNA sequences with the corresponding genomic DNA sequences.

### Conserved motif analysis

Functional motifs or domains of Dof protein sequences were analyzed using PROSITE (http://prosite.expasy.org/) and the Conserved Domain database (http://www.ncbi.nlm.nih.gov/cdd/). MEME (http://meme.nbcr.net/meme/cgi-bin/meme.cgi) (Version 4.9.1; Bailey et al., 2009) was used to identify motifs in candidate sequences. MEME was run online using the following parameters: Distribution of motif occurrences: Any number of repetitions, Number of different motifs: 25, Minimum/Maximum number of sites: 5/100, Minimum/Maximum motif width: 6/100.

### Phylogenetic analyses

Multiple alignments of the full-length protein sequences were performed using Clustal Omega (http://www.ebi.ac.uk/Tools/msa/clustalo/). The phylogenetic tree was constructed and drawn by using MEGA 6.06 program (http://www.megasoftware.net/) (Tamura et al., 2013) by the neighbor-joining (NJ) method with 1000 bootstrap replicates. Only clades with a test value higher than 50 were selected for the consensus tree.

### RNA isolation and quantitative real-time PCR

Total RNA from leaves was isolated using the Trizol reagent (Invitrogen, Carlsbad, CA, USA), according to the manufacturer's protocol. RNA was treated with RNase-free DNase I (Takara, Dalian, China) to remove any contamination of genomic DNA. First-strand cDNA synthesis was carried out with approximately 1 μg RNA using the PrimeScript reverse transcription kit (Takara, Dalian, China) and random primers, according to the manufacturer's procedure. Primers with melting temperatures of 58–60°C, lengths of 20–27 bp, and product lengths of 100–250 bp were designed using Primer Premier 5.0 software. All primer sequences are listed in Additional File 6.

qRT-PCR was performed on an CFX96 instrument (Bio-Rad, Alfred Nobel Drive Hercules, CA, USA) using SYBR Green qPCR kits (Kapa Biosystems, Boston, Massachusetts, USA), according to the manufacturer's instructions. The constitutive *actin* gene served as the endogenous control is AY572427 described previously (Lee et al., 2009). PCR was done in 20 μL volumes containing a 250 nM concentration of each primer, 40 ng of cDNA and 10 μL of KAPA SYBR® FAST Universal 2X qPCR Master Mix (KAPA SYBR® DNA polymerase is an engineered version of *Taq* DNA polymerase, designed specifically for real-time PCR using SYBR Green I chemistry). The PCR amplification condition included an initial heat-denaturing step at 95°C for 5 min; then 40 cycles at 95°C for 5 s and at 58°C for 50 s. Fluorescence was measured at the end of each cycle. A melting-curve analysis was performed by heating the PCR product from 65 to 90°C. Expression level of each *CaDof* gene was calculated using the 2^−ΔΔCt^ method, as previously described (Livak and Schmittgen, 2001). SPSS 19.0 (SPSS Inc., USA) was used for statistical analysis, and the Dunnett's *t*-test was used to detect significant difference between all stress treatments and their controls.

### Pepper RNA-seq data analysis

For expression profiling analysis of pepper *Dof* genes, we utilized the Illumina RNA-seq data that were previously generated by pepper genome sequencing (Qin et al., 2014). The expression level of each gene was measured by fragments per kilobase of exon model per million reads mapped (FPKM) values (Additional File 4). Heat maps for above-mentioned genes were generated, which have positive RPKM values in at least one or more of the samples. For the developmental stage dataset, RPKM values were log_2_ transformed before generating heat maps. The heat map was generated by BAR Heatmapper Plus (http://bar.utoronto.ca/ntools/cgi-bin/ntools_heatmapper_plus.cgi) online.

## Results

### Pepper Dof transcription factor gene family isolation

**Figure 1:**
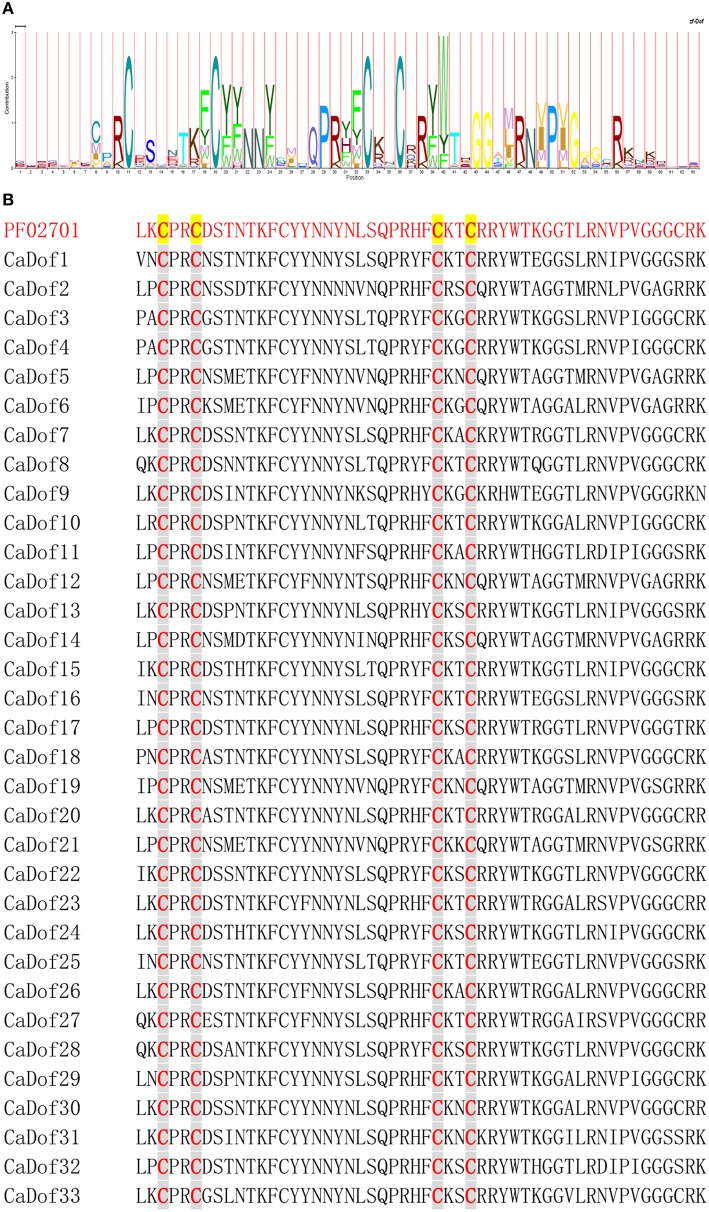
**Multiple sequence alignment of Dof domain in the pepper**. **(A)** Sequence representation LOGO derived from multiple sequence alignment of the Dof motifs. **(B)** Multiple sequence alignment of pepper Dof motifs.

A total of 33 non-redundant *Dof* genes were identified (Additional File 1). All had a typical DNA binding domain of 52 residues spanning a single C2/C2 zinc finger structure (Figure 1). Pepper *Dof* genes were designated as *CaDof1*-*CaDof33* based on the positions of their corresponding genes on chromosomes 1–12 from top to bottom. The full length coding sequences of the *CaDof* genes ranged from 441 bp (*CaDof30*) to 1512 bp (*CaDof14*). The size of deduced Dof proteins varied between 146 and 503 amino acids (aa) with an average of approximately 317 aa. The molecular weight (Mw) varied from 16.2 to 54.3 kDa, and the theoretical pI of these genes ranged from 4.15 (*CaDof18*) to 9.69 (*CaDof30*) (Table 1).

**Table 1:** *****Dof*** transcription factor genes identified and characterized in ***Capsicum annuum*****.

**Gene Name**	**Gene ID**	**Genome position**	**ORF length (bp)**	**Protein length (AA)**	**MW(kDa)**	**PI**	Corresponding gene ID in CM334^*^
*CaDof1*	Capana01g000068	Chr01:1032441–1034119(−)	864	287	32084.52	8.43	CA01g00490
*CaDof2*	Capana01g000644	Chr01:12353976–12356196(+)	1398	465	50496.32	5.99	CA06g18840
*CaDof3*	Capana01g003623	Chr01:231021101–231022072(−)	972	323	36106.63	6.21	CA10g08680
*CaDof4*	Capana01g003624	Chr01:231027264–231028244(+)	981	326	36444.98	6.18	CA00g96300/CA10g08690
*CaDof5*	Capana02g000842	Chr02:96422345–96425020(−)	1413	470	51603.21	4.93	CA02g01120
*CaDof6*	Capana02g001770	Chr02:132754035–132754559(+)	525	174	19716.04	8.48	CA02g14180
*CaDof7*	Capana02g001918	Chr02:135942687–135944043(+)	1131	376	40949.57	8.12	CA02g15190
*CaDof8*	Capana02g001919	Chr02:135984387–135985310(−)	924	307	32958.29	9.68	CA02g15180
*CaDof9*	Capana02g001972	Chr02:136987853–136988686(−)	834	277	31357.21	5.23	CA02g15750
*CaDof10*	Capana02g003147	Chr02:156487162–156488025(+)	864	287	31044.69	8.75	CA02g25910
*CaDof11*	Capana02g003155	Chr02:156578133–156578846(−)	714	237	25034.04	8.70	CA02g25980
*CaDof12*	Capana02g003361	Chr02:159651722–159654531(−)	1338	445	48908.56	7.23	CA02g28150
*CaDof13*	Capana03g000199	Chr03:2790111–2790752(+)	642	213	22823.00	9.02	CA00g78480
*CaDof14*	Capana03g000794	Chr03:11993864–11996778(+)	1512	503	54269.36	5.89	CA03g29970
*CaDof15*	Capana03g001124	Chr03:19167177–19168058(+)	882	293	32650.41	7.94	CA03g26920
*CaDof16*	Capana03g001966	Chr03:39999757–40001103(−)	801	266	28839.61	8.31	CA03g20220
*CaDof17*	Capana04g000477	Chr04:7719982–7720653(+)	672	223	22892.25	6.58	CA04g19530
*CaDof18*	Capana04g001429	Chr04:53467086–53467880(+)	795	264	29729.27	4.15	CA00g84150
*CaDof19*	Capana04g002144	Chr04:176638489–176641282(−)	1308	435	48331.74	6.83	CA11g04250
*CaDof20*	Capana05g000390	Chr05:8716240–8717262(−)	894	297	33559.33	9.19	CA05g18640
*CaDof21*	Capana05g001141	Chr05:71319465–71321812(−)	1410	469	51467.03	6.53	CA05g08190
*CaDof22*	Capana06g000298	Chr06:3884530–3886575(−)	915	304	33822.62	7.38	CA06g23590
*CaDof23*	Capana06g000433	Chr06:6334027–6335212(+)	972	323	34844.60	8.92	CA06g24550
*CaDof24*	Capana06g000951	Chr06:16788242–16789665(−)	924	307	33905.91	6.88	CA06g19670
*CaDof25*	Capana08g001127	Chr08:125490002–125491718(−)	897	298	32368.96	9.07	CA00g64610
*CaDof26*	Capana09g002009	Chr09:222380562–222382228(−)	1230	409	43106.55	9.27	CA00g57880
*CaDof27*	Capana10g001413	Chr10:153100181–153101494(−)	1098	365	39691.05	8.89	CA10g11420
*CaDof28*	Capana11g000662	Chr11:25011115–25012008(−)	894	297	33234.83	7.29	CA00g45170
*CaDof29*	Capana12g002748	Chr12:226954382–226956298(−)	1113	370	40532.36	6.79	CA00g84390
*CaDof30*	Capana00g000317	Chr00:227455491–227456312(−)	441	146	16210.37	9.69	/
*CaDof31*	Capana00g003170	Chr00:530417726–530418343(+)	618	205	22536.74	6.90	CA06g16390
*CaDof32*	Capana00g003618	Chr00:568760736–568761314(−)	579	192	20826.50	8.92	CA02g01550
*CaDof33*	Capana00g004703	Chr00:666278935–666279867(+)	933	310	33595.61	5.56	CA06g18250

* *Gene ID in another pepper genome (CM334): http://peppergenome.snu.ac.kr/, http://bioinfo.bti.cornell.edu/cgi-bin/itak/db_family_gene_list.cgi?acc=C2C2-Dof&plant=Red%20Pepper*.

### Chromosomal localization and gene structure of *CaDof* genes

**Figure 2:**
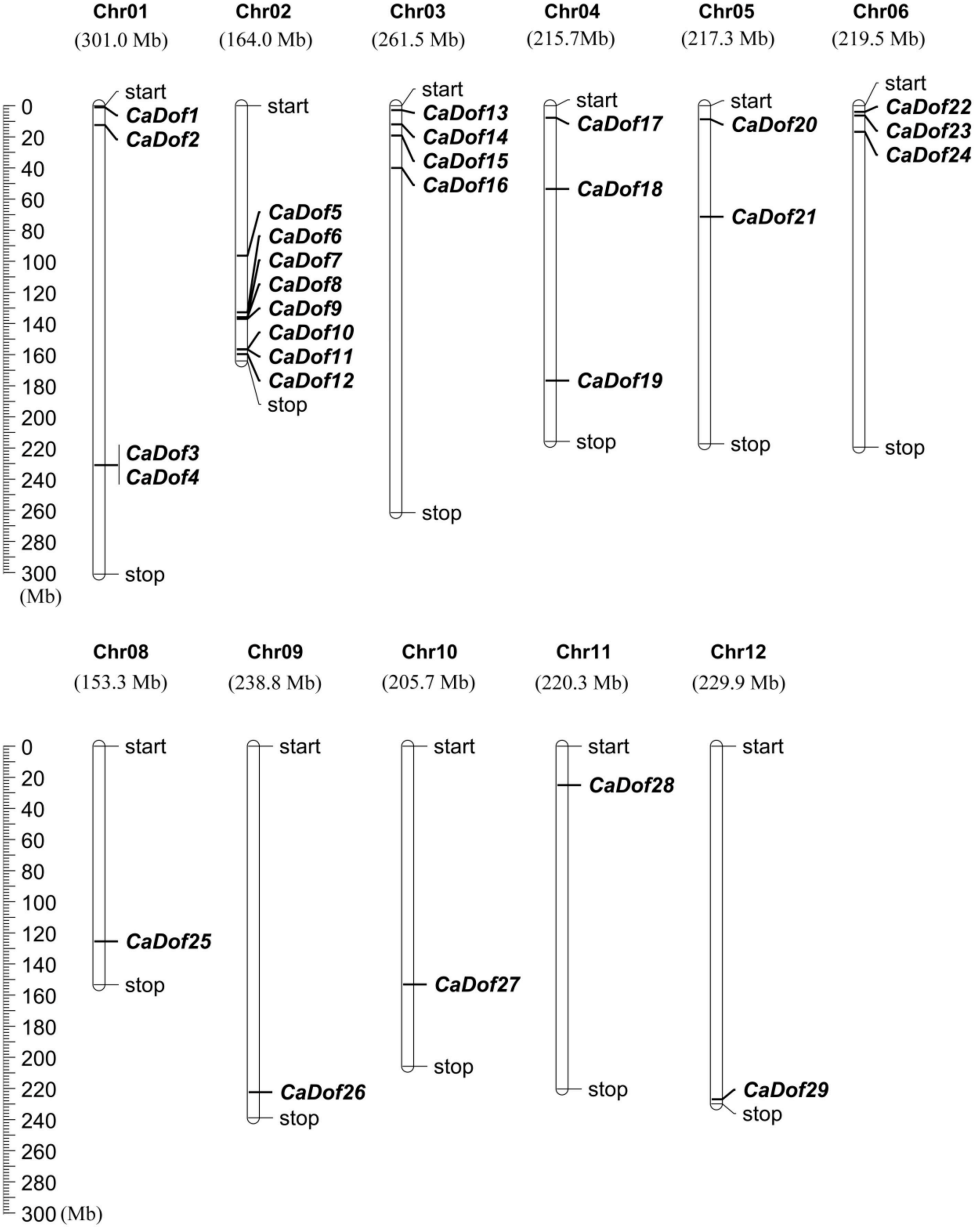
**Distribution of ***CaDof*** genes in pepper chromosomes**. Twenty nine *CaDof* genes were mapped to the 11 linkage groups (Chr01 through Chr12, except Chr07), whereas four *CaDofs* were mapped on a pseudo-chromosome, designated as Chr00.

The physical locations of the *CaDof* genes on pepper chromosomes were identified (Figure 2). The results showed that 29 of the 33 *CaDof* genes could be located on the 11 chromosomes, except chromosome 7, with an obviously non-uniform distribution. However, four members (*CaDof30*-*CaDof33*) could not be anchored on any of the pepper chromosomes. We arranged them on a pseudo-chromosome, designated as Chr00, which was concatenated by the unplaced 3134 scaffolds (705 Mb in total) (Qin et al., 2014). Chromosome 2 contained the largest number of *CaDof* genes (8 members). In contrast, only one *CaDof* gene was found on each of chromosomes 8, 9, 10, 11, and 12.

**Figure 3:**
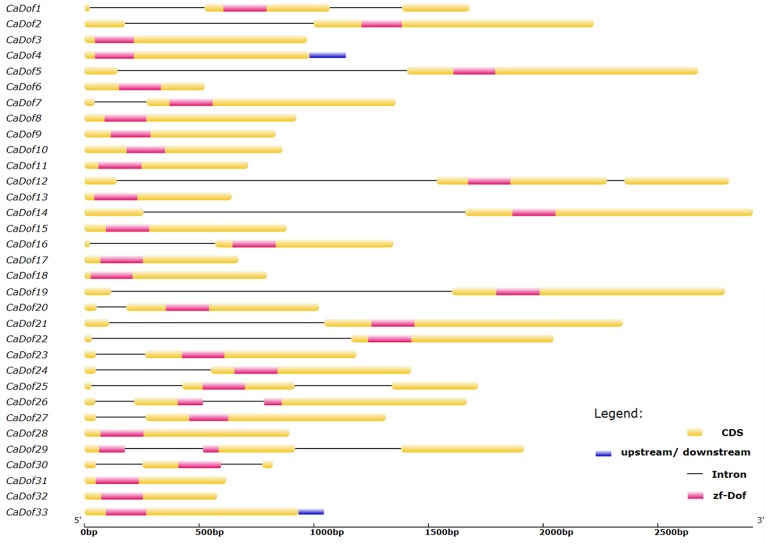
**Exon-intron structures of 33 ***CaDof*** genes**.

In order to gain further insight into their evolutionary imprints, the exon-intron structure of each member of the *CaDof* family was analyzed. The number of introns of the *CaDof* genes ranged from 0 to 2 (Figure 3). Fifteen (45.45%) of the *CaDof* genes were intronless, whereas 12 (36.36%) of them contained one intron, and were generally located upstream of the DNA binding domain. Among them, six genes (*CaDof1, CaDof12, CaDof25, CaDof26, CaDof29*, and *CaDof30*) (18.18%) contained two introns.

### Phylogenetic analysis and classification of the Dof transcription factor family

**Figure 4:**
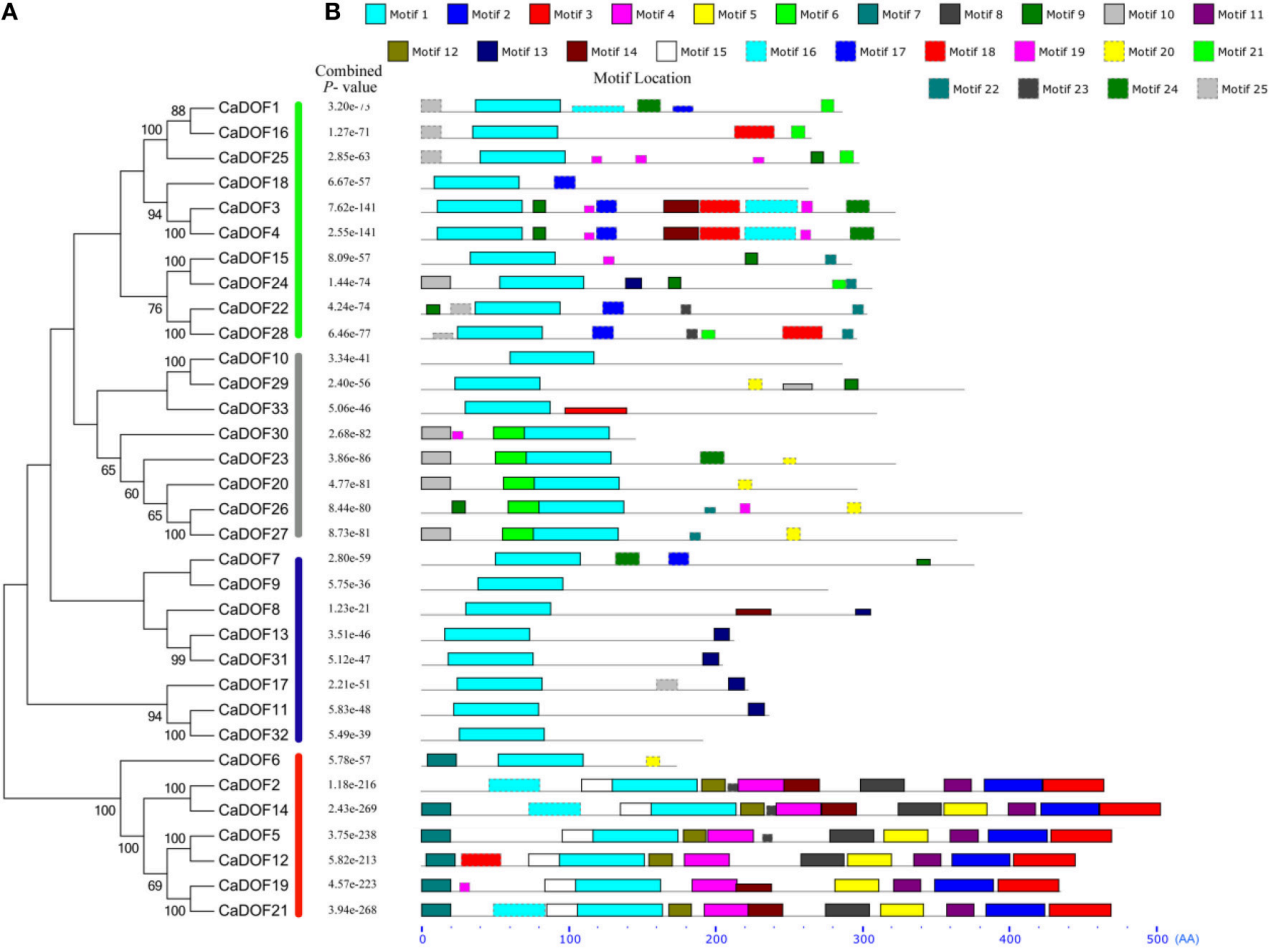
**Phylogenetic tree constructed by MEGA 6 (A) and motif locations identified by MEME program (B)**. Height of motif “block” is proportional to −log (*p*-value); combined *p*-values are shown in middle. Sequence logos and detailed information for each motif are shown in **(B)**.

In order to evaluate the evolutionary relationships among CaDof proteins, an unrooted phylogenetic tree was constructed based on the alignment of the full length of protein sequences by the neighbor-joining method with 1000 bootstrap replicates. Pepper Dof transcription factor family could be further divided into four major groups (from I to IV) with 50% bootstrap values (Figure 4A). Group I had the most members (10 genes, or 30.3%), followed by groups II and III, each comprised eight *CaDof* genes, whereas group IV contained the fewest gene members (7 genes, or 21.2%). Furthermore, some *CaDofs* within the same group shared similar exon-intron structure patterns in terms of intron number. For instance, almost all *CaDof* genes in group III had no intron, except for *CaDof7*. The two introns *CaDof* genes were mainly in group I (*CaDof* and *CaDof25*) and group II (*CaDof26, CaDof29*, and *CaDof30*). These similar structure features may be related to their functions in pepper genome.

To further reveal the diversification of *Dof* genes in pepper, putative motifs were predicted using the program MEME, and 25 distinct motifs were identified. The schematic distribution of the 25 motifs among the different gene groups is shown in Figure 4B. The identified multilevel consensus sequence for the motifs is shown in Additional File 2. Motif 1 observed in all CaDof proteins was the conserved Dof domain. As expected, members who had similar motif compositions could be clustered into one class, suggesting functional similarities among the Dof proteins within the same subfamily. Class II showed two conserved motifs (motif 6 and motif 10). Class III contained special motif 13. In class IV 10 motifs (9, 11, 16, 17, 3, 6, 5, 13, 2, and 4) were conserved, among which motif 22 might be characteristic of class IV. Motif 19 was the conserved motif in class I, with other variable motifs being 10, 15, 20, 12, 23, and 18. The motif distribution also confirmed that the *Dof* genes were conserved during evolution. The varieties of motif distributions in different subgroups implied sources of functional differentiation in *Dof* genes in the evolutionary processes.

**Figure 5:**
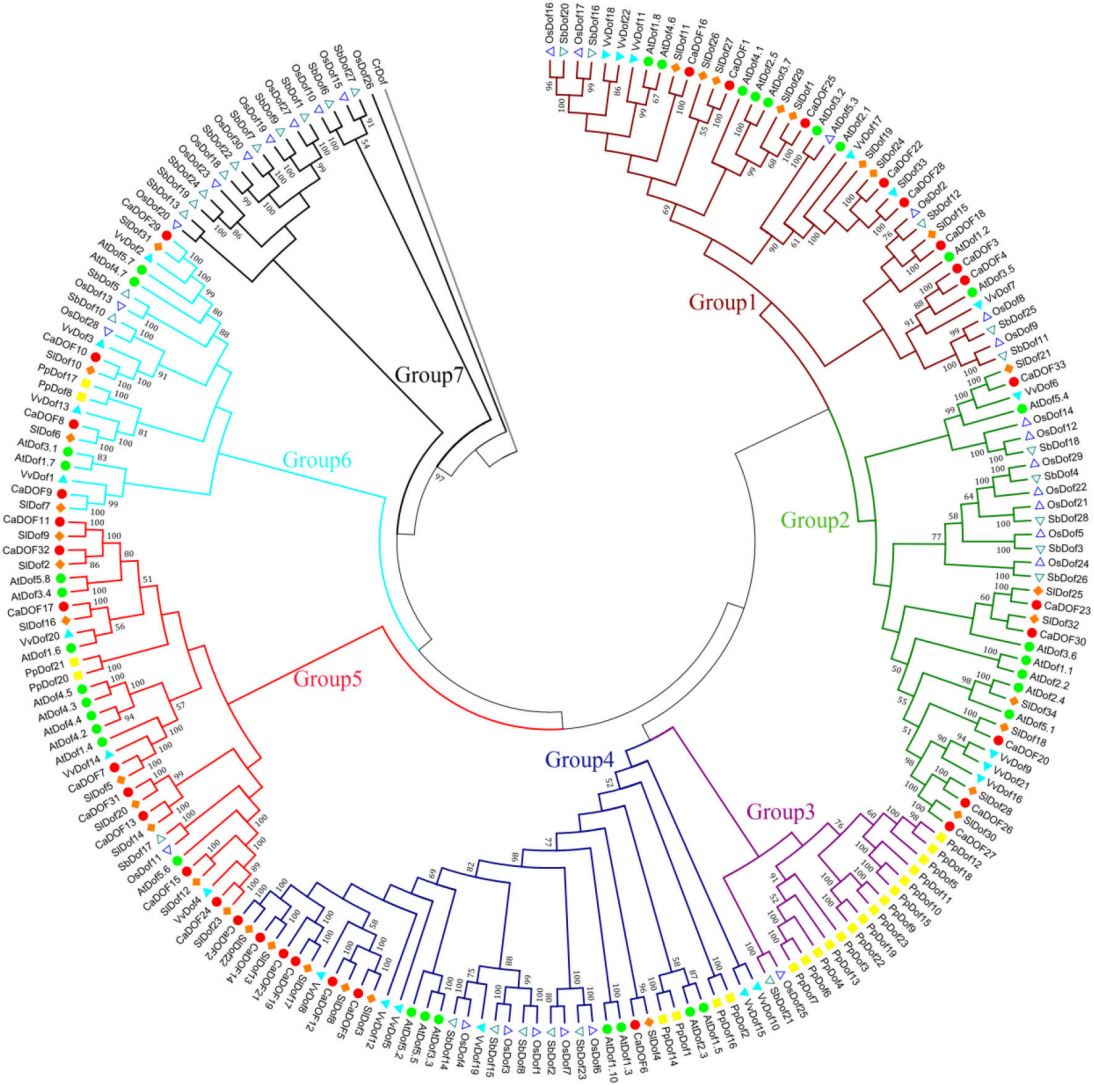
**Phylogenetic tree of the Dof transcription factors in eight representative species**. *Arabidopsis thaliana* Dofs (*AtDof1.1-5.8*) and *Oryza sativ*a Dofs (*OsDof1-30*) were obtained from Yanagisawa (2002), *Solanum lycopersicum* Dofs (*SlDof1-34*) were obtained from Cai et al. (2013), *Sorghum bicolor* Dofs (*SbDof1-28*) were obtained from Kushwaha et al. (2011). The putative orthologs from *V. vinifera, Chlamydomonas reinharditii* and *Physcomitrella patents* were assigned to corresponding CaDof proteins with the *E* < 0.01, which were extracted by a Blast P search from Phytozome v9.1 (http://www.phytozome.net/). The tree rooted using *CrDof* as an outgroup. These 207 Dof protein sequences (Additional File 3) were aligned. Bootstraping values are indicated as percentages (when > 50%) along the branches.

To further explore the evolutionary relationships within the pepper Dof family and those from other species, a total of 207 Dof protein sequences (Additional File 3) from eight species (four dicots including *Arabidopsis thaliana, Vitis vinifera, Solanum lycopersicum*, and *Capsicum annuum*, two monocots including *Oryza sativa* and *Sorghum bicolor, Chlamydomonas reinhardtii* and *Physcomitrella patens* subsp. patens) were used to construct a joint tree (Figure 5). The phylogenetic tree showed that *Dof* genes can be categorized into seven subgroups (groups 1–7) based on sequence similarities with a high bootstrap value (>50%). Among these, subgroup 1 constituted the largest clade containing 42 members, followed by subgroup 4 (40 members), and subgroup 2 (35 members); subgroup 3 contained only 17 members, which was the smallest clade. Subgroup 3 was primarily made up of the lower plant *P. patens*, whereas subgroup 7 contained the monocot-specific group of rice and sorghum.

The Dof phylogenetic tree also showed essentially the same clustering patterns in *S. lycopersicum* (tomato) and *C. annuum* (pepper), two members of the Solanaceae family. In total, 29 pairs of Dof proteins from *S. lycopersicum* and *C. annuum* were clustered as pairs, indicating that they might be orthologous. For example, the pair of SlDof31 and CaDof29 and another pair of SlDof9 and CaDof11 are highly similar, indicating that some consensus in domain may have existed before the pepper–tomato divergence. The same results appeared between *O. sativa* and S. *bicolor*, which is consistent with the notion that both belonged to the grass family. The phylogenetic similarity found in *S. lycopersicum* and *C. annuum*, and *O. sativa* and S. *bicolor* Dof proteins suggests that they may have evolved conservatively.

### Differential expression of *CaDof* genes in various tissues and developing fruits

In a previous study, we obtained a 90.5Gb Illumina RNA-Seq atlas of pepper genes from 46 libraries representing all primary developmental stages and tissue types, including various fruits in different developmental stages (Qin et al., 2014). Thus, we utilized these data to investigate the transcription levels of the *CaDof* genes in the root, stem, leaf, bud, and flower, as well as in the developing fruits of the pepper cultivar Zunla-1 (Additional File 4).

**Figure 6:**
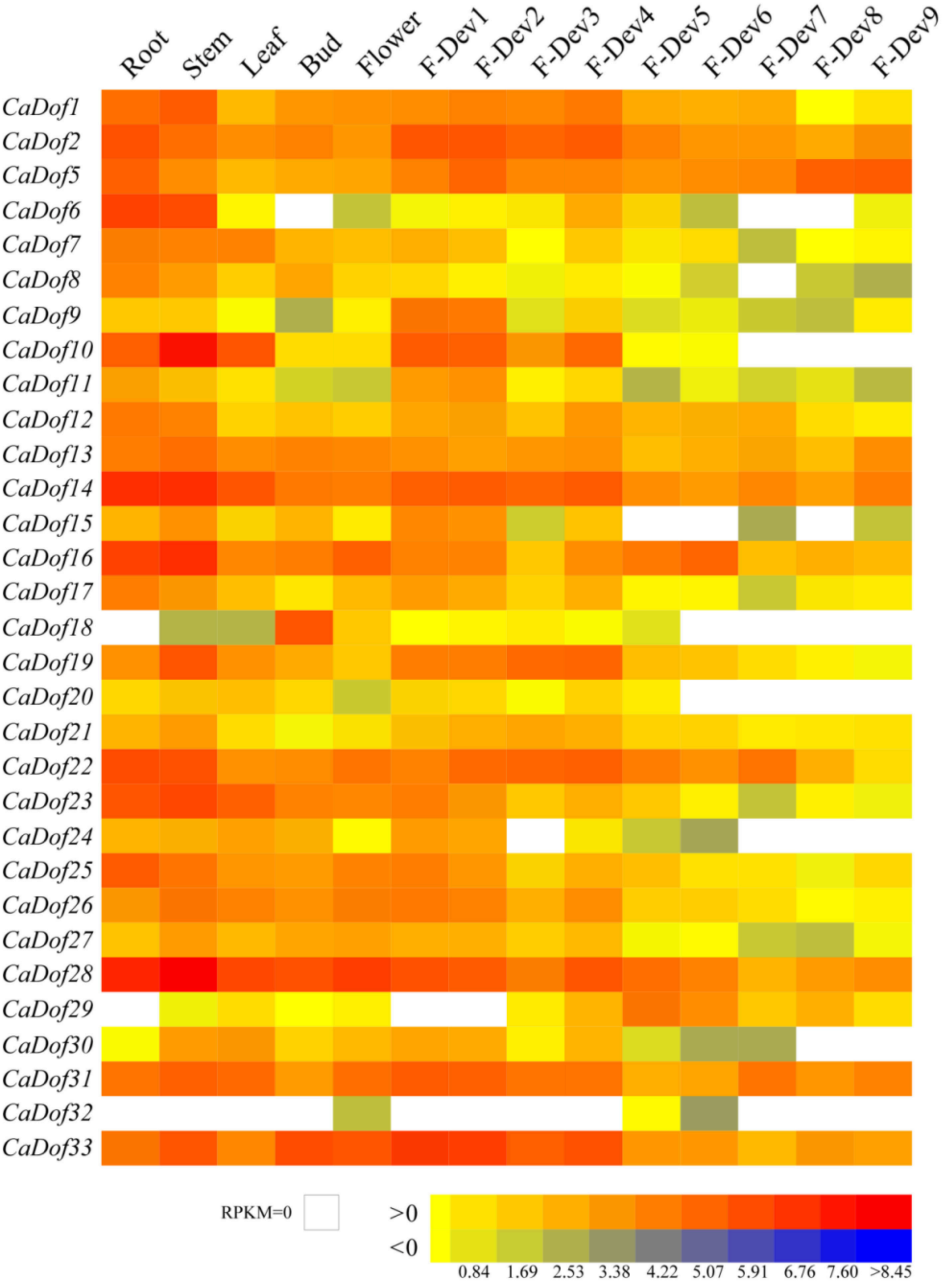
**Heat map representation of ***CaDof*** genes across different tissues and developmental stages**. The Illumina RNA-seq data were used to assess *CaDofs* transcript accumulation in total RNA samples extracted from root, stem, leaf, buds, flower, and fruit. The developmental fruit included nine stages, such as six pre-breaker stages (0–1 cm, 1–3 cm, 3–4 cm, 4–5 cm, and mature green fruit, Dev1–5), the breaker stage (fruit turning red, Dev6), and three post-breaker stages (3, 5, and 7 days after breaker, Dev7-9). The FPKM values were log_2_ transformed and heat map generated using BAR Heat Mapper Plus software. Bar at the bottom represents log_2_ transformed values. Genes highly or weakly expressed in the tissues are colored red and blue, respectively.

Among all *CaDof* genes, transcripts of 31 *CaDofs* were found in at least one of the 14 different tissues and developmental fruit stages. However, the other two *CaDofs* (*CaDof3* and *CaDof4*) were not detected in these RNA-seq libraries because they were at too low of a level. As shown in the heat map representation (Figure 6), most of the *CaDof* s exhibited divergent expression profiles in the 14 tissues examined. Most *CaDof* genes showed the highest expression level in roots and stems. Some *Dof* genes had very high transcript abundance in one or two tissues, but their expressions were almost negligible in other tissues. In particular, *CaDof6, CaDof14, CaDof16*, and *CaDof28* exhibited a higher expression level in the root, whereas *CaDof18, CaDof29*, and *CaDof32* could not be detected. Almost all *CaDofs* were expressed in the stem, except *CaDof32*, especially *CaDof28, CaDof10, CaDof14*, and *CaDof16* exhibited relatively higher transcript abundance. *CaDof10, CaDof14*, and *CaDof28* mRNA were highly expressed in leaves, whereas *CaDof18* and *CaDof32* had low expression. In both buds and opening flowers, *CaDof28* and *CaDof33* were dominantly expressed. Interestingly, the highest transcript abundance of *CaDof18* was in the bud, whereas in other tissues its expression level was nearly neglected.

In previous study, the developmental stages of pepper fruit were divided into nine stages including five color pre-breaker stages (Dev1–5, fruit size from 0–1 cm, 1–3 cm, 3–4 cm, 4–5 cm, and mature green fruit), the breaker stage (fruit turning red, Dev6), and three post-breaker stages (Dev7–9, including 3, 5, and 7 days after breaker, respectively; Qin et al., 2014). The expression pattern of *CaDof* genes during fruit development was analyzed. Most of the *CaDof* genes, including *CaDof9, CaDof10, CaDof11, CaDof19, CaDof23, CaDof26, CaDof27*, and *CaDof33* exhibited a gradually down-regulated expression profile during fruit development. During the fruit ripening stages (Dev 6–9, the color breaker stages), numerous *CaDofs* had very low expression or expression was negligible, including as *CaDof6, CaDof8, CaDof10, CaDof11, CaDof15, CaDof18, CaDof20, CaDof24, CaDof30*, and *CaDof32*. Interestingly, the expression level of *CaDof5* was up-regulated from Dev5 to Dev9. However, the relative mRNA levels of *CaDof2, CaDof9, CaDof10, CaDof15*, and *CaDof19* were significantly increased from flower to early fruit (Dev1) development, especially *CaDof9, CaDof10*, and *CaDof11*, and their expression levels we increased by more than 20 fold. These genes may be involved in pepper fruit formation. Therefore, further study of these *CaDofs* is vitally important and might be offer new insights into the understanding of the molecular mechanism of pepper fruit development and ripening.

### Expression profiling of *CaDof* genes under heat and salt stress

Functions of pepper *Dof* genes in response to abiotic stress are unknown. In order to elucidate the roles of *CaDof* genes in respone to abiotic stresses, we analyzed their expression profiles under abiotic stressors, including heat (38°C) and salinity (300 mM NaCl) treatment by using qRT- PCR in seedling leaf tissues. qRT- PCR primers are listed in Additional File 5.

**Figure 7:**
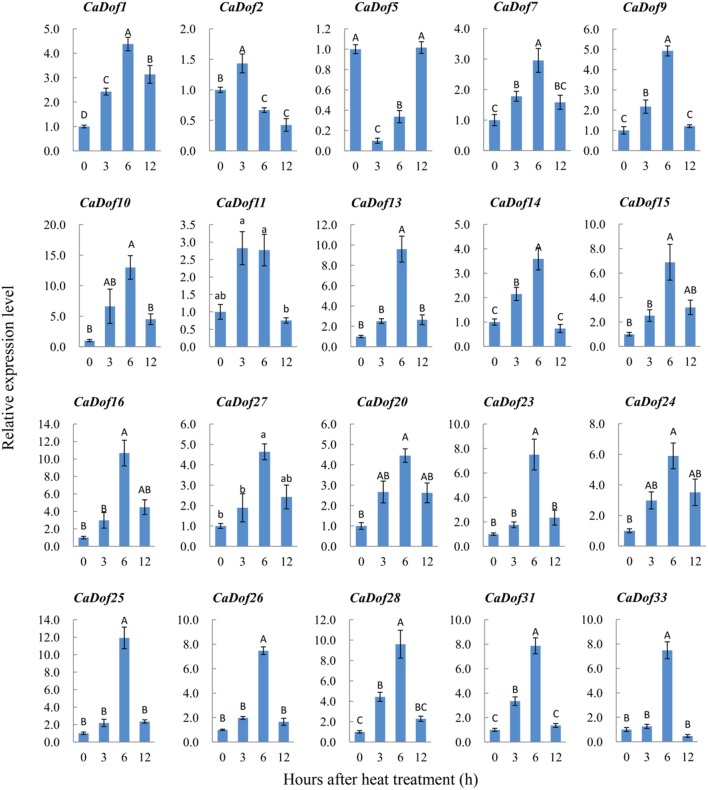
**Expression profiles of ***CaDof*** genes in response to heat stress treatment**. qRT-PCR analyses were used to assess *CaDof* transcript levels in the leaves sampled at 0, 3, 6, and 12 h after exposure to heat (38°C) in six seedlings leaves.

Among all *CaDofs*, we successfully designed and verified 20 primer pairs representing 61% of the putative *CaDofs*, except for *CaDof3, CaDof4, CaDof6, CaDof8, CaDof12, CaDof17, CaDof18, CaDof19, CaDof21, CaDof22, CaDof29, CaDof30*, and *CaDof32*. As expected, most of the verified *CaDof* genes were activated and showed higher expression levels by both stressors (Additional File 6). Under heat stress, 18 of the 20 *CaDof* genes displayed maximum expression at 6 h, and progressively declined thereafter until 12 h (Figure 7). Some genes were slightly up-regulated, such as *CaDof1, CaDof7, CaDof9, CaDof11, CaDof14, CaDof20*, and *CaDof24*, etc. Some were strongly induced, increasing more than 10 fold (such as *CaDof10, CaDof13, CaDof16, CaDof25*, and *CaDof28*). The greatest increase in expression (nearly 13 fold) occurred in *CaDof10* at 6 h of heat treatment. The results indicated that the expression of *CaDofs* were responsive to early heat stress.

**Figure 8:**
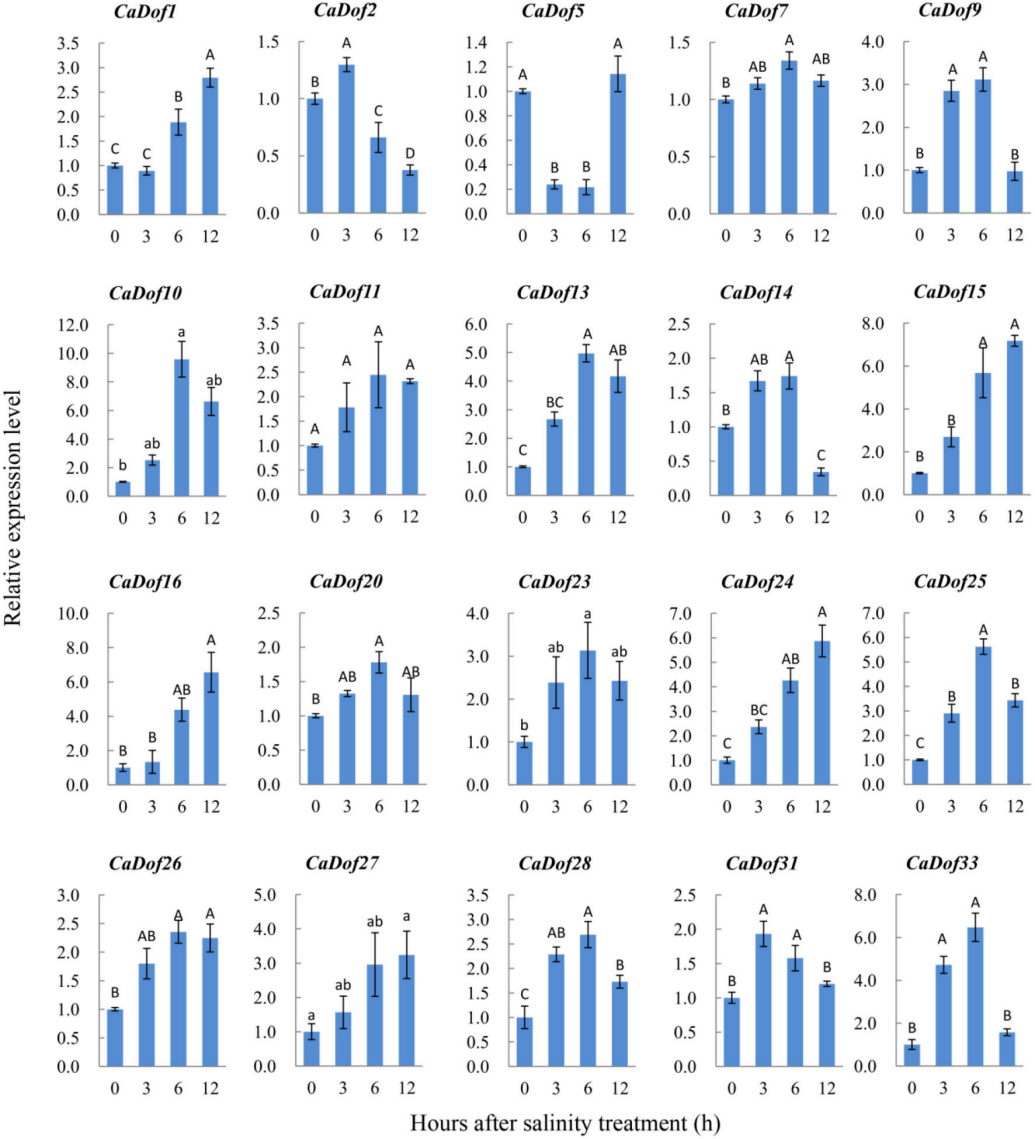
**Expression profiles of ***CaDof*** genes in response to salt stress treatment**. qRT-PCR analyses were used to assess *CaDof* transcript levels in the leaves sampled at 0, 3, 6, and 12 h after exposure to NaCl (300 mM) treatment in six seedlings leaves.

Similar to their response to heat stress, salt stress caused up-regulation of most *CaDof* genes. For example, *CaDof10, CaDof13, CaDof15, CaDof16, CaDof24*, and *CaDof25* were significantly up-regulated, whereas the transcripts of *CaDof1, CaDof9*, and *CaDof28* were slightly up-regulated (Figure 8). However, several genes, including *CaDof7 CaDof11, CaDof20*, and *CaDof26* were not sensitive to salt stress. Not all of the *CaDofs* exhibited high transcript accumulation under salt stress at 6 h. Two genes (*CaDof2* and *CaDof31*) and five others (*CaDof1, CaDof15, CaDof16, CaDof24*, and *CaDof27*) had the highest expression levels at 3 h and 12 h after treatment, respectively. It is worth mentioning that one gene (*CaDof5*) exhibited different responses to the two stressors. *CaDof5* was markedly down-regulated under heat and salt stress treatment at 3 h, whereas it was up-regulation again at 12 h.

## Discussion

Increasing evidence indicates that the *Dof* genes play important roles in a series of plant-specific physiological phenomena. To date, most of the research on the functions of the *Dof* genes has been focused in Arabidopsis (Yanagisawa, 2002; Lijavetzky et al., 2003), while it has been extremely limited in other non-model plants like pepper. In this study, we conducted a broad study of the *Dof* genes in pepper, including investigation of their structure, chromosomal organization, evolutionary relationships and expression profiles in different tissues and under heat or salt stress conditions. To our knowledge, this is the first comprehensive analysis of *Dof* genes in pepper plants.

### Characteristics of Dof family genes in pepper

This study revealed 33 potential *Dof* genes in the pepper genome. The number is quite conserved among *Arabidopsis* (36) (Yanagisawa, 2002), rice (30) (Lijavetzky et al., 2003), tomato (34) (Cai et al., 2013; Corrales et al., 2014), and potato (35) (Venkatesh and Park, 2015). However, it is significantly lower than that present in soybeans (78) (Guo and Qiu, 2013) and Chinese cabbage (76) (Ma et al., 2015). As we know, the genome size of pepper is almost quadrupled larger than that of tomato (3.26 GB vs. 850 Mb), and nearly seven times as large as Chinese cabbage (3.26 Gb vs. 485 Mb). Obviously, the number of Dof TFs in these species is not proportional with the genome size. This is because pepper did not undergo any whole genome duplication (WGD) after its divergence from the common Solanaceae ancestor (The Tomato Genome Consortium, 2012), confirming again the speculation that proliferation of TEs primarily contributed to pepper genome expansion (Qin et al., 2014). Besides, phylogenetic analysis showed that subgroup 7 clusters independently from those in monocot including rice and sorghum only, suggesting a potential functional diversity between dicot and monocot plants.

The intron-exon divergence was closely related to the evolutionary relationship of plants. The intron-exon organizations and intron numbers of Dof genes in pepper genome were quite similar to *Arabidopsis* (Yanagisawa, 2002), rice (Yanagisawa, 2002) and tomato (Cai et al., 2013). Moreover, the motif analysis indicated that the conserved C_2_C_2_-Dof domain was uniformly observed in all CaDof proteins, suggesting that CaDof TFs were evolutionarily high conserved in plants.

### Potential roles of *CaDof* genes in the tissue differentiation and organ development

Previous efforts have been made to explore the association of *Dof* genes with plant tissue differentiation and organ development (Gupta et al., 2015; Venkatesh and Park, 2015). In the present study, we firstly explored the expression evidence for all putative *CaDof* genes in different tissues (root, stem, leaf, flower, and fruit) by using RNA-Seq data. The *CaDof* genes showed differentially expressed in various analyzed tissues as reported in other plants (Cai et al., 2013; Ma et al., 2015; Song et al., 2016). *CaDof6, CaDof14, CaDof16*, and *CaDof28* exhibited relatively high expression levels in the root, indicating that they could play a role in the development of the plant root. Almost all *CaDofs* were expressed in the stem, especially *CaDof28, CaDof10, CaDof14*, and *CaDof16* showed predominantly higher transcript abundance. It is well known that roots and stems contained abundant vascular tissue. In *Arabidopsis*, more than half of the members of Dof family are expressed in the vascular system (Le Hir and Bellini, 2013). Among them, *AtDof2.4* and *AtDof5.8* were proposed to function in the early stage but different processes for vascular development (Konishi and Yanagisawa, 2007; Gardiner et al., 2010). The *CaDof28* homolog*, Dof5.6/HCA2* induces the formation of inter-fascicular cambium and regulates vascular tissue development (Guo et al., 2009). We infer that *CaDof28* could have similar functions during the pepper vascular tissue development.

Moreover, we identified one gene (*CaDof18*) that was preferentially expressed in early stage of flower. *AtDOF4.7* participates in the transcriptional regulation of floral organ abscission via an effect on cell wall hydrolase gene expression (Wei et al., 2010). Cycling DOF Factor1 (*CDF1*) had been demonstrated that may repress transcription of *CONSTANS* (*CO*) and thus show floral delay in *A. thaliana* (Lucas-Reina et al., 2015). In rice, 16 *Dof* genes were found expressing during the grain filling process also expressed at the flowering stage indicating that these genes are involved in the regulation of genes which are required throughout the seed development (Gaur et al., 2011). It may be conjectured that *CaDof18* might also play critical roles in floral development.

Fruit development and ripening is a complex and highly controlled biological process that is controlled by transcriptional regulatory networks involving many TFs, such as MADS-box, NAC, as well as EIN3/EIL, etc. (Feng et al., 2016). However, few *Dofs* had been well characterized their potential roles in this process. In banana, *MaDof10, 23, 24*, and *25* were ethylene-inducible and their transcript levels increased during fruit ripening. MaDof23 acts as a repressor and interacts with MaERF9 in regulating ripening-related genes (Feng et al., 2016). In potato, the *StCDF1* was reported to control the tuber formation by repressing potato *CO1/2* expression and thus allowing tuber induction (Kloosterman et al., 2013). In our study, the expression of *CaDof29* gradually raised from fruit developmental stage one to stage five, and reached peak in mature green fruit (Dev5), suggesting its importance in initial stage of fruit development. Its homolog, SCAP1 (*AtDof5.7*) has been proven to control the final stage of guard cell differentiation by regulating the expression of multiple genes responsible for stomatal maturation and function (Negi et al., 2013). Another gene *CaDof5*, being homologous to Arabidopsis CDF3, showed up-regulated during fruit ripening, especially in mature red fruit (Dev8 and Dev9). We speculate that it could involve in pepper fruit ripening. However, many others, such as *CaDof10, CaDof11, CaDof19, CaDof26, CaDof27*, and *CaDof33* exhibited a gradually down-regulated expression profile during fruit development. They might play a different role during fruit development.

### Potential roles of *CaDofs* in response to abiotic stresses

Previous studies have shown that *Dof* genes play important roles in plants in responding to various biotic and abiotic stresses (Noguero et al., 2013; Gupta et al., 2015). In *Arabidopsis*, the OBP2 (*AtDof1.1*) expression level increased two to three fold within 4–6 h after MeJA treatment and mechanical wounding (Skirycz et al., 2006). In tomato, *SlCDF1–5*, homologs of *Arabidopsis* CDFs, were reported to be differentially induced in response to osmotic, salt, heat, and low-temperature stresses (Corrales et al., 2014). In Chinese cabbage, most of the *BraDof* genes were up-regulated quickly by salt, drought, heat, and cold stress treatments (Ma et al., 2015). In potato, most of the *StDof* genes are up-regulated in various abiotic stresses including drought, high salinity, and ABA. Furthermore, *StDof* genes show either ABA-dependent or -independent expression pattern (Venkatesh and Park, 2015). In chrysanthemum, *CmDofs* showed either up-regulated or down-regulated when exposed to plant hormones and abiotic stress.

In the present study, the expression profiles of 20 *CaDofs* exposed to heat and salt stresses were also investigated by using qRT-PCR in pepper seedlings. Consistent with previous results, majority of the *CaDof* genes were responsive to these two stressors, exhibiting first increased and then decreased expression profiles. Interestingly, three homologs of *Arabidopsis* CDFs, *CaDof2, CaDof5*, and *CaDof14* showed different expression patterns. Maximum induction was observed under heat and salt treatment at 3 or 6 h for *CaDof2* and *CaDof14* respectively, with decay at 12 h. However, *CaDof5* was markedly down-regulated under heat and salt stress treatment at 3 h, whereas it got to peak again at 12 h. The similar result was confirmed in tomato (Corrales et al., 2014). These results manifest the potential positive roles of *CaDof* genes in plant adaptation to abiotic stresses; however, much more work is needed to verify their roles.

## Conclusion

In this study, 33 putative *Dof* transcription factor genes were identified in the pepper genome. Further, the 33 Dof transcription factors were characterized according to the conserved amino acid residues within the Dof domain, the conserved motifs and gene organization, phylogenetic analysis, and global expression profile among different tissues and under different stresses (heat and salt) by using high-throughput sequencing and qRT-PCR. The results obtained from this study provide useful clueto understand the molecular basis of the *Dof* gene family in the pepper and other plants in the family of Solanaceae. In particular, the expression profiling analysis of these genes will provide an important foundation for future studies assessing their functions.

## Author contributions

Conceived and designed the experiments: ZW, KH, and CQ. Performed the experiments: ZW, JC, XX, GL, XL, XC, and XT. Analyzed the data: ZW, JC, and CQ. Wrote the paper: ZW and CQ. All authors have read and approved the manuscript.

### Conflict of interest statement

The authors declare that the research was conducted in the absence of any commercial or financial relationships that could be construed as a potential conflict of interest.

## Supplementary material

Additional file 1 **The identified nucleotide and amino acid sequences of pepper *Dof* genes**.

Additional file 2 **Multilevel consensus sequence identified by MEME among *CaDof* genes in this study**.

Additional file 3 **The amino acid sequences of *Dof* genes in *Arabidopsis* (At), rice (Os), and tomatoes (Sl) used in phylogenetic tree construction**.

Additional file 4 **RNA-Seq RPKM expression matrix for all primary developmental stages and tissue types of Zunla-1**.

Additional file 5 **Primers used for qRT-PCR**.

Additional file 6 **The heat map representation of *CaDof* genes in response to heat and salt stress**.
