# Supplementary material for: Genome-Wide Identification and Expression Profile of Dof Transcription Factor Gene Family in Pepper (Capsicum annuum L.)
Source: Front Plant Sci. 2016 Apr 29;7:574. doi: 10.3389/fpls.2016.00574 (PMC4850169; doi:10.3389/fpls.2016.00574)
Supplement: Additional file 1 — The identified nucleotide and amino acid sequences of pepper Dof genes. [file DataSheet1.PDF]

Table S1 The identified nucleotide and amino acid sequences of pepper Dof genes.

The nucleotide sequences are as follows:

>CaDof1
ATGGATACTGCTAACTGGCCTCAGGAGATAGTGGTGAAGCCAATGGAAGAGATAATAGGTTCATCAAA
ACCTAATAATTGTGTTGAAAGGAAATTAGTTAGGCCACAAAAGGACCAAGTTGTGAATTGTCCAAGGT
GCAATTCAACAAATACAAAGTTTTGTTATTATAACAACTATAGTCTATCTCAACCAAGGTATTTTTGCAA
GACATGTAGAAGATATTGGACTGAAGGTGGATCTCTAAGAAACATTCCTGTTGGTGGTGGTTCAAGAA
AAAACAAGAAATCTTCATCTTCTAATAATAATAATAATAATAATAATTCAAATATTAATCATGTTGTAATA
AATAATCCTTTGATGAAAAAACTTCCTGATTTGATTGTGCCTCCACTAATACAACACGATATTGAGGAAT
ATCCAGAAAGGCATTTTGGAAATCGACCTGATAATTCTACAGTACTACCTCAACATCATCATGATCAAA
ACCCTAGAAAGATCATCCATGAAGGAAGCCAAGATCTCAATTTGGGATTTTCATCTGATTTCAAGACTA
TCACTGAGCTAATTCAGGTAGCAAATTACGATGGTGGTAACAAGGACAACAACAATAGCACGAATAAT
ATTTCTACATTGCCTCCTCCCTCTGAGCCGAGTTCACCTGCATTTTCGCAGTTGCCATCCCTAAATTTCT
CTTTGGATCATCATGGACTTGGAAATAATAATGTTCGTAGTGGCTATGGGTTTTTCTTTCCTTTTGTAGG
TCTAAAGCAAGTGTCAAATGCAAGTGATCATGTTCGAGATCAATCTACTAATAATGGATATTGGAATGG
CATGTTAGGAGGAGGAGGAGGAGGATCTTGGTAA
>CaDof2
ATGACGTGTGATTCAGAGATCAAGTTGTTCGGAAAGATACTTCCGGTGGTAGTCTCCGTCGAGTGTGA
CGTCGCCGGTGGGACTTCAAGTGGTGGTGATGGTGATCGATGTTTAGAGGATGGTAAAGCAAGCAGTG
CAGATGAAGGAAGTGAAAATGAAAATCAAGGAGCTGACAAGGACGATCTAACAGGAGAGCTCAATG
AAGCTAAATTTGAGGAGGGAGATCAAAGTCAGATGATGGAAGAGTCAGAAAATCCGAGGACTTTATC
AGAATCAGAGAACAGTTCTAAATCTCCAACTGATGAAGACTCTCAAGCTGTGAAAACATCCGGGACT
GAAAATGAACCAACTAATGTGACAAATTCTGAGCAGAACAGTCTAAAGAAGCCAGACAAAATTCTCC
CATGCCCTCGTTGCAACAGTTCAGATACAAAATTCTGTTACTATAATAACAACAACGTCAATCAGCCTC
GTCATTTCTGCCGGAGTTGCCAGAGGTACTGGACTGCTGGTGGTACCATGAGGAATCTCCCTGTGGGA
GCTGGTCGTCGCAAGAACAAGAATCTTGCAGCTCATTATCGTCATATAAGTATCTCCGAAGGATTACTA
GCAGCAGGAGTTGAATCTCCAAATGGATTAATTCATCATCCAATGTTCAAACCAAACGGCACTATTCTA
TCCTTTGGCACCGACTTGCCCCTATGTGAATCTATGGCTTCTCCATTAAGTCAAGCAGAGAAAAGGTTA
TCAAATGGTATCCAAAATGGTTTTCACAAAGCAGAACTCAAGAATTCTTCTTGCAAAGTTGGAGATAC
TGGGGATGAGTGCTACAAGGGGTCTAACATTCCGACTCCAAATGTGATGGTGGAAGAAGGTAAAAGA
GAGCTGCACAAGGCAGTTATGCATAATATAAATGGCATCCCATCTCCTTTTCCTTGCCTTCATGGAGTAC
CTTGGCCTTTTACATGGAATGCTGCTGTTCCTATGCCGGCTATTTGCCCCATTCCCTTCCCTATGCCTTGG
CTGGGTCCAGCTCTGCAAGCAGCAAGCGAGAAAACATCAGGTTCTGACCCTACTTCACCTTTAGGGA
AACATTCAAGAGAAGGGGATTTGCTTAAGCCAAGCAATCCCAGGGGCAAGGAACAATCAGAACAAAA
GTATTCAGAGAGGTCTATTTTGGTCCCAAAAACATTGCGGATTGATGATCCTGATGAAGCTGCTAAGAG
TTCTATATGGTCAACACTTGGGATTAAATATGATTCTGCCAACAGGGGAGAGTTTTTCAAGGCCTTGCA
ACCAAAAAGTGACGACAAACACAACAAAGCCAATACTCCTCCAGTGTTGCATGCTAACCCTGCAGCT
TTATCTAGATCGATTACCTTCCAACAAAGTGCCTAG
>CaDof3
ATGGAAAGAGGGACAATATGGAAACCCAATGTTGAGTTAGCACCAGCATGTCCTCGTTGTGGTTCAAC
AAATACAAAATTTTGTTACTACAACAACTATAGTTTGACACAACCTAGGTATTTTTGCAAAGGTTGTAG

AAGGTATTGGACTAAAGGTGGTTCTTTAAGGAATGTTCCAATTGGTGGTGGATGTAGAAAGAGTAGAA
GAGGTAAGTCTTCTTCTAATACAATTCATATTCATCATCATCATGAACTTATTTCAAGAAATTTGGGACAT
GGGGTTTGTTTAAATCCTACTAATATTGATCATCATAATCAATCTACAAGTAGTTCATTGGATCATCATCA
TGGTCCAAGTATTGATCTTGCTTTAGTCTATTCAAATTTCTTGAATAGTACTAATTCCAAGTCATCTCAGC
CGGAGGATCGTCAGAATCCTGAACTTCCTGACGATTTGTTATTGCCTGATCAAGGAGTACTCGTCACTC
CATCATTCGAGCTTTCAAGCATGATAGACATGGAGTTTGTGAATAGCGAATTAGGTCAAGAATCGCGAT
TACTTGGTGCTGGTGCTGGTGATGGTGTTGATTTTTACTTTAGTGGTATTCATGAGGAGAAGCAAAATG
GTATGAATCATAGTGATGTTCATGATGATCACTATACAAACAATATGAATGTGAATGCCAATAATAGTATC
AACCATGATCATCAATTAGGCAACAATAATTATATGGACCTCCCTCCATTGCCTTGTGAAGAGATTATGT
GGTCTAATTCTCATGATCATCATCATCATATGGTATTTCCTAATGATCTTCTACGTACAAGTCATAATTTAA
CAACCGGAGGATCAGAGCCAGAACCAGAACCAGAATCAGCAGTACAAAATCCGAGTCATGATCATAG
CGCGAATAATGCAAATGATGGGAGCCTTTTCAACTTGTCCAACTTTGGAAATATTTTCAGGCCTTGA
>CaDof4
ATGGAAAGAGGGACAATATGGAAATCCAATGTTGAGTTAGCACCAGCATGTCCTCGTTGTGGTTCAAC
AAATACAAAATTTTGTTACTACAACAACTATAGTTTGACACAACCTAGGTATTTTTGCAAAGGTTGTAG
AAGGTATTGGACTAAAGGTGGTTCTTTAAGGAATGTTCCAATTGGTGGTGGTTGTAGAAAGAGTAGAA
GAGGTAAGTCTTCTTCTAATATAATTCATAATCATCATCATCATGAACTTATTTCAAGAAATTTGGGACAT
GGGGTTTGTTTAAATCCTACTAATATTGATCATCATAATCAATCTACAAGTAGTTCATTGGATCATCATCA
TGGTCCAAGTATTGATCTTGCTTTAGTCTATTCAAATTTCTTGAATAGTACTAATTCCAAGTCATCTCAGC
CGGAGGATCGTCAGAATCCTGAACTTCCTGACGATTTGTTATTGCCTGATCAAGGAGTACTCGTCACTC
CATCATTCGAGCTTTCAAGCATGATAGACATGGAGTTTGTGAATAGTGAATTAGGTCAAGAATCGCGAT
TACTTGGTGCTGGTGCTGGTGATGGTGTTGATTTTTACTTTAGTGGTATTCATGAGGAGAAGCAAAATG
TTATGAATCATAGTGATGTTCATCATGATCACTATACAAACATCAACGTGAATGCCAATAATAGTATCAA
CCGTGATCATCAATTAGGCAACAATAATTACATGGAGCTCCCTCCATTGCCTAGTGAGGATATTATGTGG
TCTAATTCTCATGATCATCAGTATCATCATATGGTATTTCCTAATGATCTTCTAAGTACAAGTCATAATTTA
ACAACCGGAGTGTCGTCAGAGGCAGAGCCAGAACCAGAACCAGAATCAGCAATACAAAATCCGAGT
CATGATCATAGCGCGAATAATGCAAATGATGGGAGCCTTTTCAACTTGTCCAACTTTGGAAATATTTTC
AAGCCTTGA
>CaDof5
ATGTCAGAACTCAAAGACCCTGCTATTAAACTCTTTGGCAGAACCATTCAGTTGCCGGATGTTCCAGAT
TCTTCTGAAACTATGCTAGAAGATTCTTTGCCCGAGGAAGCCAATGGAGAAGAAGAAGATGTAGAAGA
TCAAAAGGATAACATTGGAGGAAACCTGGATGATGAAGAGGATGAGATGGAAATTTTGACTGGAAAG
GAATTGCAGGATCAGAATTCACATCCAACTAAAACTGATAGTATAAAGGTGCAACCTGTTGGTAGCGA
CTGTTCAACAAGACCTTCAAAAAGTGAAGAAGAGCAAGGAGAAGCAAGTAATTCGCAAGATAAAATC
CTCAAAAAGCCAGACAAGATACTTCCATGTCCCCGGTGTAACAGCATGGAAACCAAATTTTGTTATTTC
AACAATTACAATGTGAACCAGCCTAGACACTTCTGCAAGAATTGCCAGAGATATTGGACAGCTGGTGG
GACCATGAGGAATGTGCCTGTAGGTGCTGGTCGTCGGAAAAACAAGAACTCAATTCCACATTACCGTC
AAATATCTGTCTCTGAAACACTTCCGAGTGCTCAAGCAGATTATCCAAACGGAATCCAACAACCTGTT
CTTGCGTTTGGCTCCCCTACACCACTCTGTGAATCAATGGCTTCAGTTTTGAACATTGCCGACAAAACA
ATGCATAATTGCTCACAAAATGGGTTTCATAAACCACAAGATCCCGGGGTTCCAGTTAGTTATGGAGCT
GGAGATAATGGAGATGATCATTCCAGAAGATCATCAGTCACTACTGCAAATTCAGAGGATGAAGTTAG
CAAAATTGTACCGGACCAGCTAAAGAACTGCCATAACTTTCCACCTTACGTGGCTTGCTATCCTGGTGC
TCCTTGGCCATATCCATGCAATTCTGTCCCATGGAGCTCTGCAGTCCCTCCTCCTGGTTATTGCCCTCCT
GGCTTTCCTATGCCGTTTTACCCAGCAGCTTCATATTGGGGTTATACCGTAGCAGGTTCTTGGAATGTTC

CTTGGATATCCCCAACAACTGGTTCCCTAATTCAAACACCTCCGACTTCTGGTCCTAATTCTCCAACTCT
AGGGAAACACTCAAGGGATGAAAACATACTAAAACAGCTGAGCAACAAGGAAGAGTCTTCAAAAGA
GAATAATCCTGAGAAGTGCCTCTGGGTTCCAAAAACTCTGCGAATAGACGATCCAAGAGAGGCTGCC
AAGAGTTCTATATGGGCGACATTGGGAATAAAACATGATAGTGTTGAGTCAGTTGGTGGAAGTCCTTTC
AATGCTTTTCAGCCGAAGAATGATGACAATATTAGTGTTTCAGAAAACTCTACTGTATTACAAGCAAAC
CCAGCAGCATTGTCTCGGTCAGTAAATTTCAATGAGAGCTTATAA
>CaDof6
ATGGCTGAAGTTCAAGAAAGTCGCATCTCACAAGGCATCAAGTTGTTTGGTGCAACAATACAAGTCCA
AGAAATTAAGCAAGCCAAAGTTCATCAACCAACAAACAAAGTTGATCAAGATCATGATAATAATAATA
ATGATCAAGAAAAAAGGCCAGACAAGATCATCCCTTGCCCTAGATGCAAAAGCATGGAAACCAAGTT
TTGTTACTTCAACAACTACAATGTTAACCAACCAAGACACTTCTGCAAAGGCTGTCAGAGATACTGGA
CGGCCGGTGGGGCCCTACGGAACGTGCCCGTAGGAGCCGGCCGTCGCAAGGCCAAGCCACCATGCGG
TCCCGGCCCGCACGGCGACATGAACGGGCTTTCAGATGGTTGCTTTTTTGATGTTACTAATCATCATGG
CAATAATAATAATATTCATCAACTTGAGTTTGACGGTGTGGTGGCTGAGGAAGATCAATGGCATCTTTTT
CAGGCAGCAAAGAGGAGGAGGAGCACCTCCCATACCCAATCTTGTTGA
>CaDof7
ATGGCACTCATCCCTTCTTCCACTACTAATGAAATATGGCCACAGATAGATGAGAAAAATAACTTGATG
ATGGCCTCAAATGGTAGCAGTAGTAGTAATACTAGAGATATGGAGAAGCCAATTCCAGATCCATCACAA
CAACCACCACCACCACCACATCTGAAATGTCCTCGTTGCGATTCGTCTAATACGAAGTTTTGCTACTAC
AACAACTATAGTTTGTCTCAGCCAAGACACTTTTGCAAGGCATGTAAAAGGTATTGGACTAGAGGAGG
AACCTTAAGGAATGTACCAGTTGGAGGTGGCTGTAGGAAGAACAAGAGGATTAAGAGACCATCAACT
AATTCTTCTTCTTCTTCTTCTTGTACTGCTCATGATATAATTACTACTTCAACTCCAAATATCTCTACTTTA
AACCCTAGCCACCATGTAGCCCATAATAGTATTGATATATCTTCTACAAATTCTATCAATCCTTTATTTTAT
GGGTTGACTAGTGAGAGGTCTGATCTCAACATTCCATTTGCAAGGCTTTTCAATTCTAGGGTTTCGAGT
CATGCTACAGTCGGTGAGGGACAAGTGTATTCTCTGACAGACAGTATCCCTGGATTAATGGATCGTCGT
ATGGGGTTAGGGTTTTCTAATTCTTCTGTAGGGGGGGTCAATATTATGGGTGAGAATAATAATTATGGGC
ATGGTGGGTTCAATCCTATTAAGCAAATCCAAGATGTTGTCATGACTAGTAATTGTACTACTTCTTCAAC
TTCACTTCTTTCAACCTACCCCAATATGTTTGGATCTTCAACATCAACTTCAACTATGGCTTCTCTTATAG
CTTCAAGCCTTCAGCAACAAAAGTTCATGTCCAATATTAATGGTAACAATTTTCACAACTTGGCTCCTA
ATTATGAGGAATTGCAAATGTCAAGGGGGGACAACAACAACAACAACAGTAATGTTCATGAAGGTGG
TGGAAATGGGATCACAATGTTGAAAGCTGAGAAAATGGATCTTTCAAATCATCAGATTCATGAACAAA
TTATTAATTCATCTGATCCTTCACTTTCTTGGAATGGTGCTTGGCTTGATCCTTCTAATATGGGGTCTAAT
TCAGTCCCTTCTCTCATCTAG
>CaDof8
ATGGAGCAAGGTGGAAGATCGTCAGGAGAGAGCGATCGAAATCAGCAGCAAAGGAGAATGAAGATG
CCTGAAAACAACTCATCACAACCACAGCCGCCACCGCAGAAATGCCCTCGTTGTGATTCAAACAACA
CCAAGTTTTGCTACTACAATAACTACAGTTTGACTCAGCCGAGGTACTTTTGCAAGACCTGTAGGAGGT
ACTGGACTCAAGGTGGAACCTTGAGGAATGTCCCCGTAGGCGGTGGCTGCCGTAAAGGTAAACGCAC
GATGAAGGGCGGTAGCGTTGGTGTTAGTTGTGGTTCATCTTCATCAGCTAGTGAAAGTTCATCAAGATC
TTATCAACAACAATCGCAACAAATACCGAATTTGTCAGCAGCAGCAGCAGCTGTTTTTTTCTCAGGTA
ACAACAATTCGAGATCTCAACCGCCGCCACTCCCATCGCTGAGTTCTTTGTATACCGGTGGCGTTGGTG
GTGGTGGTGGGTTTTTATCTACTTTAGCCTCCATGCAATCAATGACTCAATTATCACAAGGTGTCAATAA
TGATCACTCTCAGTTAGGGGTAATTTCAGCCAGCAATAGTAGTCAATTTGGTAATTTCAATATTCCAAGT
AGTATTCCTCCTAAGGTTCAAATTAACCAACAAATGGAAAGTGGAATTTACCAAATGGTCGTCAATAGA

GAAAAGCCCATGGAATCATCTTTTTACCCATCTGATCAAATTTCACAGTTTCAACCAACAAGGCCTTTG
GGTTCTTGGACACAAAGGTTCATCAACAACAACAATAACAATAATATTTGGCCGAACGCCTCCGCTAG
CAGTAGCAGCAGCGGAGGTGCTAACAGTAGCACCACCGCTGCTGGTGCATCTCTCAACCCGAATCAAT
GGCCTGATCTTCCGGGGTTCGGACCATCACCATGA
>CaDof9
ATGGGGTTGAGTACTAAGTTGGTTTCAATTGATGATGATGGTCTAGATGATTGGACTTGTAGCAGCCAG
AATTCTCTACCGGAACCACCGTTGATAAGGCGGCAACCGCCATCGAAACCGGAGCCATTGAAATGTCC
AAGGTGTGACTCAATCAACACAAAATTTTGTTACTACAACAATTACAACAAGTCACAACCTCGACATT
ATTGTAAAGGTTGTAAAAGGCATTGGACCGAAGGTGGCACCCTTCGTAATGTCCCGGTAGGCGGTGGC
CGAAAAAACAAGCGAATGAGGATGACTACTGATCTCGTTGATCATATAACTGGAAGAAAACGTGTCAC
CTTAGAGGAGATGAACGATCAGAGGTGTCCTTTAATCAGCACAACCATCACTAATACTACTACAAGTTC
CATGCCAAGTACTATTATTAGTAATATGGATGAAGATATCAAAAATATTCCTTCATTAGCATCATCGTCAC
TACCATATGACATATTTTCAAGTTTAAAGTTGTCATCAATTCCTCAAGATGGGAACACACATTTTTCACT
AATTCCCAATTCTAGTACTACTCAATTATCTTCAAATGTGTATTGTAATTATGATTACATGGGGAAGTTTG
ATAGTACAATGGAGGAGTCAACAATAACTACAGTCATGCCAATTACAAGCAGCAGTGATCTATTTTCTC
AGCCATGGAAAGTTCCAGAAACAAGCAATGACTTTATTATTGAAAATATGTCAAGTAATTATTGGAATT
GGAATGAATTCGACACGTTGAGTACTGCAGCTGATCTCAATATACAATGGGATGATTTGGAGATCAAAC
CATAA
>CaDof10
ATGATTCAAGAGCTCTTCGCCGGAAACACTACACTTATAGGAGGAGATAATAATATTTCAAAGTTATCTA
ATATTACTCCTTCCTCCTCTCCTCTTTCTTGTACTACTTCTAATTCCAGTATTGCCCCTGCAGCAGCTGCT
GGCGCAACTGCAACAGCAAATGCAAGTTCCCCTTCGAACGTGGAAAGCCTTAGATGCCCACGTTGTG
ATTCCCCAAACACAAAGTTTTGTTACTACAACAACTACAATTTAACTCAGCCTCGTCATTTCTGCAAGA
CTTGTCGCCGTTATTGGACTAAAGGTGGCGCGTTACGCAACGTTCCTATAGGTGGTGGCTGTAGAAAA
AACAAGACCATCACTACAGCTAAGTCAAGTGCCGCAAAATTGAAAAATTCAATTCCATTTGAGTTTATT
GGAAAATCAGGCATTTTTGGAGGGTTCGAGCAGGAAATAATACCTTCTAATAATAACCCTTTCCTATTCT
CCACACCCCATCAAAATCATAATCCTATTCTTTCCTTACTTAGAGGAAATCATCATAACCTAAACCTTGT
CAAGGATGAGCAAAAATCAATTGAGGTGAATTTACATAATCAGTTTCCTTCCAACAGTCTTTCCAGTTT
GTGGAAAAATAACGATACTATAGTTGGTGAAGTTCAAAATAGTACAGGGTTTCAAGAACTATATCAAAG
GCTTAAAGCTTCAACAAGTAGGTGTTATCCCGATATTCATGGACCATCATCATCATCATCGTCCTCAATG
ATTTTGGAATCGGCGCCGGTGGCCGGAGGAGAATTGGGTTTCTGGAGCCCTAGCTTTTCGACATGGAC
GGATCTACCCACAGCAAATAGTGCATATCTCTAA
>CaDof11
ATGCCTTCCGATGTTAATGAGCGAAGAGTTACCAAGCAACAGCAAGGAGGTGCACCGGCTCCAGAGC
CTGAGCATCTTCCTTGTCCACGTTGTGATTCCATCAACACCAAATTCTGTTACTACAACAACTACAATTT
CTCTCAGCCACGTCACTTCTGCAAAGCTTGCCGCCGTTACTGGACACACGGCGGAACTCTTCGTGACA
TCCCTATTGGGGGAGGAAGTCGCAAAAATGCCAAGCGGTCACGTACGATCACAACTAACAGCAGTTT
GTCCTCCACGCTCTCTCCTCGAGATTACCAACACGCGTCTAACCCGTCGGCTTTCTTGGTTCCTTTAAC
TGCCGATCATGGGGGTTCACTACCCTTCGACGTGAAGCCGAATGTGAACATGTGTGGGAGTTTCACTT
CGTTGTTGAGCAGTGCTCAAGGGCCTGGTGGGCTTTTAGCACTTGGTGGATTTGGGCTTGGAGTTGGC
GTTGGGTCTGGAATTGAAGATATGGGCTTTGGTCTTGGCAGGCCCATTTGGCCGTTTCCTGGAGTTTCA
CATAGCAATGTTGAAAACAACAGCGCTAATGGCACTGGAGCCAGTATGTTGGGAAGCACGTGGCAGC
TTGCTAGTGGAGGAGAGGGTGGCTTCGTTGGCGCAACAGCAGGGGAAATTTTTAATTTCCCTGATCTT
GCTATTTCTACCCATGGAAATCGGATGAAATGA

>CaDof12
ATGTCTGAAGCAATAGCTAGTAGGGACCCTGCCATTAAACTCTTTGGTCGGACTATTCACTTGCCCCTT
TTTCCGGCGCCGGCGCCGGAAAATACCGGATATTATTGTTCCTCAGCTGGAGAAAATGAACAAAAACA
CGAGGATCAGAATCCAATTCAACAGAAATGTGATATTACAAAAGAGTTACCTGATTATTATGACTGTTC
AACAGCCAAAACGTCAAAAAGTGAAGAGGAACAGGATGAAACAAGCAACTCACAGGAAAGAAACC
TTAAAAAAACAGACAAAATACTTCCATGTCCTCGCTGCAATAGCATGGAAACAAAATTTTGTTACTTCA
ATAATTATAATACTAGCCAGCCTAGGCACTTCTGCAAGAATTGTCAGAGATATTGGACTGCTGGTGGGA
CTATGAGGAATGTGCCTGTAGGTGCTGGTCGTCGAAAACACAAGAACTCAGTTTTGCATGACAGTTAC
AGTTCTGTTTCTGAAGCACTATCAAAAGCAAGAACAAATTTTCCTAATGAAACCCAGCAACCTCCTCT
CACGATCAGTGGAACTATTCTTACATTTGATACTGACAAACCCCTATCGGAGTCGATGGTTTCAGCTTT
GAACGTTTCTGATAAAACTATGCAGAATTATTCTGGGAATGGGTTCCGCAAATATAAAGAGCTTGGAAT
TCAAGCTGGAGATAAAGGAGATGATCTTTCCGATGGATCTTCAGTTACTGTTGTAAGTTCGAAGGATAG
TGATAATGGGTTGCCTGACACACTAAGGCAGAATTGTAACAGCTTTTCAAATCATTTACCTTGCTTTTCT
GGAGCTCCTTGGCCATACATATGGAGTTCTGTGCCCTGTAGAAATGTAGTACCTCCACCTGGCATTCCT
GTGTCATTGTTCCCCGCAACCACTTATTCGGGTTGTACAATACTTGGTTCTTGGAATGTCCCTAAGATGC
CCCCACCCACTGCATCCCAAAACCAAGTGCCTCTAACTTCTGGTCCTAATTCTCCAACTTTTGGGAAAC
ATTCAAGGGATGAGAATGTGCTGAATTCGATGGGCACTGAGGAAGAGCCACGAAAAGAGAGTAATCC
TGGGAGGCGCATATGGTTTCCTAAAACATCGCGAATTGGTGATCTAGGAGAAGCAGCAAAGAGTTCTA
TCTGGGGAACATTGGGAATAAAGCATGAGGTAGTTGATTCAGTTGGTGGAGGTCTTCTCAAAGCCTTT
CTTCCAAGGAGTGATGAGAGGAACTGTGTTTCAGAGACCTCTACTTTATTACAAGTCAATCCAGCAGC
AATATCTAGGTCATTAAATTTTAATGAGAGCTCCTAA
>CaDof13
ATGCAAGACCCATCAATTTATTCACAAATCAAGCCTCAATTCCCTGAACAAGAACACTTGAAATGCCCT
AGATGTGATTCACCAAACACAAAATTCTGCTACTACAACAATTACAACCTTTCTCAGCCACGCCACTAC
TGCAAAAGCTGTCGAAGGTATTGGACTAAAGGCGGTACTCTTCGTAACATCCCAGTTGGTGGAGGTTC
TCGTAAGAACACGAAACGATCATCTTCAGCATCAACCAGTAAGAAAATTACCTCAACAACAACAACA
ACTCCACTAACATCATCTGTTTCAGCTTCTTCATCAGCAAATCCAAAACCAGAGCCATTTGGTATACCT
GCAATTCCGTCTTTTGACGTGACTACTGGTCCATTCAGCTCACTGTTAGCGTCAAACGAACCGCAATTT
GGGAATTTGCTGGAAGCTTTGAATCCGAATAATAGTAATAATAATGGTTCCAATATTCAGTTGAGTGAAT
TTTCAAGGAATCCAATTTCCAGTTCGGGCTTGGGCTTGGGTTCGGGTTCGGGCTCCGGCCAGAACCAT
TCAAATGGTGGAGAATCAAATAATTGTTGGAATGGTGGTAGCAATGGTTGGCCTGATCTTGCAATTTAC
ACACCAGGTTCTAATTTCCAATAA
>CaDof14
ATGAGAGAAGTGAAGGACGGAGAGATAAAGTTATTTGGAAAGAAAATTGCGTTGCCGGAGAACGGG
AAGATGCTGCCGGTGATAGTTTCCGGTGAAGATTCCGATGTCGGGAAGTCTGTGAGTGGTAGTGAGGT
TGTTACGGGTGAAGAAAGTAGTACCGGGTCGGATCGTGGTGATCCATGCTTAGTGGATAAGGAAGGAA
ATACTTCTTCTGAATCTGATGGTGGAAGTGAATATGAAAAGGAAGACGCTGATAAGGATCAAATGACA
AGAGAGCTTAGTGAAGCCAACTTAGAGGAGAAATACCAAAGTCAAATTATGGAAGAATCAGAAAATC
CGAAGTCTCCATCAGAAAACAAGTCTAAAACTACTACCGATGATGACTCTCCCACGGCAAAATCATCC
AGGACTGAGGGTGATCAAAATGATGCAGCCGCAAATTCCCAGCAGAAACCTCTGAAGAAGCCAGACA
AAATTCTCCCCTGCCCTCGTTGCAATAGTATGGATACGAAATTCTGTTACTACAATAATTACAACATCAA
TCAGCCTCGTCATTTCTGCAAGAGCTGCCAGAGATATTGGACAGCTGGGGGTACCATGAGGAATGTGC
CTGTGGGAGCTGGTCGTCGCAAGAATAAGAACTCTGCATCGCATTGTCGTCACATCATGATTTCTGAAG
CCCTTGAAGCTGCAAGAATTGATCCTCCAAATGGATTCCATCATCCAGCATTTAAACCCAATGGCACTG

TCCTATCGTTTGGTCCTGACTCGCCACTGTGCGACTCTATGGCGTCTGTTTTGAATCTTGCTGAGAATA
AGACACCAAATGGGATCCGAAATGGTTTTTACAGACCAGAACACAAGAATCCATCTGGCCTAGGTGGA
GAAAATGGGGATGACTGCTCTAGTGGTTCTTCAGTCACCACTTCAAATTCAATGGCGGAAGGAGTAAAA
AAATCGCGCCCCTGAGGCAGTTATGCAAACTATAAATGCCTTCCCATCTCCAGTTCCTTGCATCCCCGG
AGTACCTTGGCCTTTCCCATTTGCTGCTGTTCCTTTCCCCGCAGTTAGCCCCTCTGGATATCCTATGCCT
TTCTGCCCTCCACCACCTTATTGGAATTGCAGTGTGCCTGGTCCATGGAGTCTTCCTTGGTTGACTGCA
CCTTCACCAACAGCAAACCAAAATGGATCAGGCTCTGCTCCTAATTCGCCTTTAGGGAAGCATTCAAG
GGATGGTGAATTGCTTAAGCCAAACAATCCCGAGGGTCAAAAGAACTCAGAGGGGTTTGTTATAGTAC
CAAAAACATTGCGGATAGATGATCCTGATGAAGCTGCAAAGAGTTCTATATGGTCAACACTGGGAATC
AAGTACGACTCTGTTAGCAGGGGAGGACTTTTCAAGGCCTTGCAACCGAAAAGCAGTGAGAAGGATC
ACCCTGCCACTACATTCCCGGCTTTACAGGCTAACCCTGCAGCCTTTTCTAGGTCCCTCAGCTTCCAGG
AGAGAGTCTAA
>CaDof15
ATGGATTCTTCATCTTCACCATCTGGTGGGGATCATCACATTAATCTGACATGTTCAAGGCCCATAATAG
TTGATCAAAGAAGACTAAGACCCCCACATGACCACTCCATCAAATGCCCTCGTTGCGACTCTACCCAC
ACCAAATTTTGTTACTACAACAACTATAGCCTCACTCAACCTAGGTACTTTTGCAAGACATGTCGAAGG
TATTGGACTAAAGGTGGCACTTTAAGAAACATCCCTGTTGGTGGTGGCTGCCGTAAGAACAAAAAAGT
TTCCTCCAAAAAATCTAACACTAATGAAACTTTAGCTACTACTAGTACTAATAATAATAACCAAAATCTT
CCAGAACCAGAAATGCCCTTTCCTCTACATAACCATCACTTCATGAGTGGCACTAGCAGCTTTGTTCAT
CATGGTAATTTCATGCTTGATCAAAATCAAGCCCCAATTATTGATTTCATGGAAAGTAAGTACGAGGCTT
TAGTTGGGAGTAGTTCAAGAAACCAACATTTATTTCTTGGGAATGGTGATAATAATATTGGAATGATGA
GTAATGCTGGATTTGGTCATGATAATATTATTGCACCAAATTTTCCATTTGGAATGGCGTCGATGAATATG
GATAATGTGAACAACTTTGGAATGTTGTTACCATACGAAAATAATCACCATCATCATGAGGAACTACAA
AGTATGAATAATGCAGTTGATGTGAAGCCAAATCCCAAGATTTTGTCACTGGAGTGGCATGACCAAGC
TGGCAATAAAGAGTCATCATTTGGATATAATTATTCTGGTAGTAGTACTGGAGGCCTAGGATCTTGGACT
GGCTTGATGAATGGTTGTTATGGATCATCAACAACAAACCCTTTAGTCTGA
>CaDof16
ATGGATACTTCTCAGTGGCCACAGGGTATAGGAGTAGTGAAAGGTGTGGAACCCTCATCAAAGGCAGT
AGTACTACCAGATCAAAGAAAGCCAAGGCCACAAAAGGAACAAGCAATAAATTGTCCAAGGTGCAAT
TCAACAAACACAAAATTCTGTTACTACAACAATTATAGTCTCTCTCAGCCAAGGTATTTTTGCAAGACT
TGTAGAAGGTACTGGACTGAAGGAGGTTCTTTGAGGAATGTTCCTGTTGGTGGTGGTTCTAGAAAAAA
CAAAAGATCTAACAATAACAGCAACAACAACAATAATTCTTCGTCATCTAACAGTTCATCCACGTCATC
ATCATTGTCATCGTCCAAAAAACTTCTTTCAGATCTAGCAAACCCTAACGACCTCAATTTAACTTATAAC
CCAATATCTGCAACTGCTGCTGTTGCTACTACTAGTACTGCTGGCAATTTCAGCAATTTTTCGGAATTTA
TGGCTTTACCATTGATCCACCCAGCTAATTCGACATCTTCATTTATGCCAAACAATTTGTACACATCATC
AACTGGGCTTCCTAATTTGCATGATTTGAAGTCCAGTAGTCTTAATTTTTCTTTGGATGGATTTGAAAAT
GGTTATGGAAGTTTGCAAGGAGGTGATCAAGAGGCAAAATTGTTTTTCCCTATGGATGATTTGAAGATT
AATGTTTCAACGGCGAATGATCAGTTTGAAGAAAATAGAGAGCAAGCTGCAGCTGATCAATCTAATGG
ATTTTGGAATGGAATGTTGGGTGGAGGAGGATCATCCTGGTAA
>CaDof17
ATGTCTTCAGAAATCGGCGACAGACGTCCGGCGAGACTACCGGCGCCGGTGAACGGAACACGACCAT
CGGAACCGGAGAACTTGCCATGTCCACGTTGTGATTCTACTAACACGAAGTTTTGTTATTACAACAACT
ATAACCTATCTCAACCTCGTCATTTTTGCAAGTCTTGCCGTCGTTATTGGACACGTGGCGGCACCTTAA
GGAATGTCCCCGTTGGTGGAGGTACACGTAAGAACTCCTCCCATAAACGCCCACGCACCACCACCGG

AGCCGCCGTTCAAGAACACACCAACCCGGGTTTGGGTTCCGGGTCGGGTTCGGTTTCGTTAATGGGTT
GTGAAGTGAACTTGAATGAATCAGTTCAGGAAGGTGGCGGAAATGGTACGGCGTCGTTTACTTCGTTG
CTGACAGCTGGTCCAGTGGGAGGTGGGTTTGGGCCATTAGGTGGATTTGGGCTTGGGCTAAGTGGGTT
TGGGCTTGGAAATCTTGATTGGCCCATGGAGCAAGTAGTAGTAGGCGGCGTCGGAGGTAATGGCGGCG
ATGGTGGTGAAAATGACAAGTGGCAGCTGAGTGGCGGGGAGGTTGAAGGCGGCGGAGGTGGTGGCG
ATGATGATTGTTTTGGTTGGCCTGATCTTGCTATTTCAGCACCAGGGACGAGTCTTAAATGA
>CaDof18
ATGGAGAGAACACGAAAGTCCAACATTGAGCAAGCACCAAATTGTCCTAGGTGTGCCTCTACCAACA
CAAAATTTTGTTACTACAACAACTATAGCTTGTCACAACCTCGGTATTTTTGCAAAGCTTGCCGAAGAT
ACTGGACTAAGGGTGGTTCTCTTAGAAATGTCCCCGTTGGTGGCGGTTGCCGGAAAAGCCGACGTTCA
AGATCTTTACGAAAAGACGATAATACCCTTCAATCCCCTAGCCCCGCCTTCGAAACTCCCGGAGCTAAC
ATTGATCTAGCCGATGTTTTTGCGAAGTACTTGAACCAAGGTACAGCCAATGATCATGATGATGATGAT
CAAGATAATAATATTATTCTTCAAGAATCTCAAGATTATTCTTCTATTGGAGCAAGCTTATCGGAATCTCC
TTCATCAGATAGCTTGGTTAATAATCCCACTTCTTTTGAGAATGAGAGTCTATTTGATGAGACCATTATG
GCCAGTTTTCAAGATTATCCTTGTGGTAATTTCCTTCAAGAGGAGCAAGGGGGTCCAATTGATCAAGTA
GGTAACCAAGATTTTCTTGATTTTAACACAAGCTTTCTTGAAATGCAAGCCATGCTTGGGGATGAAATA
ATAGGGCAAGGGGAGGAATTTGATCATTATAACACAAGTAATTTTTCTTGGCAATCAATGATGCAGTTT
CAAGATTTTGGATCAATTTTAGAACTAGATGATCAGCTCAAGAATTCAACATCAAACTTAGCCAGTGAC
AATAACAACTATAGCTCATTTGATCTATCTAACTAA
>CaDof19
ATGTCTGAAATAAGAGATCCTGCTATTAAGTTGTTTGGTAAAACAATTGGTATGACACAACAAGAAACC
AATTGTGTTCATGATCATCATACAAGTGCTTCATTTGACAATGATAAGATCGCTTTAGGAGGAGAACTTA
CACAAAGCAAACAAGATGATGTACTTGTCGATCCAACTGCCGACTCTTCGGTTGAACCAGAAACATCA
TCTGGTATAAGCGATGACCTCAAGATGCAGGATGCAGAAAAAGAAATATTATCGTCTAAATCTATCGAG
GAGGAAGATTCAAGTGAGGAAAAAACACTCAAGAAGCCTGACAAAATAATTCCATGTCCCCGATGTA
ATAGCATGGAAACAAAGTTCTGCTATTATAACAATTACAACGTCAATCAGCCCCGTTATTTCTGCAAGA
ACTGCCAGAGATATTGGACCGCTGGAGGAACAATGAGAAATGTGCCTGTGGGTTCTGGCCGCCGCAA
GAACAAAAATTCATCCACTTCGATTTATCCTCTTCAAGCAGGTCGGGTTGAAGCAGCACACGGAATGC
ACCTTCCTGCTTTAAGGACAAATGGTACTATCCTTACATTTGGATCGGATAAACCACTTTGTGATTCCAT
GGCTTCTGCATTGAACATAGCTGAGAATTCACATAATATGAATCGAAACGAATACTGTGGATCCGAACG
AAGAATGCCTGCAATTGCGAATGATCAATCAAATGGAACTTGTAGCACAGCCTCAAGTATAACTGACA
AAGAAAGCAATTCCGGTCCACATGATTTAGCTAATTGGAGTAATTTCCAGCCATTTTCTCCTCAAGTAC
CTTACTTTCATGGCGCTCCGTGGCCTTATTCTGGCTTTCCAGTATCATTCTATCCAGCAACACCCTACTG
GGGCTGCACCGTAGCAAGCCCTTGGAATGTACCTTGGCTTTCTTCTGATCAATCAGTCCACAACACAA
GTCCTGCTTCACCGACTTTAGGAAAACACTCTCGTGATGAAAGCAAGTTTGATCCATCACAATCACGG
AGAAGAGATGCTACTTTGCAGGACAGAGAAGGAGAGAGATGTGTACTGATTCCTAAGACATTAAGGAT
TCATGATCCAAATGAAGCGGCTAAAAGCTCTATATGGTCGACATTAGGTATTAAGAATGAGAAGATTCA
TTCGACTCACGGAACAATGCTCTTCAGTTCCTTCAATCCAAAAGCTGATCTCAGAAATCATGAACGTG
ATGCTTCTTTACTCTTACAAGCTAATCCAGCAGCTTTGTCTAGATCACTTAAATTCCGCGAGAGCACCC
AATAA
>CaDof20
ATGGCTTTCTCATCTATTCCACTCTATCTAGATCCTTCCAATTGGCAACATGAGCAAGAAAATCAACAA
CAACAACAACTTGGAGTTACTAATCATGAGATGAATTACCCCTCTGAGCTCTCGCCAGCTGTGTTGCCA
CCTCCAGCAGCAACAGGAAGTGGCGGTCCAGCTGGCTCAGTTAGGCCTGGCTCAATGACAGAAAGAG

CCAGGCTAGCAAAGATACCACAGCCGGAGAATGCCTTGAAATGTCCACGTTGTGCATCCACGAACAC
AAAGTTTTGCTACTACAACAACTACAACCTTTCTCAACCGCGCCACTTCTGCAAGACTTGCCGGAGGT
ACTGGACTAGAGGTGGCGCGCTAAGGAATGTCCCGGTTGGAGGTGGTTGTAGGAGGAACAACAAGA
GAAGTAGCAAAAGAAGTAGATCAACAAAATCACCAAATAGAAGTGATCAAAGATCAAGAAATAATGT
TCCAACTATTAGTACTAGTACTATTACTTTCCCTAGCCATTTGCCTCTTCCTAATACTAGTACACATTTATC
ATTCTTGAATACACCTTTTCATAATTTAAATGACTTCAATTCTACACAAAATGACATGAATTTTGGAGAA
ATTCAATCTCATGAGGGTGATGGAAGATTTATTGACCAATTTAGACTTCAACAAATGCAACAATTTTCTT
TCTTCCCCCCCTTAGAACAACAACCTAGTAATTTATATCCAATTAGTGAATTTGGGATTAGTCATGATTT
GGAAAATGTGAAGGTGGAAGAGAATAAGAGTAGTATTAATAGCCAAGGGATGAATTTACAAAGAAATA
ACACTTTGGGGGTTAATCAATTTTGGATAGATTATAATATTTCTAGTACTTCTACTAGTCAACTATTATGA

>CaDof21

ATGTCAGAAGTTAGAGATCCAGGGATTAAACTTTTTGGAAAGACAATTATTTTGCCAATTGATGATCTA
CGAAGCTCTTCTATTAACACAACTTCACATGATGATCAGATCACTTCTGAAGGAGAGCTTACACAGAGT
AAAAGAGATGATTTTACCAATTCCACTGCAGACGAGTCGGTTGAACCAGAAATATCATCTGGTATAAGT
GATGACCCGAAGGCACAGGATGCCTATAAAATAACATTGTCCCCAAAATCTACCGAGAAGGACGATCC
AAATGAGGCAAGTGGCACTCAGGATAAGGTACTGAAAAAGCCAGACAAAATACTTCCCTGTCCGCGC
TGTAATAGCATGGAAACCAAATTCTGCTATTATAACAATTACAATGTCAACCAGCCCCGTTATTTTTGCA
AAAAGTGTCAGAGATATTGGACAGCTGGGGGAACAATGAGAAATGTGCCTGTGGGTTCTGGTCGCCG
CAAGAACAAAAGTTCTTCCACTTCAAGTTACCGTCATATAATGGTGTCAGATGCCCTCCAAGCAGCCC
GGTTTGAAGCAGCAAATGGGATGAACCTTCCTAGTTACAGAACAAATGGAACTGTCCTTGCATTTGGA
TCAGATAAACCTCTTTGTGATTCCATGGCTTCTATATTGAACATTGCTGAGAAATCACATAACTCTATTC
AAAATGGATTTAATGGATCTGAACAAAGAATGATTGCTTCTTGTGGAGGGAAAGAAATTGGGAATGAT
CGATCAAGCGAGGCTTGTAGCACAACCTCAAATTCAACAGAAAAGGGAAATGATTCCACTGCACGTG
ATTTAGCCTGGAAGAATTTTCAGGCCTTTCCACCGCAAGTGCATCACTTTCCAGGGCCTCCTTGGCCTT
ATACATGTAATGCGGCTCCCTGGACATCTGCAGTGCCACCACCCACCCTTGCTCCATCTGGTTTTCCGG
TGTCATTTTATCCTCCACCCCCATACTGGAGTTGCACTATGGCAAGCCCTTGGAATGTCCCGTGGGTATC
TCCACCACCCTCTTCTGCTAGTTGCTCTGTCCATGGCAACAATCCTAATTCTCCAACCTTAGGGAAACA
TTCTCGGGATGAAAGCTCGTTTAATCCATCAAACATGGCGAAAGAGGATACTTTGCAGGATAAAGATG
GAGAGAGATGTGTACTGATTCCTAAGACACTGAGAATTGATGATCTAGATGAAGCAGCTAAGAGCTCT
ATGTGGTCTACACTAGGCATTAAGAATGATAAGAATGATTCAGCTAATGGCACGAGGCTCTTCAAGGCC
TTCAATACAAAAGTTGATGAGAGAAACAATGAATCCGACACTAATCTAGTCCTGCAAGCTAACCCCGC
AGCCTTGTCCCGATCACATAATTTCCAAGAGAGCACGTAA

>CaDof22

ATGGATCCCTCTAGTGCACAACATCATCATCAGGAATTGTCTTCTCAAACCCTAGAAAGCATGTTGGTG
AGCACAAAGCCACAACAAGATCAAAAGAAGCCAAAGCCACCAGAGCAAGCAATAAAATGTCCAAGA
TGTGACTCCTCCAACACAAAATTTTGCTACTACAACAACTACAGTCTCTCTCAACCTAGATACTTTTGC
AAATCATGTAGAAGGTATTGGACTAAAGGAGGAACATTAAGAAATGTTCCAGTAGGAGGAGGCTGTAG
GAAGAACAAAAGGTCTTCATCATCATCAAGAAGTAGTAGCCAAGAACAACATTCCATCAACATTCCCA
ATTGCCCTACTAATCCATTTTCTTATGATTCTAGTGATTTGAGTCTAGCTTTTGCTAGGCTTCAAAGACA
AGAAAGTGGGCCATTGGGATTTGAAAACCATAGTAATATTTCAATGATCATGTGCAATGAAAATCCAAG
TGGATTTCTTGATGCACTTAAGGGTAGTACTGGATTTCTTGAAAATAATAATCCAAATGGATTTCATCAC
CAGAATCTGTTCTATGGAGTTGGAAATATTATTAATGGGGACATGGGATTACATAATGTGGAAAATGGTG
GAATGGGGGTGATCAATAATAATGTAAGTGATCAAGAAATGGGATTAATGCATAATTATGATCAAGAAA
TTAGCAGTGGTACTGTAACAACTACAACAGCTACAACAATGACAACAGTTAAACAAGAGATGTGCAA

CATGGCTAGAGATCAAGGTGACAACAAAGTTTTGTGGGGATTTCCATGGCAAATTAATGGAGAAGGAC
ATAATATGTCTGATTTTGATTCAACCAGGAGAATGTGGAATGGAGTTGGTGGCTCTTCTTGGCATGGAC
TTCTCAATAGTCCTCTCATGTAG
>CaDof23
ATGGTTTTCTCATCTTTTCCTGTATATCTAGATCATCCCAATTTGCATCAGTTACAACAGCCAGATGGCCA
TCAACAAGTTGGAAACCCTGGGCTGGAGAATCCCCAACTTACAGCTCTGCAGCCCCCACCTGTTCAGA
TGGGGGCCAGTCCTGGCTCGATCAGACCAGGTTCTATGGTGGATCGAGCCAGGCTAGCTAAAATTCCA
CTACCGGAGGCTGGACTAAAGTGTCCAAGGTGTGATTCAACAAATACAAAGTTCTGCTACTTCAATAA
CTACAACCTTTCACAACCAAGACACTTCTGTAAGACTTGTCGCCGTTACTGGACAAGAGGGGGAGCC
TTGAGAAGCGTGCCGGTAGGAGGAGGATGCCGGAGGAACAAGAGAAGCAAAAGCAGTACAAATAAC
AACAGCTCAAAGACAACTGGAAGTAATGTCAATAGTACTACTACTGCTGATCCGAGACAAATAGGTAC
TTCAACAAGTGCAAGTCCATCTAGCTGCAACACAGAAATAATTACTGGACGCCATCACTTTCCACACG
AGCAATCCCCAGTACAGTTTACTCCACTCATGGCCGCCTTCCAAAACCTAAATCATCACTATGGCGGAT
TTCAGCCTCCTCCTTTGGTCTCAACACAGGGCGCTGCTACTCTTGGTCATCCTGAAATGGGATTTCAAA
TAGGTAGTACTACAAATAGTACTAACAATTTGTCTGCTCCTTCAGGAGTATCTGATCATCAGTGGAGATT
ACCTTCTTTGGCAGCAAACACAAATTTGTACCCTTTTCATCAAGGTGAAGGAATCGAATCATCATCTGG
TAATAGTATTGCCCATGATGATCAAGGGTTAAATTCGACGAAACAGTTTTTGGGGACAATGGAAAATAA
CACTAATCAATATTGGGGTGGAAACGCATGGACAGGGTTTGCTGGACTCAATTCCTCTTCTTCAGCCAG
CCATCTCCTTTGA
>CaDof24
ATGGGTATTACTTCTTTGCAAGTGTGCATGGATTCATCAAACTGGCTACAGGACACAATTAACGAGGAA
ACAGAATTCGATTCTTCTTCTTCCCCATCAGGTGGTGACATATTCACATGTGCAAGGCCATTAATAGAA
AGAAAACTAAGACCCCAACATGACCAACCACTCAAATGCCCTCGTTGTGACTCAACACATACAAAATT
CTGTTACTACAACAATTACAGCCTTTCTCAACCAAGGTATTTCTGTAAATCTTGCAGAAGGTACTGGAC
TAAAGGGGGAACTCTAAGGAATATACCTGTTGGTGGTGGATGTAGGAAGAACAAGAAAGTCTCTTCA
AAGAAATTGTCTAATGACAACAACATTACCCCTCATAATGTTGTAGTATCATCTAATTCATCTCCTATTTC
TAACTACCCTGATATGGCATTATCCCACTTTGGTAATTTTATGGGAATTAATACTAGTAATAATAATAATAA
TATTAATCATAATTTCATGCTTGAAAATCATCATCATCACGTACCTATTGATTTTATGGAGAGCAAGTATG
AAGCTTTGGTGGGGACTATTACTAGTTCAAGAAATCAAGATTTTCTTGGGAATGTCGATGTTACTGCCG
GCATGATCAACGGTTATGGTGAGATGGATAATTCCGGAATTGTTGGACCAAATTTTCATGGTGGGTTTT
GCTCTACATTTGGGTTGCCTATGGATGGGAACCTAAACTATGAGGGACAAAATATAACAATGGATGTGA
AGCCAAATCCCAAGATTTTGTCACTTGAATGGCTTGATCAAGGCTGCTCTAATGCTGGTAATATTAAAG
AATCTTTTGGCTACTTGAATGGAGGACTAGGATCTTGGACTGGATTGATGAACAATGGTTATGGATCAT
CTGCGACGCACCCGTTAGTTTGA
>CaDof25
ATGGAGACTACTCAATGGTCGACGCAGGTGGAAATCGGAGCTGTGAAATCATCGATGGGTGCCGAAAT
AGGTTCAAGGCCAGGTGGAGAAATAATTAATAAGAAGGCGAGGCCAGTGAAGGATGGAGCAATAAAT
TGTCCTAGGTGCAATTCAACAAATACAAAGTTTTGTTACTACAACAATTACAGCCTGACTCAACCAAGA
TATTTCTGCAAGACTTGTAGAAGGTATTGGACTGAAGGTGGAACTCTTAGGAATGTTCCTGTTGGTGGT
GGTTCTAGGAAAAACAAAAGATCTTCTTCTTCCCAAAAAGTTCTTGATCTGAACCCTAATTTGAATAGT
CATGTTCCAGATCATCAGCTTCATCAAAACCCTAATAAGATTAATATTGTTGGAAGTACTAGCCAAGATC
TTAGCCTCGGGTTTCGAACTGTGCCACATGATCATCAGACGTCGTTCCATGGTGTCCTCCCTCAGTTTC
TTGAATTGCCAAAGATGGATGCCAGCAACAATCATCTAGGTAGCACACAAATTTCAGCTCTGGAGCTG
CTTAGGACTGGAATTGCTTCAAGAGGGTTCACTTCATTCATCTCCTCACCATCAACACCAGATTTGAAT

GCTTTATACAGTACTTCAGGATTTCCATTTCAAGAATTAAAGCTTAGTGGTGGTAATACTCATGATCATA
CAGCTTCTTTGAGTAATTATCCAAGTGGAGGGCCAGGGGTTCAAGAAAATGGAGGTGCAAGAATAATG
TTCCCTCTAGGAGGATTGAAGCAACTTTCAAGTACAAATGAAGCTGATCATCACCATCATCAGACAAA
GGGACAGGAGAATAATAATTCAAATGCTGGATTATATTGGAATGGGATGTTAGGTGCTGGAGGATCCTG
GTAA

>CaDof26

ATGAATTTTTCTTCAATTCCATATCTTGATCCAGCCAACTGGCAACAGCAAGGTGGAGGTAGCATCCAA
AATCATCATCACCATCAACTTACATCACCACCATCTCAGACTGCACCACCGCCGCAAGTACCACCGCCT
CCTCCCGTACCCCTGCAGCCTCATGGAGGTGGCGGTGGTGCAGGTACCATTAGGCCGGGCTCGATGGC
TGATCGAGCCCGGATGGCTAACATACCTATGCCTGAGGCAGCCCTAAAATGCCCTAGATGTGATTCAAC
AAATACCAAGTTTTGTTACTTCAATAACTATAGTCTCTCACAGCCTAGACACTTTTGCAAGGCTTGTAA
AAGGTACTGGACTAGAGGCGGCGCGCTGAGAAATGTCCCCGTGGGAGGGGGTTGCAGGAGGAACAA
AAGAAGCAGCAACAGCAAAAGTGGTAACAACAACAATAATAATAATAATTCAAAATCTCCGGCTTCTA
GCACGAGTACTGATGGTCGTCAAGCTACTAACAATTCGGGTTCTACAAGTACAATTTCATCACATAGCA
ACAGCTTTACCGGTCCAACATCAGCAGCTAGTTTATTAGGTCTTATGACCCCTCAAATTCCGCCTCTTC
GTTTCATGTCACCTTTAGGTCAATTTAGTTCTGATCATCATCATCACCACCACCATTTTACTCCGAGTAAT
CACATGAACTTGAATTTTTCTACAAGTACTTGTGGTAACATATTAGGTGGTACTACTGAAGGTATGATGG
TTAATACTAACAACAATTTGCTCGGTGTTGGTGTTGGTGTTGGTGTTGGTGTTGGTGCTGGTGCTGGTG
CTGGTGTTGGTGGTGTTGCTTCGCTTTTATCAAGTGGAAACCTTGAACATTGGCGTATGCCACAACAAT
TCCCTAATTTCTTAGGTGGATTTGATCCGTCTAATTCGCCTTCTTCTGGTGTTAGTAACTACCCATTTCAA
GGTGGTGTTCATGAGGCAGTACAATTCCTTGGTGGTGAGAGTACGAGTCAAATTAGCAGGCCAAAAAT
CTCGACATCAATGCTGAATCAGATGGCTTCAGTGAAGATGGAAGATAGCAACAATAACAATAATAATCA
AGATCAATCAGCTTTGTCAAGGCAATTGTTGGGGATACAAGGAAACAATGAAAATTGGAATACTGCTA
GTGCTTGGAGTGATCTTTCTGCTAGTTTTAGCTCTTCTTCCACTAGTAATGCCTTATAA

>CaDof27

ATGGTTTTTTCCTTTATTTCTGCTTATCTTGATCCATCCAACTGGCAACAGCAAGTTGGATATGGCATCC
CAAATACCCAGCTTCCATCAGCACCACCACAACCTACGCCGCCGCATCCACTAGCGACTACTCCACCA
CCACCTCGGCCCCATGGTGTGGTTGGTGAAGATTCAATCCGACCGGGCTCCATGGCTGACCGAGCCCG
GTTAGCAAATATACCAATGTCAGAGGCAGTTCAAAAATGCCCTCGCTGTGAGTCAACAAACACAAAGT
TTTGCTACTTCAACAACTACAGCCTCTCCCAGCCTCGTCACTTCTGCAAGACCTGCAGAAGGTACTGG
ACGAGAGGAGGCGCTATAAGGAGCGTCCCAGTGGGTGGAGGTTGTCGCAGGAACAAAAGAAGCAGC
ACTAACAGTACTACTACTAGTGCTACTAAATCCAGCAACAATAACAATACTTCTAAATCACCAGCTTCT
AGCCAAGCTACTAATTCTGGTTCCACAAGCAACAATAGTTGTACTTTTTCAAGCCAATCGTCAGCAGCT
AGTTTATTAGGTCTAATGAACCCTCAAATTCATCCTCTCCGTTTCATGTCCCCTTTAGGCCAATTGACTG
ATCAACACTTCACCCAAAATGATAATGTTACGATGAATTACTCGTCATTTTCTTCTTCATCACCAGCTCC
AGTCATTGTGGAATCAACCATTGAAAGCACGAATTTTCAGCTCGGAATGAGTAACAATTTAGAGCAGT
GGCGGCTACATCAACAACTGGCGAGCCAATTCCCATACAATTTGTACGGGGGATTGGATTCGTCTTCTG
CTTCTGGCTCAGGGCTTTACCATTTCCACCCAACTCATTACTCAAGTAATGAGGTGGGTGGTGGTGGTG
GTGTCATAAGTCAAATTAGGTCAAAAGTTTCGAACCCTATGTTGACTCAGCTGGCATTGATGAAAATGG
AAGACAATCAAGATCATCTAGCAACTATGCCACGACAGTTTTTAGGGCACGAAAACTGGCCAAGTAAT
GGTAGTCATGCTAATTGGAATGAGCTTTCCGTGAGTTTTAGCTCCTCTTCCACTAGTAATGTACTATAA

>CaDof28

ATGTCTTCCCAAACACTTGAAAGCATGTTGGTTTGCACAAAGCCTGAGCAAGAAAAGAAGCCAAGGC
CAGCAGAACAACAACCCCAAAAATGTCCAAGATGTGACTCTGCCAACACCAAGTTTTGCTATTATAAC

AACTACAGTCTCTCTCAGCCCAGATACTTCTGCAAATCTTGCAGAAGGTACTGGACTAAAGGAGGTAC
ACTTCGAAATGTTCCAGTTGGTGGTGGTTGCAGAAAGAACAAAAGGCCATCGTCATCATCCAAGAGA
AGCCAAGATAACAGCCCTAATAGTAACAATCCAATATCTCCTCTCAGCATACCTACTATATCTTCCTATGA
TCATTCCTCCACTGATTTGAGCCTAGCATTTGCTAGACTCCAAAAACAAGCAAGTGGGCATCTGGGAA
TTGATCAAGAACACGACGACGACGACAACAACAACAACAATATGTCAATGCTGTACAATCCTGACAA
CACTAGTACTACTGTCCCTTCTAGCTTTCTTGATGCACTAAGGGGTGGATCATTTCTTGAAAATGCTCCA
AATGGATTTCACCACAAAATGTACTATGGGAACTATGGAGGTATGCTAGGGGCGAATAGCGAAGAAAT
GGGAATGAATTACGATCAAGAAATGAGCATTGGTACAACTAGTGCCAGTACTATAATGACAACAACAG
TGAAGCAAGAAATGTGCGGTATTGCAAGATCATCTGAAGGCGATCAGAATAATAATAGCAAAGTTTTG
TGGGGTTTTCCATGGCAACAGATGAATGAAGATCATTTAAACAAAATGAATATGAGTGATTTTGAATCA
ACAAACAGGCAAAATTGGAGTGCATTTGGAGTTTCTTCTAATTGGCATGGACTTATCAATAGTCCTCTT
ATGTAG
>CaDof29
ATGACGAGTCAAGATAACAAAGATGATAGCCAGAGCTCCGGCGGTGGCCCTGCCGGAGCAAGGCCAA
AAGAACCAGCTTTGAATTGTCCGAGATGCGATTCTCCCAACACAAAGTTTTGCTATTACAACAATTACA
GTCTTTCTCAACCTAGACATTTCTGCAAGACTTGCAGAAGGTACTGGACTAAAGGTGGAGCGTTACGC
AACGTTCCGATAGGTGGTGGTTGCCGGAAAAACAAGAAGATAAAGACTTGTTCATCATCTTCAAGGGA
TTCAAAAGATACAACTAGTGGTTCTTCAGATATTGGTGGGCTAAAATTCTTTCATGGTCTTTCACCAGCT
ATGGATTTTCAACTTGGAGGACTAAATTTTCCAAGATTGAACAATACTTCAACATCAACTGGTGGAAGT
ATTTTCAACCAATTTTCTACATTTGGTGACATTTCAACTACTACTACTAATTGTGCTACAAATATTGGATC
AAGTTCTTGTTTCAATCTTGATCCTTTAGGAAGTTGTTCTGGTTCTTTATTGGGATTCAATAATTTCCCTT
TCTCAAGTTCTATACTAAAACAAGGTACAACTGCAGTTCAAGAAATGGGATCAATGGGAGTTCATCAT
GGCACAATGGCATCTTCTATAGAGTCATTGAGTTCAATTAACCAAGATTTGCATTGGAAATTACAACAA
CAAAGATTGGCCATGCTATTTGGTGGTGAAAATCAAAAAGAGAATAACATTGTTTCATCATCAATCCCT
TTTGATGATCAAAATCAAAATCAAAACCAAAACCAAAACCAAATCCAAATCCAAAAGCCACAGCCAA
TTTTGTTCCAAAATCTGGAGATTTCATCGTCAAAACAACAAGAAGATCATCATCATCAAGAAGCAACA
ACATTTGGAAACGACAATATTAATTCAAGAAAAGATGTTAATATTACAATTGGAAGTCATGGGAATAAT
TTGGCAACTGAATGGTTCTTTGATGATTCATTTGGTGTTAATCCAAATTCAACAAATACCATCAATGAAA
ATGAGGATCAAAATGGCAACAATTGGAATAGCACTATTCAAGCTTGGAACAATTTGAATCAGTATAGTA
CACTCCCTTAA
>CaDof30
ATGGTTTTCTCTTCTTTTCCAGTCTATTTAGATCATCCTAATTTGCACCATTTACAACAAGAACCAGATCA
TCATCAGCAAGGAAGCCCTGGCCTTGACAATCCTCAACTTCCACCTGTCCAGCCACCCACTTTGGTGG
GTGGCGGGGCGGGCTCGATCAGGCCTGGTTCGATGGTCGATCGAGCCCGGATAGCCAGGCTTCCGCTG
CCGGAACCTGGACTGAAGTGTCCGAGGTGTGATTCCTCGAACACTAAGTTCTGCTACTACAACAATTA
CAACCTCTCGCAGCCGCGCCACTTCTGTAAGAATTGCCGCCGTTACTGGACGAAAGGAGGCGCCTTG
AGAAATGTGCCAGTCGGAGGCGGCTGCCGTAGGAACAAAAGAAATAAAAGCACCTCCTCATCAGTTA
GTTGGGGAAATGGGATTTCATGTAGGGAGTAA
>CaDof31
ATGCAAGATCCATCATCATTAATATATTCACAGATCAAGCCTCAATTCCCAGATCAAGAAGTATTGAAAT
GTCCTAGATGTGACTCAATCAACACAAAGTTTTGTTACTACAACAATTACAACCTTTCTCAGCCACGAC
ATTTTTGTAAGAATTGTAAAAGGTATTGGACTAAAGGAGGTATTTTAAGGAACATTCCAGTTGGTGGAA
GCTCTCGTAAGAACTCGAAGCGATCATCATCATCATCAAGTAGTAGTAAACAATCCTTAAGGACATCGT
CTTTATCACCATCTTCAGCACAGAATGGAAAAATAGAGGTTTTTCCTACACCTGCTGTTCCAGCTTTTG

ATCAGGAAAGCCCGATTCTTGATGCGAACGGGCCTTTTGGCCCATTCCTGGTGTCCAACGGGTCAGAG
ATAGGCAATTTGTTAGAAGGTTTGAATCCAAACGGGCCTAATTATTCGGGCTCTGATGATGCTGCGGCT
CAAAGTGAGAATAATAGTACCAGAGATGATGAATATTTGGATGTTCAGAAGGGTGAAGATTCTAATTGT
TGGAATGTTAATACTAATAGTTGGGCTGATCTTGCTATTTACACACCAGGTTCAAGATTCCAGTAA

>CaDof32

ATGACTTTGGAAGCGAGTGAAAAAAGAGTGACCAAACAACAAACCGCAGGTGGTGTGCTACCACCA
GCACAAGAACCCGATCAGCAACTTCCTTGCCCACGTTGTGACTCAACCAACACAAAGTTTTGCTACTA
CAACAACTACAACTTGTCTCAGCCCCGTCACTTCTGCAAGTCTTGCCGCCGTTACTGGACCCATGGTG
GCACCTTACGTGACATCCCTATTGGTGGCGGTAGCCGCAAGAATGCCAAACGCTGTCGCATATATACCA
GTACCCCCTTTTCCTCCTCCACTGCCGTTTCTCCTCACGGTAATTCCCCGTTTTTGGTTCCTCTTCCTAC
AGCTAATCAATTGCTTTATGGTAGCGATGTGAAGCCGTGTCTCAATATGAGTGGCAATAATTTCACTTCT
TTGTTGAGTTCTCATGGACCAACAGGGGTTTTAGCACTTGGTGGGATTGAGGATGTGAGCTTTAGTATT
GGAAGAGCTGCTGTTTGGCCATTTCCTGGAGCTCCTGATTCGTTTATTCGTAATTATGACAGTGGTGT C
GGAGCTAGCATGTGGCAGTTTTCAGGCTGA

>CaDof33

ATGCAAGATATACATCCGATCGGAGGTGGAGGAGGCCGGTTTTATGGCGGGGGTGGTGATAGGAGGCT
AAGGCCAAACAATCATCAAAACCATCAAGCTTTGAAGTGTCCTCGTTGCGGTTCTCTCAATACAAAAT
TCTGCTACTATAACAATTACAATCTTTCTCAGCCGCGCCATTTCTGTAAGAGTTGCCGGAGGTACTGGA
CTAAAGGTGGTGTCCTGCGTAACGTACCTGTTGGTGGTGGATGCCGGAAGAGCAAGCGTGCCAAGCT
CAAATCTAGTTCTATCAACGCTGTTGCTGAAGTTGCAGAGGCTCAGGAGGAGCAGAAGTCTGATACGA
ATTCCAGCAGTGAGAGTTCTAGCCTTACTGCTACTACAACGGCGGCCACTGCAGCAGCTGCGGTAGCA
GAAGTGACGGCCACGGGGAATACTTCCGGTGGTGCAACGACGGAGGATGTGTCTGCAACTTCTTCCA
ACTCGGCTTCAACTTATCTCAACTTTCCGGATTCATCGAACTTCTTCATACCTCACAGTACTACTGATAA
CCACCATACCTTCGATGATCAGCCATTGACGGAGAACTTCTCGAGCATGATGACGTCATCAAACGATCC
ATCTATGGTAGGGTTTAACATTGCTGAAATTCCTGCGTACCGGTTGCCGGAGAATCAATCCTCGATGAT
CGAAACTTTGCCGTCCGGTGATCTGAAGATGGAACAGACGGGTACGGGTTACTTGAATCAAACGGATC
GGGTTGAGTTCCCCGGGTTACAGCAGAACAGGATAGACAACAGTGAACTCGCTTCACTAGATTGGCA
AACTGGTGGTGGTGGCGATCATGGTCTGTATGATTTAACAGGGACCGTTGATCAATCTTACTGGAATCA
AACGCAGTGGGGTGAAAATGATAACTCCCTTAATTTTCTCCCTTAA

The amino acid sequences are as follows:

>CaDOF1
MDTANWPQEIVVKPMEEIIGSSKPNNCVERKLVRPQKDQVVNCPRCNSTNTKFCYYNNYSLSQPRYFCKT
CRRYWTEGGSLRNIPVGGGSRKNKKSSSSNNNNNNNNNSNINHVVINNPLMKKLPDLIVPPLIQHDIEEYPE
RHFGNRPDNSTVLPQHHHDQNPRKIIHEGSQDLNLGFSSDFKTITELIQVANYDGGNKDNNNSTNNISTLPP
PSEPSSPAFSQLPSLNFSLDHHGLGNNNVRSGYGFFFPFVGLKQVSNASDHVRDQSTNNGYWNGMLGGG
GGGSW
>CaDOF2
MTCDSEIKLFGKILPVVVSVECDVAGGTSSGGDGDRCLEDGKASSADEGSENENQGADKDDLTGELNEA
KFEEGDQSQMMEESENPRTLSESENSSKSPTDEDSQAVKTSGTENEPTNVTNSEQNSLKKPDKILPCPRCN
SSDTKFCYYNNNNVNQPRHFCRSCQRYWTAGGTMRNLPVGAGRRKNKNLAAHYRHISISEGLLAAGVES
PNGLIHHPMFKPNGTILSFGTDLPLCESMASPLSQAEKRLSNGIQNGFHKAELKNSSCKVGDTGDECYKGS
NIPTPNVMVEEGKRELHKAVMHNINGIPSPFPCLHGVPWPFTWNAAVPMPAICPIPFPMPWLGPALQAASE
KTSGSDPTSPLGKHSREGDLLKPSNPRGKEQSEQKYSERSILVPKTLRIDDPDEAAKSSIWSTLGIKYDSAN
RGEFFKALQPKSDDKHNKANTPPVLHANPAALSRSITFQQSA
>CaDOF3
MERGTIWKPNVELAPACPRCGSTNTKFCYYNNYSLTQPRYFCKGCRRYWTKGGSLRNVPIGGGCRKSRR
GKSSSNTIHIHHHHELISRNLGHGVCLNPTNIDHHNQSTSSSLDHHHGPSIDLALVYSNFLNSTNSKSSQPED
RQNPELPDDLLLPDQGVLVTPSFELSSMIDMEFVNSELGQESRLLGAGAGDGVDFYFSGIHEEKQNGMNH
SDVHDDHYTNNMNVNANNSINHDHQLGNNNYMDLPPLPCEEIMWSNSHDHHHHMVFPNDLLRTSHNLT
TGGSEPEPEPESAVQNPSHDHSANNANDGSLFNLSNFGNIFRP
>CaDOF4
MERGTIWKSNVELAPACPRCGSTNTKFCYYNNYSLTQPRYFCKGCRRYWTKGGSLRNVPIGGGCRKSRR
GKSSSNIIHNHHHHELISRNLGHGVCLNPTNIDHHNQSTSSSLDHHHGPSIDLALVYSNFLNSTNSKSSQPE
DRQNPELPDDLLLPDQGVLVTPSFELSSMIDMEFVNSELGQESRLLGAGAGDGVDFYFSGIHEEKQNVMN
HSDVHHDHYTNINVNANNSINRDHQLGNNNYMELPPLPSEDIMWSNSHDHQYHHMVFPNDLLSTSHNLT
TGVSSEAEPEPEPESAIQNPSHDHSANNANDGSLFNLSNFGNIFKP
>CaDOF5
MSELKDPAIKLFGRTIQLPDVPDSSETMLEDSLPEEANGEEEDVEDQKDNIGGNLDDEEDEMEILTGKELQ
DQNSHPTKTDSIKVQPVGSDCSTRPSKSEEEQGEASNSQDKILKKPDKILPCPRCNSMETKFCYFNNYNVN
QPRHFCKNCQRYWTAGGTMRNVPVGAGRRKNKNSIPHYRQISVSETLPSAQADYPNGIQQPVLAFGSPTP
LCESMASVLNIADKTMHNCSQNGFHKPQDPGVPVSYGAGDNGDDHSRRSSVTTANSEDEVSKIVPDQLK
NCHNFPPYVACYPGAPWPYPCNSVPWSSAVPPPGYCPPGFPMPFYPAASYWGYTVAGSWNVPWISPTTGS
LIQTPPTSGPNSPTLGKHSRDENILKQLSNKEESSKENNPEKCLWVPKTLRIDDPREAAKSSIWATLGIKHDS
VESVGGSPFNAFQPKNDDNISVSENSTVLQANPAALSRSVNFNESL
>CaDOF6
MAEVQESRISQGIKLFGATIQVQEIKQAKVHQPTNKVDQDHDNNNNDQEKRPDKIIPCPRCKSMETKFCY
FNNYNVNQPRHFCKGCQRYWTAGGALRNVPVGAGRRKAKPPCGPGPHGDMNGLSDGCFFDVTNHHGN
NNNIHQLEFDGVVAEEDQWHLFQAAKRRRSTSHTQSC
>CaDOF7
MALIPSSTTNEIWPQIDEKNNLMMASNGSSSSNTRDMEKPIPDPSQQPPPPPHLKCPRCDSSNTKFCYYNN
YSLSQPRHFCKACKRYWTRGGTLRNVPVGGGCRKNKRIKRPSTNSSSSSSCTAHDIITTSTPNISTLNPSHH
VAHNSIDISSTNSINPLFYGLTSERSDLNIPFARLFNSRVSSHATVGEGQVYSLTDSIPGLMDRRMGLGFSNSS

VGGVNIMGENNNYGHGGFNPIKQIQDVVMTSNCTTSSTSLLSTYPNMFGSSTSTSTMASLIASSLQQQKF
MSNINGNNFHNLAPNYEELQMSRGDNNNNNSNVHEGGGNGITMLKAEKMDLSNHQIHEQIINSSDPSLS
WNGAWLDPSNMGSNSVPSLI
>CaDOF8
MEQGGRSSGESDRNQQQRRMKMPENNSSQPQPPPQKCPRCDSNNTKFCYYNNYSLTQPRYFCKTCRRY
WTQGGTLRNVPVGGGCRKGKRTMKGGSVGVSCGSSSSASESSSRSYQQQSQQIPNLSAAAAAVFFSGNN
NSRSQPPPLPSLSSLYTGGVGGGGGFLSTLASMQSMTQLSQGVNNDHSQLGVISASNSSQFGNFNIPSSIPP
KVQINQQMESGIYQMVVNREKPMESSFYPSDQISQFQPTRPLGSWTQRFINNNNNNNIWPNASASSSSSGG
ANSSTTAAGASLNPNQWPDLPGFGPSP
>CaDOF9
MGLSTKLVSIDDDGLDDWTCSSQNSLPEPPLIRRQPPSKPEPLKCPRCDSINTKFCYYNNYNKSQPRHYCK
GCKRHWTEGGTLRNVPVGGGRKNKRMRMTTDLVDHITGRKRVTLEEMNDQRCPLISTTITNTTTSSMPS
TIISNMDEDIKNIPSLASSSLPYDIFSSLKLSSIPQDGNTHFSLIPNSSTTQLSSNVYCNYDYMGKFDSTMEES
TITTVMPITSSSDLFSQPWKVPETSNDFIIENMSSNYWNWNEFDTLSTAADLNIQWDDLEIKP
>CaDOF10
MIQELFAGNTTLIGGDNNISKLSNITPSSSPLSCTTSNSSIAPAAAAGATATANASSPSNVESLRCPRCDSPNT
KFCYYNNYNLTQPRHFCKTCRRYWTKGGALRNVPIGGGCRKNKTITTAKSSAAKLKNSIPFEFIGKSGIFG
GFEQEIIPSNNNPFLFSTPHQNHNPILSLLRGNHHNLNLVKDEQKSIEVNLHNQFPSNSLSSLWKNNDTIVG
EVQNSTGFQELYQRLKASTSRCYPDIHGPSSSSSSMILESAPVAGGELGFWSPSFSTWTDLPTANSAYL
>CaDOF11
MPSDVNERRVTKQQQGGAPAPEPEHLPCPRCDSINTKFCYYNNYNFSQPRHFCKACRRYWTHGGTLRDIP
IGGGSRKNAKRSRTITTNSSLSSTLSPRDYQHASNPSAFLVPLTADHGGSLPFDVKPNVNMCGSFTSLLSSA
QGPGGLLALGGFGLGVGVGSGIEDMGFGLGRPIWPFPGVSHSNVENNSANGTGASMLGSTWQLASGGEG
GFVGATAGEIFNFPDLAISTHGNRMK
>CaDOF12
MSEAIASRDPAIKLFGRTIHLPLFPAPAPENTGYYCSSAGENEQKHEDQNPIQQKCDITKELPDYYDCSTAK
TSKSEEEQDETSNSQERNLKKTDKILPCPRCNSMETKFCYFNNYNTSQPRHFCKNCQRYWTAGGTMRNV
PVGAGRRKHKNSVLHDSYSSVSEALSKARTNFPNETQQPPLTISGTILTFDTDKPLSESMVSALNVSDKTM
QNYSGNGFRKYKELGIQAGDKGDDLSDGSSVTVVSSKDSDNGLPDTLRQNCNSFSNHLPCFSGAPWPYI
WSSVPCRNVVPPPGIPVSLFPATTYSGCTILGSWNVPKMPPPTASQNQVPLTSGPNSPTFGKHSRDENVLNS
MGTEEEPRKESNPGRRIWFPKTSRIGDLGEAAKSSIWGTLGIKHEVVDSVGGGLLKAFLPRSDERNCVSET
STLLQVNPAAISRSLNFNESS
>CaDOF13
MQDPSIYSQIKPQFPEQEHLKCPRCDSPNTKFCYYNNYNLSQPRHYCKSCRRYWTKGGTLRNIPVGGGSR
KNTKRSSSASTSKKITSTTTTTPLTSSVSASSSANPKPEPFGIPAIPSFDVTTGPFSSLLASNEPQFGNLLEALN
PNNSNNNGSNIQLSEFSRNPISSSGLGLGSGSGSGQNHSNGGESNNCWNGGSNGWPDLAIYTPGSNFQ
>CaDOF14
MREVKDGEIKLFGKKIALPENGKMLPVIVSGEDSDVGKSVSGSEVVTGEESSTGSDRGDPCLVDKEGNTS
SESDGGSEYEKEDADKDQMTRELSEANLEEKYQSQIMEESENPKSPSENKSKTTTDDDSPTAKSSRTEGDQ
NDAAANSQQKPLKKPDKILPCPRCNSMDTKFCYYNNYNINQPRHFCKSCQRYWTAGGTMRNVPVGAGR
RKNKNSASHCRHIMISEALEAARIDPPNGFHHPAFKPNGTVLSFGPDSPLCDSMASVLNLAENKTPNGIRN
GFYRPEHKNPSGLGGENGDDCSSGSSVTTSNSMAEGVKNRAPEAVMQTINAFPSPVPCIPGVPWPFPFAAV
PFPAVSPSGYPMPFCPPPPYWNCSVPGPWSLPWLTAPSPTANQNGSGSAPNSPLGKHSRDGELLKPNNPEG
QKNSEGFVIVPKTLRIDDPDEAAKSSIWSTLGIKYDSVSRGGLFKALQPKSSEKDHPATTFPALQANPAAFS

RSLSFQERV
>CaDOF15
MDSSSSPSGGDHHINLTCSRPIIVDQRRLRPPHDHSIKCPRCDSTHTKFCYYNNYSLTQPRYFCKTCRRYWT
KGGTLRNIPVGGGCRKNKKVSSKKSNTNETLATTSTNNNNQNLPEPEMPFPLHNHHFMSGTSSFVHHGNF
MLDQNQAPIIDFMESKYEALVGSSSRNQHLFLGNGDNNIGMMSNAGFGHDNIIAPNFPFGMASMNMDNV
NNFGMLLPYENNHHHHEELQSMNNAVDVKPNPKILSLEWHDQAGNKESSFGYNYSGSSTGGLGSWTGL
MNGCYGSSTTNPLV
>CaDOF16
MDTSQWPQGIGVVKGVEPSSKAVVLPDQRKPRPQKEQAINCPRCNSTNTKFCYYNNYSLSQPRYFCKTCR
RYWTEGGSLRNVPVGGGSRKNKRSNNNSNNNNNNSSSSNSSSTSSSLSSSKKLLSDLANPNDLNLTYNPISA
TAAVATTSTAGNFSNFSEFMALPLIHPANSTSSFMPNNLYTSSTGLPNLHDLKSSSLNFSLDGFENGYGSLQG
GDQEAKLFFPMDDLKINVSTANDQFEENREQAAADQSNGFWNGMLGGGGSSW
>CaDOF17
MSSEIGDRRPARLPAPVNGTRPSEPENLPCPRCDSTNTKFCYYNNYNLSQPRHFCKSCRRYWTRGGTLRN
VPVGGGTRKNSSHKRPRTTTGAAVQEHTNPGLGSGSGSVSLMGCEVNLNESVQEGGGNGTASFTSLLTAG
PVGGGFGPLGGFGLGLSGFGLGNLDWPMEQVVVGGVGGNGGDGGENDKWQLSGGEVEGGGGGGDDD
CFGWPDLAISAPGTSLK
>CaDOF18
MERTRKSNIEQAPNCPRCASTNTKFCYYNNYSLSQPRYFCKACRRYWTKGGSLRNVPVGGGCRKSRRSR
SLRKDDNTLQSPSPAFETPGANIDLADVFAKYLNQGTANDHDDDDQDNNIILQESQDYSSIGASLSESPSSD
SLVNNPTSFENESLFDETIMASFQDYPCGNFLQEEQGGPIDQVGNQDFLDFNTSFLEMQAMLGDEIIGQGE
EFDHYNTSNFSWQSMMQFQDFGSILELDDQLKNSTSNLASDNNNYSSFDLSN
>CaDOF19
MSEIRDPAIKLFGKTIGMTQQETNCVHDHHTSASFDNDKIALGGELTQSKQDDVLVDPTADSSVEPETSSGI
SDDLKMQDAEKEILSSKSIEEEDSSEEKTLKKPDKIIPCPRCNSMETKFCYYNNYNVNQPRYFCKNCQRYW
TAGGTMRNVPVGSGRRKNKNSSTSIYPLQAGRVEAAHGMHLPALRTNGTILTFGSDKPLCDSMASALNIA
ENSHNMNRNEYCGSERRMPAIANDQSNGTCSTASSITDKESNSGPHDLANWSNFQPFSPQVPYFHGAPWP
YSGFPVSFYPATPYWGCTVASPWNVPWLSSDQSVHNTSPASPTLGKHSRDESKFDPSQSRRRDATLQDREG
ERCVLIPKTLRIHDPNEAAKSSIWSTLGIKNEKIHSTHGTMLFSSFNPKADLRNHERDASLLLQANPAALSR
SLKFRESTQ
>CaDOF20
MAFSSIPLYLDPSNWQHEQENQQQQQLGVTNHEMNYPSELSPAVLPPPAATGSGGPAGSVRPGSMTERAR
LAKIPQPENALKCPRCASTNTKFCYYNNYNLSQPRHFCKTCRRYWTRGGALRNVPVGGGCRRNNKRSSK
RSRSTKSPNRSDQRSRNNVPTISTSTITFPSHLPLPNTSTHLSFLNTPFHNLNDFNSTQNDMNFGEIQSHEGD
GRFIDQFRLQQMQQFSFFPPLEQQPSNLYPISEFGISHDLENVKVEENKSSINSQGMNLQRNNTLGVNQFWI
DYNISSTSTSQLL
>CaDOF21
MSEVRDPGIKLFGKTIILPIDDLRSSSINTTSHDDQITSEGELTQSKRDDFTNSTADESVEPEISSGISDDPKAQ
DAYKITLSPKSTEKDDPNEASGTQDKVLKKPDKILPCPRCNSMETKFCYYNNYNVNQPRYFCKKCQRYW
TAGGTMRNVPVGSGRRKNKSSSTSSYRHIMVSDALQAARFEAANGMNLPSYRTNGTVLAFGSDKPLCDS
MASILNIAEKSHNSIQNGFNGSEQRMIASCGGKEIGNDRSSEACSTTSNSTEKGNDSTARDLAWKNFQAFP
PQVHHFPGPPWPYTCNAAPWTSAVPPPTLAPSGFPVSFYPPPPYWSCTMASPWNVPWVSPPPSSASCSVH
GNNPNSPTLGKHSRDESSFNPSNMAKEDTLQDKDGERCVLIPKTLRIDDLDEAAKSSMWSTLGIKNDKND
SANGTRLFKAFNTKVDERNNESDTNLVLQANPAALSRSHNFQEST

>CaDOF22

MDPSSAQHHHQELSSQTLESMLVSTKPQQDQKKPKPPEQAIKCPRCDSSNTKFCYYNNYSLSQPRYFCKS
CRRYWTKGGTLRNVPVGGGCRKNKRSSSSSRSSSQEQHSINIPNCPTNPFSYDSSDLSLAFARLQRQESGPL
GFENHSNISMIMCNENPSGFLDALKGSTGFLENNNPNGFHHQNLFYGVGNIINGDMGLHNVENGGMGVI
NNNVSDQEMGLMHNYDQEISSGTVTTTTATTMTTVKQEMCNMARDQGDNKVLWGFPWQINGEGHNMS
DFDSTRRMWNGVGGSSWHGLLNSPLM

>CaDOF23

MVFSSFPVYLDHPNLHQLQQPDGHQQVGNPGLENPQLTALQPPPVQMGASPGSIRPGSMVDRARLAKIPL
PEAGLKCPRCDSTNTKFCYFNNYNLSQPRHFCKTCRRYWTRGGALRSVPVGGGCRRNKRSKSSTNNNSS
KTTGSNVNSTTTADPRQIGTSTSASPSSCNTEIITGRHHFPHEQSPVQFTPLMAAFQNLNHHYGGFQPPPLV
STQGAATLGHPEMGFQIGSTTNSTNNLSAPSGVSDHQWRLPSLAANTNLYPFHQGEGIESSSGNSIAHDDQ
GLNSTKQFLGTMENNTNQYWGGNAWTGFAGLNSSSSASHLL

>CaDOF24

MGITSLQVCMDSSNWLQDTINEETEFDSSSSPSGGDIFTCARPLIERKLRPQHDQPLKCPRCDSTHTKFCYY
NNYSLSQPRYFCKSCRRYWTKGGTLRNIPVGGGCRKNKKVSSKKLSNDNNITPHNVVVSSNSSPISNYPD
MALSHFGNFMGINTSNNNNNINHNFMLENHHHHVPIDFMESKYEALVGTITSSRNQDFLGNVDVTAGMI
NGYGEMDNSGIVGPNFHGGFCSTFGLPMDGNLNYEGQNITMDVKPNPKILSLEWLDQGCSNAGNIKESF
GYLNGGLGSWTGLMNNGYGSSATHPLV

>CaDOF25

METTQWSTQVEIGAVKSSMGAEIGSRPGGEIINKKARPVKDGAINCPRCNSTNTKFCYYNNYSLTQPRYFC
KTCRRYWTEGGTLRNVPVGGGSRKNKRSSSSQKVLDLNPNLNSHVPDHQLHQNPNKINIVGSTSQDLSLG
FRTVPHDHQTSFHGVLPQFLELPKMDASNNHLGSTQISALELLRTGIASRGFTSFISSPSTPDLNALYSTSGF
PFQELKLSGGNTHDHTASLSNYPSGGPGVQENGGARIMFPLGGLKQLSSTNEADHHHHQTKGQENNNSN
AGLYWNGMLGAGGSW

>CaDOF26

MNFSSIPYLDPANWQQQGGGSIQNHHHHQLTSPPSQTAPPPQVPPPPPVPLQPHGGGGGAGTIRPGSMADR
ARMANIPMPEAALKCPRCDSTNTKFCYFNNYSLSQPRHFCKACKRYWTRGGALRNVPVGGGCRRNKRS
SNSKSGNNNNNNNNNSKSPASSTSTDGRQATNNSGSTSTISSHSNSFTGPTSAASLLGLMTPQIPPLRFMSPL
GQFSSDHHHHHHHFTPSNHMNLNFSTSTCGNILGGTTEGMMVNTNNNLLGVGVGVGVGVGAGAGAGV
GGVASLLSSGNLEHWRMPQQFPNFLGGFDPSNSPSSGVSNYPFQGGVHEAVQFLGGESTSQISRPKISTSM
LNQMASVKMEDSNNNNNNNQDQSALSRQLLGIQGNNENWNTASAWSDLSASFSSSSTSNAL

>CaDOF27

MVFSFISAYLDPSNWQQQVGYGIPNTQLPSAPPQPTPPHPLATTPPPPRPHGVVGEDSIRPGSMADRARLAN
IPMSEAVQKCPRCESTNTKFCYFNNYSLSQPRHFCKTCRRYWTRGGAIRSVPVGGGCRRNKRSSTNSTTTS
ATKSSNNNNNTSKSPASSQATNSGSTSNNSCTFSSQSSAASLLGLMNPQIHPLRFMSPLGQLTDQHFTQNDN
VTMNYSSFSSSSPAPVIVESTIESTNFQLGMSNNLEQWRLHQQLASQFPYNLYGGLDSSSASGSGLYHFHPT
HYSSNEVGGGGGVISQIRSKVSNPMLTQLALMKMEDNQDHLATMPRQFLGHENWPSNGSHANWNELSV
SFSSSSTSNVL

>CaDOF28

MSSQTLESMLVCTKPEQEKKPRPAEQQPQKCPRCDSANTKFCYYNNYSLSQPRYFCKSCRRYWTKGGTL
RNVPVGGGCRKNKRPSSSSKRSQDNSPNSNNPISPLSIPTISSYDHSSTDLSLAFARLQKQASGHLGIDQEH
DDDDNNNNNMSMLYNPDNTSTTVPSSFLDALRGGSFLENAPNGFHHKMYYGNYGGMLGANSEEMGMN
YDQEMSIGTTSASTIMTTTVKQEMCGIARSSEGDQNNNSKVLWGFPWQQMNEDHLNKMNMSDFESTNR
QNWSAFGVSSNWHGLINSPLM

>CaDOF29

MTSQDNKDDSQSSGGGPAGARPKEPALNCPRCDSPNTKFCYYNNYSLSQPRHFCKTCRRYWTKGGALRN
VPIGGGCRKNKKIKTCSSSSRDSKDTTSGSSDIGGLKFFHGLSPAMDFQLGGLNFPRLNNTSTSTGGSIFNQ
FSTFGDISTTTTNCATNIGSSSCFNLDPLGSCSGSLLGFNNFPFSSSILKQGTTAVQEMGSMGVHHGTMASSI
ESLSSINQDLHWKLQQQRLAMLFGGENQKENNIVSSSIPFDDQNQNQNQNQNQIQIQKPQPILFQNLEISSS
KQQEDHHHQEATTFGNDNINSRKDVNITIGSHGNNLATEWFFDDSFGVNPNSTNTINENEDQNGNNWNST
IQAWNNLNQYSTLP

>CaDOF30

MVFSSFPVYLDHPNLHHLQQEPDHHQQGSPGLDNPQLPPVQPPTLVGGGAGSIRPGSMVDRARIARLPLPE
PGLKCPRCDSSNTKFCYYNNYNLSQPRHFCKNCRRYWTKGGALRNVPVGGGCRRNKRNKSTSSSVSWG
NGISCRE

>CaDOF31

MQDPSSLIYSQIKPQFPDQEVLKCPRCDSINTKFCYYNNYNLSQPRHFCKNCKRYWTKGGILRNIPVGGSS
RKNSKRSSSSSSSSKQSLRTSSLSPSSAQNGKIEVFPTPAVPAFDQESPILDANGPFGPFLVSNGSEIGNLLEGL
NPNGPNYSGSDDAAAQSENNSTRDDEYLDVQKGEDSNCWNVNTNSWADLAIYTPGSRFQ

>CaDOF32

MTLEASEKRVTKQQTAGGVLPPAQEPDQQLPCPRCDSTNTKFCYYNNYNLSQPRHFCKSCRRYWTHGGT
LRDIPIGGGSRKNAKRCRIYTSTPFSSSTAVSPHGNSPFLVPLPTANQLLYGSDVKPCLNMSGNNFTSLLSSH
GPTGVLALGGIEDVSFSIGRAAVWPFPGAPDSFIRNYDSGVGASMWQFSG

>CaDOF33

MQDIHPIGGGGGRFYGGGGDRRLRPNNHQNHQALKCPRCGSLNTKFCYYNNYNLSQPRHFCKSCRRYW
TKGGVLRNVPVGGGCRKSKRAKLKSSSINAVAEVAEAQEEQKSDTNSSSESSSLTATTTAATAAAAVAEVTA
TGNTSGGATTEDVSATSSNSASTYLNFPDSSNFFIPHSTTDNHHTFDDQPLTENFSSMMTSSNDPSMVGFNI
AEIPAYRLPENQSSMIETLPSGDLKMEQTGTGYLNQTDRVEFPGLQQNRIDNSELASLDWQTGGGGDHGL
YDLTGTVDQSYWNQTQWGENDNSLNFLP
