# Supplementary material for: Genome-Wide Identification and Expression Profile of Dof Transcription Factor Gene Family in Pepper (Capsicum annuum L.)
Source: Front Plant Sci. 2016 Apr 29;7:574. doi: 10.3389/fpls.2016.00574 (PMC4850169; doi:10.3389/fpls.2016.00574)
Supplement: Additional file 3 — The amino acid sequences of Dof genes in Arabidopsis (At), rice (Os), and tomatoes (Sl) used in phylogenetic tree construction. [file DataSheet3.DOC]

**Table S3 The full length DOF protein sequences in *Arabidopsis thaliana* (*AtDof1.1-5.8*), *Oryza sativa* (*OsDof1-30*), *Sorghum bicolar* (*SbDof 1-28*), *Solanum lycopersicum* (*SlDof1-34*), *Chlamydomonas reinhardtii* (*CrDof*), *Physcomitrella patens* (*PpDof1-23*) and *Vitis vinifera* (*VvDof 1-22*) used in phylogenetic tree construction.**

>AtDof1.1

MGGSMAERARQANIPPLAGPLKCPRCDSSNTKFCYYNNYNLTQPRHFCKGCRRYWTQGGALRNVPVGGGCRRNNKKGKNGNLKSSSSSSKQSSSVNAQSPSSGQLRTNHQFPFSPTLYNLTQLGGIGLNLAATNGNNQAHQIGSSLMMSDLGFLHGRNTSTPMTGNIHENNNNNNNENNLMASVGSLSPFALFDPTTGLYAFQNDGNIGNNVGISGSSTSMVDSRVYQTPPVKMEEQPNLANLSRPVSGLTSPGNQTNQYFWPGSDFSGPSNDLL

>AtDof1.2

MLPYIGHNSYQQHQFPLPEMEIPEKWKLSYEQEAITAPACPRCASSNTKFCYYNNYSLSQPRYFCKGCRRYWTKGGSLRNIPVGGGCRKRSRSRQNSHKRFGRNENRPDGLINQDDGFQSSPPGSDIDLAAVFAQYVTDRSPSSTDNTTGSDQDSPITTTTHALESLSWDICQETDVDLGFYGEFNNLTQKTKEDQEVFGQFLQEDREEIFEFQGLLDDKEIQEILECSFSEEPDQLVSQGSFMINGDNWSSTDLTRFGI

>AtDof1.3

MWLSHLFMSLSKLTCNFSIFSVFMACGSIGMSQVRDTPVKLFGWTITPVSHDPYSSSSHVLPDSSSSSSSSSLSLRPHMMNNQSVTDNTSLKLSSNLNNESKETSENSDDQHSEITTITSEEEKTTELKKPDKILPCPRCNSADTKFCYYNNYNVNQPRHFCRKCQRYWTAGGSMRIVPVGSGRRKNKGWVSSDQYLHITSEDTDNYNSSSTKILSFESSDSLVTERPKHQSNEVKINAEPVSQEPNNFQGLLPPQASPVSPPWPYQYPPNPSFYHMPVYWGCAIPVWSTLDTSTCLGKRTRDETSHETVKESKNAFERTSLLLESQSIKNETSMATNNHVWYPVPMTREKTQEFSFFSNGAETKSSNNRFVPETYLNLQANPAAMARSMNFRESI

>AtDof1.4

MPIISSPNTNPLASMQSKNMIVASSHQQQQQQQPQQPQPQLKCPRCDSSNTKFCYYNNYSLSQPRHFCKACKRYWTRGGTLRNVPVGGSYRKNKRVKRPSTATTTTASTVSTTNSSSPNNPHQISHFSSMNHHPLFYGLSDHMSSCNNNLPMIPSRFSDSSKTCSSSGLESEFLSSGFSSLSALGLGLPHQMSHDHTINGSFINNSTTNKPFLLSGLFGSSMSSSSTLLQHPHKPMNNGGDMLGQSHLQTLASLQDLHVGGNNEDMKYKEGKLDQISGNINGFMSSSSSLDPSNYNNMWNNASVVNGAWLDPTNNNVGSSLTSLI

>AtDof1.5

MATQDSQGIKLFGKTITFNANITQTIKKEEQQQQQQPELQATTAVRSPSSDLTAEKRPDKIIPCPRCKSMETKFCYFNNYNVNQPRHFCKGCQRYWTAGGALRNVPVGAGRRKSKPPGRVGGFAELLGAATGAVDQVELDALLVEEWRAATASHGGFRHDFPVKRLRCYTDGQSC

>AtDof1.6

MPSEPNQTRPTRVQPSTAAYPPPNLAEPLPCPRCNSTTTKFCYYNNYNLAQPRYYCKSCRRYWTQGGTLRDVPVGGGTRRSSSKRHRSFSTTATSSSSSSSVITTTTQEPATTEASQTKVTNLISGHGSFASLLGLGSGNGGLDYGFGYGYGLEEMSIGYLGDSSVGEIPVVDGCGGDTWQIGEIEGKSGGDSLIWPGLEISMQTNDVK

>AtDof1.7

MQDLTSAAAYYHQSMMMTTAKQNQPELPEQEQLKCPRCDSPNTKFCYYNNYNLSQPRHFCKNCRRYWTKGGALRNIPVGGGTRKSNKRSGSSPSSNLKNQTVAEKPDHHGSGSEEKEERVSGQEMNPTRMLYGLPVGDPNGASFSSLLASNMQMGGLVYESGSRWLPGMDLGLGSVRRSDDTWTDLAMNRMEKN

>AtDof1.8

MDTAKWPQEFVVKPMNEIVTNTCLKQQSNPPSPATPVERKARPEKDQALNCPRCNSLNTKFCYYNNYSLTQPRYFCKDCRRYWTAGGSLRNIPVGGGVRKNKRSSSNSSSSSPSSSSSSKKPLFANNNTPTPPLPHLNPKIGEAAATKVQDLTFSQGFGNAHEVKDLNLAFSQGFGIGHNHHSSIPEFLQVVPSSSMKNNPLVSTSSSLELLGISSSSASSNSRPAFMSYPNVHDSSVYTASGFGLSYPQFQEFMRPALGFSLDGGDPLRQEEGSSGTNNGRPLLPFESLLKLPVSSSSTNSGGNGNLKENNDEHSDHEHEKEEGEADQSVGFWSGMLSAGASAAASGGSWQ

>AtDof1.10

MSKSRDTEIKLFGRTITSLLDVNCYDPSSLSPVHDVSSDPSKEDSSSSSSSCSPTIGPIRVPVKKSEQESNKFKDPYILSDLNEPPKAVSEISSPRSSKNNCDQQSEITTTTTTSTTSGEKSTALKKPDKLIPCPRCESANTKFCYYNNYNVNQPRYFCRNCQRYWTAGGSMRNVPVGSGRRKNKGWPSSNHYLQVTSEDCDNNNSGTILSFGSSESSVTETGKHQSGDTAKISADSVSQENKSYQGFLPPQVMLPNNSSPWPYQWSPTGPNASFYPVPFYWGCTVPIYPTSETSSCLGKRSRDQTEGRINDTNTTITTTRARLVSESLRMNIEASKSAVWSKLPTKPEKKTQGFSLFNGFDTKGNSNRSSLVSETSHSLQANPAAMSRAMNFRESMQQ

>AtDof2.1

MDPEQEISNETLETILVSSTKGSNNNNKKMEEEMKKKVSRGELGGEAQNCPRCESPNTKFCYYNNYSLSQPRYFCKSCRRYWTKGGTLRNVPVGGGCRRNKRSSSSAFSKNNNNKSINFHTDPLQNPLITGMPPSSFGYDHSIDLNLAFATLQKHHLSSQATTPSFGFGGDLSIYGNSTNDVGIFGGQNGTYNNSLCYGFMSGNGNNNQNEIKMASTLGMSLEGNERKQENVNNNNNNSENPSKVFWGFPWQMTGDSAGVVPEIDPGRESWNGMVSSWNNGLLNTPLV

>AtDof2.2

MVFSSVSSFLDPPINWPQSANPNNHPHHHQLQENGSLVSGHHQVLSHHFPQNPNPNHHHVETAAATTVDPSSLNGQAAERARLAKNSQPPEGALKCPRCDSANTKFCYFNNYNLTQPRHFCKACRRYWTRGGALRNVPVGGGCRRNKKGKSGNSKSSSSSQNKQSTSMVNATSPTNTSNVQLQTNSQFPFLPTLQNLTQLGGIGLNLAAINGNNGGNGNTSSSFLNDLGFFHGGNTSGPVMGNNNENNLMTSLGSSSHFALFDRTMGLYNFPNEVNMGLSSIGATRVSQTAQVKMEDNHLGNISRPVSGLTSPGNQSNQYWTGQGLPGSSSNDHHHQHLM

>AtDof2.3

MATQDSQGIKLFGKTIAFNTRTIKNEEETHPPEQEATIAVRSSSSSDLTAEKRPDKIIACPRCKSMETKFCYFNNYNVNQPRHFCKGCHRYWTAGGALRNVPVGAGRRKSKPPGRVVVGMLGDGNGVRQVELINGLLVEEWQHAAAAAHGSFRHDFPMKRLRCYSDGQSC

>AtDof2.4

MVFSSIQAYLDSSNWQQAPPSNYNHDGTGASANGGHVLRPQLQPQQQPQQQPHPNGSGGGGGGGGGSIRAGSMVDRARQANVALPEAALKCPRCESTNTKFCYFNNYSLTQPRHFCKTCRRYWTRGGALRNVPVGGGCRRNRRTKSNSNNNNNSTATSNNTSFSSGNASTISTILSSHYGGNQESILSQILSPARLMNPTYNHLGDLTSNTKTDNNMSLLNYGGLSQDLRSIHMGASGGSLMSCVDEWRSASYHQQSSMGGGNLEDSSNPNPSANGFYSFESPRITSASISSALASQFSSVKVEDNPYKWVNVNGNCSSWNDLSAFGSSR

>AtDof2.5

MDATKWTQGFQEMMNVKPMEQIMIPNNNTHQPNTTSNARPNTILTSNGVSTAGATVSGVSNNNNNTAVVAERKARPQEKLNCPRCNSTNTKFCYYNNYSLTQPRYFCKGCRRYWTEGGSLRNVPVGGSSRKNKRSSSSSSSNILQTIPSSLPDLNPPILFSNQIHNKSKGSSQDLNLLSFPVMQDQHHHHVHMSQFLQMPKMEGNGNITHQQQPSSSSSVYGSSSSPVSALELLRTGVNVSSRSGINSSFMPSGSMMDSNTVLYTSSGFPTMVDYKPSNLSFSTDHQGLGHNSNNRSEALHSDHHQQGRVLFPFGDQMKELSSSITQEVDHDDNQQQKSHGNNNNNNNSSPNNGYWSGMFSTTGGGSSW

>AtDof3.1

MQDPAAYYQTMMAKQQQQQQPQFAEQEQLKCPRCDSPNTKFCYYNNYNLSQPRHFCKSCRRYWTKGGALRNVPVGGGSRKNATKRSTSSSSSASSPSNSSQNKKTKNPDPDPDPRNSQKPDLDPTRMLYGFPIGDQDVKGMEIGGSFSSLLANNMQLGLGGGGIMLDGSGWDHPGMGLGLRRTEPGNNNNNPWTDLAMNRAEKN

>AtDof3.2

MDYSSMHQNVMGVSSCSTQDYQNQKKPLSATRPAPPEQSLRCPRCDSTNTKFCYYNNYSLSQPRYFCKSCRRYWTKGGILRNIPIGGAYRKHKRSSSATKSLRTTPEPTMTHDGKSFPTASFGYNNNNISNEQMELGLAYALLNKQPLGVSSHLGFGSSQSPMAMDGVYGTTSHQMENTGYAFGNGGGGMEQMATSDPNRVLWGFPWQMNMGGGSGHGHGHVDQIDSGREIWSSTVNYINTGALL

>AtDof3.3

MMMETRDPAIKLFGMKIPFPSVFESAVTVEDDEEDDWSGGDDKSPEKVTPELSDKNNNNCNDNSFNNSKPETLDKEEATSTDQIESSDTPEDNQQTTPDGKTLKKPTKILPCPRCKSMETKFCYYNNYNINQPRHFCKACQRYWTAGGTMRNVPVGAGRRKNKSSSSHYRHITISEALEAARLDPGLQANTRVLSFGLEAQQQHVAAPMTPVMKLQEDQKVSNGARNRFHGLADQRLVARVENGDDCSSGSSVTTSNNHSVDESRAQSGSVVEAQMNNNNNNNMNGYACIPGVPWPYTWNPAMPPPGFYPPPGYPMPFYPYWTIPMLPPHQSSSPISQKCSNTNSPTLGKHPRDEGSSKKDNETERKQKAGCVLVPKTLRIDDPNEAAKSSIWTTLGIKNEAMCKAGGMFKGFDHKTKMYNNDKAENSPVLSANPAALSRSHNFHEQI

>AtDof3.4

MPTSDSGEPRRIAMKPNGVTVPISDQQEQLPCPRCDSSNTKFCYYNNYNFSQPRHFCKACRRYWTHGGTLRDVPVGGGTRKSAKRSRTCSNSSSSSVSGVVSNSNGVPLQTTPVLFPQSSISNGVTHTVTESDGKGSALSLCGSFTSTLLNHNAAATATHGSGSVIGIGGFGIGLGSGFDDVSFGLGRAMWPFSTVGTATTTNVGSNGGHHAVPMPATWQFEGLESNAGGGFVSGEYFAWPDLSITTPGNSLK

>AtDof3.5

MERAEALTSSFIWRPNANANAEITPSCPRCGSSNTKFCYYNNYSLTQPRYFCKGCRRYWTKGGSLRNVPVGGGCRKSRRPKSSSGNNTKTSLTANSGNPGGGSPSIDLALVYANFLNPKPDESILQENCDLATTDFLVDNPTGTSMDPSWSMDINDGHHDHYINPVEHIVEECGYNGLPPFPGEELLSLDTNGVWSDALLIGHNHVDVGVTPVQAVHEPVVHFADESNDSTNLLFGSWSPFDFTADG

>AtDof3.6

MVFSSLPVNQFDSQNWQQMISILVFFSTSRLFKKLFLVDKNLFSCLLQGLMYNVFLTGLIFSLQGNQHQLECVTTDQNPNNYLRQLSSPPTSQVAGSSQARVNSMVERARIAKVPLPEAALNCPRCDSTNTKFCYFNNYSLTQPRHFCKTCRRYWTRGGSLRNVPVGGGFRRNKRSKSRSKSTVVVSTDNTTSTSSLTSRPSYSNPSKFHSYGQIPEFNSNLPILPPLQSLGDYNSSNTGLDFGGTQISNMISGMSSSGGILDAWRIPPSQQAQQFPFLINTTGLVQSSNALYPLLEGGVSATQTRNVKAEENDQDRGRDGDGVNNLSRNFLGNININSGRNEEYTSWGGNSSWTGFTSNNSTGHLSF

>AtDof3.7

MDATKWTQGFQEMINVKPMEQMISSTNNNTPQQQPTFIATNTRPNATASNGGSGGNTNNTATMETRKARPQEKVNCPRCNSTNTKFCYYNNYSLTQPRYFCKGCRRYWTEGGSLRNVPVGGSSRKNKRSSTPLASPSNPKLPDLNPPILFSSQIPNKSNKDLNLLSFPVMQDHHHHGMSHFFHMPKIENNNTSSSIYASSSPVSALELLRSNGVSSRGMNTFLPGQMMDSNSVLYSSLGFPTMPDYKQSNNNLSFSIDHHQGIGHNTINSNQRAQDNNDDMNGASRVLFPFSDMKELSSTTQEKSHGNNTYWNGMFSNTGGSSW

>AtDof4.1

MDHHQYHHHDQYQHQMMTSTNNNSYNTIVTTQPPPTTTTMDSTTATTMIMDDEKKLMTTMSTRPQEPRNCPRCNSSNTKFCYYNNYSLAQPRYLCKSCRRYWTEGGSLRNVPVGGGSRKNKKLPFPNSSTSSSTKNLPDLNPPFVFTSSASSSNPSKTHQNNNDLSLSFSSPMQDKRAQGHYGHFSEQVVTGGQNCLFQAPMGMIQFRQEYDHEHPKKNLGFSLDRNEEEIGNHDNFVVNEEGSKMMYPYGDHEDRQQHHHVRHDDGNKKREGGSSNELWSGIILGGDSGGPTW

>AtDof4.2

MNNLNVFTNEDNEMNVMPPPRVCPRCYSDQTRFSYFNNNKKSQPRYKCKNCCRCWTHGGVLRNIPVTGICDKSNLPKIDQSSVSQMILAEIQQGNHQPFKKFQENISVSVSSSSDVSIVGNHFDDLSELHGITNSTPIRSFTMDRLDFGEESFQQDLYDVGSNDLIGNPLINQSIGGYVDNHKDEHKLQFEYES

>AtDof4.3

MDNFNVVANEDNQVNDVKPPPPPPRVCARCDSDNTKFCYYNNYSEFQPRYFCKNCRRYWTHGGALRNVPIGGSSRAKRTRINQPSVAQMVSVGIQPGSHKPFFNVQENNDFVGSFGASSSSFVAAVGNRFSSLSHIHGGMVTNVHPTQTFRPNHRLAFHNGSFEQDYYDVGSDNLLVNQQVGGYVDNHNGYHMNQVDQYNWNQSFNNAMNMNYNNASTSGRMHPSHLEKGGP

>AtDof4.4

MDNLNVFANEDNQVNGLKRPPPSRVCPRCDSDNTKFCFYNNYSESQPRYFCKNCRRYWTHGGALRNIPVGGSCRKPKRLKVDQSSISEMVSVENQPINHQSFRQTQENNEFVRSFDASSSATVTAVPNHFGYLSELHGVTNLLPIQSFRTMDCLDFGDESFQQGYYDVGSNDLIDNPLINQSIGGYVDNLTSYCINQVEPKLQPRYEHES

>AtDof4.5

MDNLNVFANEDNQVNDVKPPPPPPRVCARCDSDNTKFCYYNNYCEFQPRYFCKNCRRYWTHGGALRNIPIGGSSRAKRARVNQPSVARMVSVETQRGNNQPFSNVQENVHLVGSFGASSSSSVGAVGNLFGSLYDIHGGMVTNLHPTRTVRPNHRLAFHDGSFEQDYYDVGSDNLLVNQQVGGYGYHMNPVDQFKWNQSFNNTMNMNYNNDSTSGSSRGSDMNVNHDNKKIRYRNSVIMHPCHLEKDGP

>AtDof4.6

MDTAQWPQEIVVKPLEEIVTNTCPKPQPQPLQPQQPPSVGGERKARPEKDQAVNCPRCNSTNTKFCYYNNYSLTQPRYFCKGCRRYWTEGGSLRNIPVGGGSRKNKRSHSSSSDISNNHSDSTQPATKKHLSDHHHHLMSMSQQGLTGQNPKFLETTQQDLNLGFSPHGMIRTNFTDLIHNIGNNTNKSNNNNNPLIVSSCSAMATSSLDLIRNNSNNGNSSNSSFMGFPVHNQDPASGGFSMQDHYKPCNTNTTLLGFSLDHHHNNGFHGGFQGGEEGGEGGDDVNGRHLFPFEDLKLPVSSSSATINVDINEHQKRGSGSDAAATSGGYWTGMLSGGSWC

>AtDof4.7

MMTSSHQSNTTGFKPRRIKTTAKPPRQINNKEPSPATQPVLKCPRCDSVNTKFCYYNNYSLSQPRHYCKNCRRYWTRGGALRNVPIGGSTRNKNKPCSLQVISSPPLFSNGTSSASRELVRNHPSTAMMMMSSGGFSGYMFPLDPNFNLASSSIESLSSFNQDLHQKLQQQRLVTSMFLQDSLPVNEKTVMFQNVELIPPSTVTTDWVFDRFATGGGATSGNHEDNDDGEGNLGNWFHNANNNALL

>AtDof5.1

MVFSSFPTYPDHSSNWQQQHQPITTTVGFTGNNINQQFLPHHPLPPQQQQTPPQLHHNNGNGGVAVPGGPGGLIRPGSMAERARLANIPLPETALKCPRCDSTNTKFCYFNNYSLTQPRHFCKACRRYWTRGGALRSVPVGGGCRRNKRTKNSSGGGGGSTSSGNSKSQDSATSNDQYHHRAMANNQMGPPSSSSSLSSLLSSYNAGLIPGHDHNSNNNNILGLGSSLPPLKLMPPLDFTDNFTLQYGAVSAPSYHIGGGSSGGAAALLNGFDQWRFPATNQLPLGGLDPFDQQHQMEQQNPGYGLVTGSGQYRPKNIFHNLISSSSSASSAMVTATASQLASVKMEDSNNQLNLSRQLFGDEQQLWNIHGAAAASTAAATSSWSEVSNNFSSSSTSNI

>AtDof5.2

MADPAIKLFGKTIPLPELGVVDSSSSYTGFLTETQIPVRLSDSCTGDDDDEEMGDSGLGREEGDDVGDGGGESETDKKEEKDSECQEESLRNESNDVTTTTSGITEKTETTKAAKTNEESGGTACSQEGKLKKPDKILPCPRCNSMETKFCYYNNYNVNQPRHFCKKCQRYWTAGGTMRNVPVGAGRRKNKSPASHYNRHVSITSAEAMQKVARTDLQHPNGANLLTFGSDSVLCESMASGLNLVEKSLLKTQTVLQEPNEGLKITVPLNQTNEEAGTVSPLPKVPCFPGPPPTWPYAWNGVSWTILPFYPPPAYWSCPGVSPGAWNSFTWMPQPNSPSGSNPNSPTLGKHSRDENAAEPGTAFDETESLGREKSKPERCLWVPKTLRIDDPEEAAKSSIWETLGIKKDENADTFGAFRSSTKEKSSLSEGRLPGRRPELQANPAALSRSANFHESS

>AtDof5.3

MDHLLQHQDVFGNYNKAREAMGLSYSSNPTPLDNDQKKPSPATAVTRPQPPELALRCPRCDSTNTKFCYYNNYSLTQPRYFCKSCRRYWTKGGTLRNIPVGGGCRKNKRSTSSAARSLRTTPEPASHDGKVFSAAGFNGYSNNEHIDLSLAFALLNKQHPGSSSQLGFHSELGSSHQSDMEGMFGTSQQKENATYAFGNGSSGLGDPSRVLWGFPWQMNGESFGMMNIGGGGGHVDQIDSGREMWTNMNYINSGALM

>AtDof5.4

MQDIHDFSMNGVGGGGGGGGRFFGGGIGGGGGGDRRMRAHQNNILNHHQSLKCPRCNSLNTKFCYYNNYNLSQPRHFCKNCRRYWTKGGVLRNVPVGGGCRKAKRSKTKQVPSSSSADKPTTTQDDHHVEEKSSTGSHSSSESSSLTASNSTTVAAVSVTAAAEVASSVIPGFDMPNMKIYGNGIEWSTLLGQGSSAGGVFSEIGGFPAVSAIETTPFGFGGKFVNQDDHLKLEGETVQQQQFGDRTAQVEFQGRSSDPNMGFEPLDWGSGGGDQTLFDLTSTVDHAYWSQSQWTSSDQDQSGLYLP

>AtDof5.5

MLETKDPAIKLFGMKIPFPTVLEVADEEEEKNQNKTLTDQSEKDKTLKKPTKILPCPRCNSMETKFCYYNNYNVNQPRHFCKACQRYWTSGGTMRSVPIGAGRRKNKNNSPTSHYHHVTISETNGPVLSFSLGDDQKVSSNRFGNQKLVARIENNDERSNNNTSNGLNCFPGVSWPYTWNPAFYPVYPYWSMPVLSSPVSSSPTSTLGKHSRDEDETVKQKQRNGSVLVPKTLRIDDPNEAAKSSIWTTLGIKNEVMFNGFGSKKEVKLSNKEETETSLVLCANPAALSRSINFHEQM

>AtDof5.6

MGLTSLQVCMDSDWLQESESSGGSMLDSSTNSPSAADILAACSTRPQASAVAVAAAALMDGGRRLRPPHDHPQKCPRCESTHTKFCYYNNYSLSQPRYFCKTCRRYWTKGGTLRNIPVGGGCRKNKKPSSSNSSSSTSSGKKPSNIVTANTSDLMALAHSHQNYQHSPLGFSHFGGMMGSYSTPEHGNVGFLESKYGGLLSQSPRPIDFLDSKFDLMGVNNDNLVMVNHGSNGDHHHHHNHHMGLNHGVGLNNNNNNGGFNGISTGGNGNGGGLMDISTCQRLMLSNYDHHHYNHQEDHQRVATIMDVKPNPKLLSLDWQQDQCYSNGGGSGGAGKSDGGGYGNGGYINGLGSSWNGLMNGYGTSTKTNSLV

>AtDof5.7

MSSHTNLPSPKPVPKPDHRISGTSQTKKPPSSSVAQDQQNLKCPRCNSPNTKFCYYNNYSLSQPRHFCKSCRRYWTRGGALRNVPIGGGCRKTKKSIKPNSSMNTLPSSSSSQRFFSSIMEDSSKFFPPPTTMDFQLAGLSLNKMNDLQLLNNQEVLDLRPMMSSGRENTPVDVGSGLSLMGFGDFNNNHSPTGFTTAGASDGNLASSIETLSCLNQDLHWRLQQQRMAMLFGNSKEETVVVERPQPILYRNLEIVNSSSPSSPTKKGDNQTEWYFGNNSDNEGVISNNANTGGGGSEWNNGIQAWTDLNHYNALP

>AtDof5.8

MPSEFSESRRVPKIPHGQGGSVAIPTDQQEQLSCPRCESTNTKFCYYNNYNFSQPRHFCKSCRRYWTHGGTLRDIPVGGVSRKSSKRSRTYSSAATTSVVGSRNFPLQATPVLFPQSSSNGGITTAKGSASSFYGGFSSLINYNAAVSRNGPGGGFNGPDAFGLGLGHGSYYEDVRYGQGITVWPFSSGATDAATTTSHIAQIPATWQFEGQESKVGFVSGDYVA

>OsDof1

MDDLAAASPPHPPPPPPESHVPPPPQTPEKDSCEDTGDMRISEEKPCTDQELDADQMNSSSFNSSSECENQTPSNDEMTGSESKSEAAQTEGGGSSEEKVLKKPDKILPCPRCNSMDTKFCYYNNYNINQPRHFCKSCQRYWTAGGSMRNLPVGAGRRKSKSSTANYRSILITGSNLAAPAGDAPLYQLSIKGDQTATAVKFAPDSPLCNSMASVLKIGEQSKNAKPTSTAQPRNGETQTCPASGTTSDSPRNEPVNGAVSGHQNGIVGHSGVPPMHPIPCFPGPPFVYPWSPAWNGIPAMAPPVCTAPAEPANSSDNGSTASVQWSMPPVMPVPGYFPVIPSSVWPFISPWPNGAWSSPWIQPNCSVSASSPTSTSTCSDNGSPVLGKHSRDSKPQGDDKAEKNLWIPKTLRIDDPDEAAKSSIWTTLGIEPGDRSMFRSFQSKPESREQISGAARVLQANPAALSRSQSFQETT

>OsDof2

MLSHVEMAPAAGGFKLFGKVIMQCGVSEGTQDKAQGFVVAREKVEPEEEEEEEQRVPAAATSGQRASIKREAADRDEEQRQGGGDAAGQPTQRRLQDSAEARAAAAAPLPCPRCRSRDTKFCYFNNYNVNQPRHFCKACHRYWTAGGALRNVPVGAGRRKNRPLGPLAVAHHNHHHRAAAGFVLGFPNPSSPTSPSPVYTDRWPVTPDRPF

>OsDof3

MCDKDPGIKLFGRVIPLAPEAEAAAAADGSDQPEAAAAAAAEVEPAAQDEDHHKETEERKYDEMKVDVPQEEEDNEMKVDAPQEKKDNEVTADVPEEKGNDEMRVDASESIESIEPVSRSTLDNKKEDQGQMNNVEEKAASDSKDENEKTANDESGQDKVLKKPDKILPCPRCNSMDTKFCYYNNYNVNQPRHFCKNCQRYWTAGGTMRNVPVGAGRRKSKSSSLHYRHLLMAPDCMMGSRVEISKSMNPEAFASAHSTPIQPIGRNETVLKFGPEVPLCESMASVLNIQEQNGTNAAAVPTGENQEDNSCISSITSHNVLPENAAQVDKNSTPVYCNGVGPVPQYYLGAPYMYPWNIGWNNVPMMVPGTSMPESASQSESCSTSSAPWMNMNSPMMPVASRLSAPPFPYPLVPPALWGCLSSWPATAWNIPWIRTNGGCMSPSSSSNSSCSGNGSPLGKHSRDSSLPLKEDKEEKSLWVPKTLRIDDPDEAAKSSIWATLGIKPGDPGIFKPFQSKGESKGQAASETRPARALKANPAALSRSQSFQETS

>OsDof4

MAALRQGDDPAIKLFGRTIPLLLDPPAAAAAAADEVMPNLGNGVKTNNDLPLVSDKLLIVKGIPFCPNNSKKNDLQGISRPDGRIEIDSMTEDVKTEPDGSVPEKILKKPDKILPCPRCNSMETKFCYFNNYNVHQPRHFCRNCQRYWTAGGAMRNVPVGAGRRRNKHVSKYCQAMMTCNNTVAPGDVSDVVHHQVITHGSSLLPATLKENETPTEFISEVPPCKSSASILDIGEPNDTDLVPLASGDNKEEKSCASSVVVSSCSENLMPDNAIMKEPNNRSGCCNGVALPFPTGPALVLPWSLGWNSVALMPATQCSMQPVLGLKDGIPCPPSWPPQLMVPAPGICTPVVPIPLVPPLWSCFPGWPNGMWNAQCPGGNTTVLPSTAPNKISCSGSSSLVLGKHSREESLQEEEKTRNYLWVPKTLRIDDPAEAAKSSIWATLGIKPDDKGIFKSFQPNVAKNGTAPESPQALQANPAAFSRSQSFQETT

>OsDof5

MVFPSVPAYLDPPNWNNQQGQPPRPANVGGGDAQHLPVGPTAAAAAPGEIGGLPTSSSSASAAAAAAQQARPNSMAERARLARAPQPEPALKCPRCDSTNTKFCYYNNYSLSQPRHFCKTCRRYWTRGGSLRNVPVGGGCRRNKRSGKSSSAAAAGASSSSSKPSSSAARQLPGGGASPMPSAAASTQPGGAAAGAIIPPSGLSSMSHHLPFLGAMHPPGPNLGLTFSAGFQPLGGMHHHVDTADQFPVASGGGATIGASLEQWRVQQQQQQQPQQHQFPFLGGALELPPPPPMYQLGLEATRAAGTGATAAAAFTLGQTSATATTSRQEGSMKLEDSKGLEMSLQRQYMAALRQGDGVWGNNNGGNGGSDGGGNGGGGSWTMNFPGFHSSSGGGGDDGGGVL

>OsDof6

MGECKVGGGGGGGDCLIKLFGKTIPVPEPGACAAGDVDKDLQHSGSSTTEPKTQENTVQDSTSPPPQPEVVDTEDSSADKNSSENQQQQGDTANQKEKLKKPDKILPCPRCSSMDTKFCYYNNYNINQPRHFCKNCQRYWTAGGAMRNVPVGAGRRKSKSVSAASHFLQRVRAALPGDPPLYAPVKTNGTVLSFGSDLSTLDLTEQMKHLKDKFIPTTGIKNTDEMPVGLCAEGLSKTEESNQTNLKEKVSADRSPNVAQHPCMNGGAMWPFGVAPPPAYYTSSIAIPFYPAAAAAVAAYWGCMVPGAWNAPWPPQSQSQSVSSSSAASPVSTMTNCFRLGKHPRDGDEELDSKGNGKVWVPKTVRIDDVDEVARSSIWSLIGIKGDKVGADHGRGCKLAKVFESKDEAKASTHTAISSLPFMQGNPAALTRSVTFQEGS

>OsDof7

MGECRGGGGGGDGLIKLFGKTIPVQPDAKDVQQHSGSSSSSTESDVQETAAVAVADPSPRSEVVDGESPPQPGGEAASHQQQQKEMKLKKPDKILPCPRCSSMDTKFCYFNNYNVNQPRHFCKHCQRYWTAGGAMRNVPVGAGRRKNKNATAAAHFLHRVRACAAAAAMPAAPHDATNATVLSFGGGGGGHDAPPVTLDLADKMTRLGKEGLVAHARNADAAAACSEVSSNRDDEQIGNTVAKPANGLQQHPPPPHHHHHSAMNGGGIWPYYTSGIAIPIYPAAPAYWGCMIPPPGAWSLPWPATVQSQAISSSSPPTSATPSVSSFTLGKHPREGGDHEARDHHGNGKVWVPKTIRIDNADEVARSSIRSLFAFRGGDKADDNNDDDGTGVHKLATTVFEPKRDSKTAKHPAITSLPLLHTNPVALTRSATFQEGS

>OsDof8

MLSSHCESMLAYAAAAGRRAVVVDHHQRRYRPNVEVAPNCPRCESPNTKFCYYNNYSLSQPRYFCKGCRRYWTKGGSLRNVPVGGGCRKNRRGKAVRAMVGETMTARGGGGGGAAAFSHRFHGPVRPDMILEGMAGSTAASAGLGEQPGVAAPDEKPAAADGSTIDLALLYAKFLNHHQPTMAEQGGGAAVPESVDTSSGSSSDRTTSPAAAQPAAAAAYGPGQDGLVGEPISTEEHGAAAMARCAQALGELNFSVDQISCYTSLGLPTTDGGDLILPSTLDQHAKYEPFDSLPEDALSLHDIISGDDDVWCNALGCQGLEAALCRP

>OsDof9

MLPYAPRPPSLLVDRRYKQGAEAAPNCPRCDSPNTKFCYYNNYSLSQPRYFCKGCRRYWTKGGSLRNVPVGGGCRKNRRGKSSSSARSAADAVSSGRDAAFGHRFPGPVRPDMVLEGMVGNPANPGQAMPDVAAAADGSTIDLAMLYAKFLNHPPTDAGLGAVTPESGGHVDEAFDTFSASSDLSPGILAAASAQFDPNQDGFGEWSSPASGNDPTSTATTATTSMLCTDASVQAALGELNFAMDQSCFDSLGLPTDVAGAGSLSSWCSIVPSLSTWEEPKYDSLDSFPDDAMSLHECMIGAPDHDWSVDCQGLEALYMP

>OsDof10

MAGAGGAATAAAGGGGGGGVAAGRSGGGGGGGAAAAAGAGAPDPRAEALRCPRCDSANTKFCYYNNYSLSQPRHFCKACKRYWTRGGTLRNVPVGGGCRKNKRSRSGGAAPGGGVGRGGPGGGAAAAVSSAGGGAAGTSPASSLALPQPGSLPSLSSALGLTGGTSLASLLLGSGGSGGDHLGLFQAMQSVVSDAAAFEMHQQHQSQVDHLLGLGYGAAGAQIQAAKPWLHDGGATGGLLDGFYAPLLSGSIVPGLEELQVKAEATTGDHQQKSSAAAAGEQSWDLPTPSSSNVEASIIASDALMAAAAASMNPAVSAAAASTAPSAQSLLYWGNGGIGAAAAAWPDLANCGSSIATLF

>OsDof11

MMAGAPPMHICMDSDWLKGIVPEEHGMGSSSPSAELIACPRAPMQAAAAAADRRLRPQHDQPLKCPRCESTHTKFCYYNNYSLSQPRYFCKTCRRYWTKGGSLRNVPVGGGCRKNKRAPPKKAAAHAQPAVAVAAALQGRHMETGLHLSFSGMQHHLAPPPPAAATAADPLCSLGLFDWKQYDPVFAGSGGGGSPVAALESAGGSEAQFMGAGMMGIGGGGVAEYHALSALRFAAGLGDHLALPFGAVRAEHDAVEVKPVAAERLLSLEWCGEASRTAAPESSISSLGGLGLWSGMIGGGHHHHGSSAAI

>OsDof12

MQEFQSIPGLAGRLFGGAAAADIRRAQAQQGPASRCGGIPSPEAVKCPRCESTNTKFCYYNNYNLSQPRHFCKSCRRYWTKGGVLRNVPVGGGCRKTKRSGSSSAASSAPSTPTAATDNAKNQRRASASSPRSSSGGSGNTSPTAAAATTPTTPATPSSNTIAVINHATTTTTTTNPFPTDVPPPAPIFADQAAALASLFAPPPPPPLPVFSFAAQAKTEDGIASVLLAGQTTAPTAATVADMTPFTSLDAGIFELGDVPPAAYWNAGSCWTDVPDPNVYLP

>OsDof13

MAPAAGDDAVVPRKGAGGGGTTTPPPPPPAQQQQQQPLPPPPPQEQGLRCPRCDSPNTKFCYYNNYSLSQPRHFCKTCRRYWTKGGALRNVPVGGGCRKNKRSRSAAAASRLSLNLPTVEGIGGAAADPAAAAAARLGFLGGGTTMMMSSSTSPLGGAAAAVADFQQGGAVGMLPLPRLQSPAGGGVGHHQYVPFGEWPSGDIAGGNAVNGGGGHGAVSSTIASSIESLSFINQDLHWKLQQQRLATMFLGPPGSASAAAAAAQANDGGGNGAQATASGHAAGGGGGAFMHMAGSVPSMEAAMPSATSWFMDSCSYGLPSPPPPATAAVAATTSSNLNSGGRSSGGDDNATSNCGSAIPAWGDISTFAMLP

>OsDof14

MQEFHPVPGLAGRLFGGAAAAAAVAAVEEVRCPRCDSSNTKFCYYNNYNLSQPRHFCKACRRYWTKGGLLRNVPVGGGCRKPKRPAPPPSSSFTGGGGGGGGCGHRDSKSARSAGGGGDGSGSTASATATPAAAPASSNTLSAAVSQPSSVDALSPPPAPMFADQATAFASLFAPPPPPPSQALPAFASFTAQPKAEEDVADAPALAATEQHRSSSAASFAAHSISPPFAAARSSDGPAAAAAAAAADWAPPTAVLDAGMFDLAGAIGGDTSYWNAASWTDHDGTIYLP

>OsDof15

MEVAEGRTVAAAAAGGGGLGGGARTEAEGLACPRCESTNTKFCYYNNYNLAQPRHFCKACRRYWTRGGALRNVPVGGGTRNKVAPAPCTGRRKRAAHAAHAAAPPPTTTASSAPLPLMPPAVAYELPFLPPPPPLPLAAVDPDRRLLDLGGSFTSLLAPAQLHNGHFTTGFLLGTMSSAPPPPPPPATSTPSPAPAAHPPVSDSIWAMGWPHLSI

>OsDof16

MDAAHWHQGLGLVKPMEEMLMAANAAAGANPNPAATAPSSVTGGALRGGGGGGAPPVAGGAGAGSTERRARPQKEKALNCPRCNSTNTKFCYYNNYSLQQPRYFCKTCRRYWTEGGSLRNVPVGGGSRKNKRSSSSAASASPASASTANSVVTSASMSMSMASTGGGASKNPKLVHEGAQDLNLAFPHHGGLQAPGEFPAFPSLESSSVCNPGGPMGTNGRGGGALSAMELLRSTGCYMPLQVPMQMPAEYATPGFALGEFRAPPPPPQSSQSLLGFSLDAHGSVGGPSAAGFGSSAGLQGVPESTGRLLFPFEDLKPTVSSGTGGGGASGGGAGVDGGHQFDHGKEQQAGGGGGGPGGHDTPGFWNGMIGGGSGTSW

>OsDof17

MDAAHWHQGLGLVKPMEEMLMGANPNPNGSSNQPPPPPSSAASAQRPIAPPAAGAAAGAGAAGAGAGTERRARPQKEKALNCPRCNSTNTKFCYYNNYSLQQPRYFCKTCRRYWTEGGSLRNVPVGGGSRKNKRSSSSVVPSAAASASTSAAVSGSVPVGLAAKNPKLMHEGAQDLNLAFPHHHGRALQPPEFTAFPSLESSSVCNPGGNLAAANGAGGRGSVGAFSAMELLRSTGCYVPLPQMAPLGMPAEYAAAGFHLGEFRMPPPPQQQQQQQAQTVLGFSLDTHGAGAGGGSGVFGACSAGLQESAAGRLLFPFEDLKPVVSAAAGDANSGGDHQYDHGKNQGGGGGVIGGHEAPGFWNSSMIGNGSSNGGGGGGSW

>OsDof18

MQEQQPETGRRPAQQFATVDLRRPKGYAAAPATPQPGSAATAAAAAGPAATAAAAAAGEGDPCPRCESRDTKFCYYNNYNTSQPRHFCKCCRRYWTKGGTLRNVPVGGGTRKKSSSSSSSSSSSSAAAAAPAAKRQKTSKKRRVTTPEPLAATTPVLTEAAADSAAKTTTEATSEKKTTTSTTTTTPPAPDTTSEITTELVVPAVEEDSFTDLLQPDSAAVTLGLDFSDYPSITKSLADPDLHFEWPPPAFDMASYWPAGAGFADPDPTAVFLNLP

>OsDof19

MPGQVMEAALQQLPASMASGSLLLPPACLQHPLPAAAAASGGVGGSSREQCPRCASHDTKFCYYNNYNTSQPRHFCRACRRYWTLGGSLRNVPIGGSTRKRPRPPVRRPPVHFTAAAAAAAAAAPPHHHHHHHGGPLTPPPATSSSSQQAGLLGSLFALGAAPLLEGRVGVGFDLGLGLPGPGHHHAVAGGGGPAAAVATSSSSSAAAPLLWPTGLLDSSSNNAETWRMAAGGMWPEFTAAAAQNIRLVIDIGDTTIQVPLNGPTVVQNIGRQAAAAVAGDSSAGGVSEKTGGAGGGGGEEWMQEQDGLLCMRGRRCGRRGGCLPRPRDWFAALLAADPAAAAVTRDQAGKAMLYLIVNTCTFATSLAVLPDAVRRRRRLRVEEGHRVADHQHDDGRRAVRRGDVRALRRRRLPAHGPFVGTVVAAVTVVVVRCNLALPFRGGDAGHGCSWVSRL

>OsDof20

MANLPSTAAAAAADASGFKLFGKVIQPDGQRGVEESAAAQAPPHPHPPAPPVMEAAAAAAGTSQTLQAAGGGGGGGGGGGGEPLPCPRCGSRETKFCYFNNYNVRQPRHLCRSCRRYWTAGGALRRVASASPGRRRPRPSAARSAAAAAASASAASPPAAVPAASEGAESVDSRS

>OsDof21

MVFSSLPIFLDPPNWTQMQQQPLQCLIGGGGSDHHHLMPPPSGLAPLPSAAGAADTAASAPAAAAQQQQPRPAVVSMSERARLARVPLPEPGTLRCPRCDSTNTKFCYFNNYSLSQPRHFCKACRRYWTRGGALRNVPVGGGCRRNTKRSTKKSSSSSSRQGGGAGNAAAAATSSSSTTSTSTTATTSSAAAAAAAAAADVIASMQAGGALLPHHLIGGLPSSAAAAAALEASLEGYHHHHHAHGHQLPFLQPPPFLQQGLHGYHFADGDVAAGAALADGGFPRGVASGLLAQLASVKMEEHGTNNGGGVGGGFVGAHEQYWHGGNGGGGWPAEFLSGFSSSSSGNVL

>OsDof22

MTSASLLLAPRSPDMAAAGILPVSGGGGASSARPVSMAERARMAKIPQPEPGLKCPRCDSTNTKFCYFNNYSLTQPRHFCKACRRYWTRGGALRNVPVGGGFRRNKRGTKPSNSKKPAAAVAGGVMAPPHAQLQLPFGFDGGGGGGHGSIIGGGGGGGASRLGFPELSSLHAAAAVDYQLGGGGGGDGLGLERQRLPHFPFLARSNAAVHPPPLMSTAAGVSYPFGDVAAGGLGGDMPANAASVAGSAGLITQMASVKMDDIDNHPPPSAATTTASSPIEFLGLRGSLQFWGGGGGHRGGGDGAGGSAAPGGGGGGWSDLPAFDLSTSGNIL

>OsDof23

MASGGALSPVEEKPTVVKTTKAEQHEEEAAVAVKSAAEMMKKSSPCCPRCNSIKTKFCYYNNYSMAQPRYFCRECRRYWTQGGSLRNVPVGGGCRKSKRSSASSASASAASPPAPAVGAAPPVVPALSSAISKLLQSEPMAAPCADFPNVLPTFVSTGFELPAAAGDRLSLGSFGAFGNLSAAVAAPGGGGGSSTTTSFMDMLRGVGGLFDGVGNSHQMGGNGGGGGSYYAPLITGAGNGMLMPPPPLPPFSGSLMQHGMQGLFANHAMGGGGGGVMNAGEDGSVMAGLGGGQWPPALGGADEQQGGGDGGEAVMTKDTGGGASSSASRPDYFYGWNSAAGGVVAGGGIGGNAAAATGATPWQGLIDSSSAMM

>OsDof24

MIFPPAFLDSSSWNDNNNNNHNQQQQQQHAHGHHQHHQVAAGCGGGGGGGDGNSHELLQQQSMIPGTLADGGGGGGAVGPAKPMSMSERARLARIPLPEPGLKCPRCDSTNTKFCYFNNYSLSQPRHFCRACRRYWTRGGALRNVPVGGGYRRHAKRAKPKPASAAGSASAATTTAGSTPAGSTTTTTTSSTCATPNAPALPAMLGGNLSILPPLLRLADFDAMSLGSTFSGMAAAAGKPPPVDAAGCYSVGAATGLEQWRLQQMQSFPFFHAMDHQAAMAAPPPAMAMPGMFQLGLDGDGHGSGGGEDGGELHHAMPSSKREGYPRGMYGDHHLAGGYTSYSSATTGNHLL

>OsDof25

MAPAVASSPSLVLSAAAATASNKRPADSDASPPHQGDRTGQQEKKQQQLECPRCRSTNTKFCYYNNYSTSQPRHFCRACRRYWTHGGTLRDVPVGGASRRGGGGKRRRVSADADPSSASPPPPTTSTTDAYADLPAGFPFLSDGAFLPQFGLAGVAPAAFSWASAVPDLYNCGIAPWDDGTAVTGAAWDNFADIAGLDLSWPPPGN

>OsDof26

MSSPFLGSSSSSASSPLSYLTPPRPPPPPPPLLMVRHGLARWLTDLDDELMVFDDDLGVQGQGYAAAANGGIGGGGVEAVNAAAAPRQGGRHAGHPPLPRPPPRQCPRCGSANTKFCYYNNYSRTQPRYLCKACRRHWTEGGTLRDVPVGGGRKNSKRAAGGGKAGATASTAASAHVVAPAAAPPTSSSFPDLLRQMLMAPATAGGGGGYSIDLTAWQQMAAFAAPPQAATGDVGGAVGAASTAAPDANCGGGGVQYWNGWLQDDMPGLDGSC

>OsDof27

MEAPLHQSPVPLLPPPPPPPRVVGVQQQQQQEAVVPPPPAMAAAAGGGGREQCPRCASRDTKFCYYNNYNTAQPRHFCRACRRYWTLGGSLRNVPIGGSTRKRPRPSRPARAAVAAAIAAAAAASASGSQIAAQQQQAPPVVMSQHEAAAAAAAASGGGGGDGLLVSLLGAAPVLEGRLGGGIGVDLLGGEQLGFGAMAMPPAPLLWPARVLEGGDAWKSAAAAAGVSYSPFPALWQELAAAAPVELAGGGGLLRHGGGGAPQLM

>OsDof28

MIQELLGGTTMDQLKGASALNHASLPVVLQPIVSNPSPTSSSSTSSRSSAQATQQRSSSATSSPHGQGQGGGAAEQAPLRCPRCNSSNTKFCYYNNYNLTQPRHFCKTCRRYWTKGGALRNVPIGGGCRKPRPMPAPVAKPPMSCKAAPPLGLGGGPVSWASGQQAATAHLMALLNSARGVQGHGGSNVHRLLGLDTMGHLQILPGAPNGAGAGTAASLWPQSAPRPVTPPPPHMDSQLGMGTLGHHDVLSSLGLKLPSSASSSPAASYYSDQLHAVVSNAGRPQAPYDVATASLPCTTAVTSLPSALSSVSAAAPTSNTVGMDLPPVSLAAPEMQYWNGPAAMSVPWPDLPTPNGAFP

>OsDof29

MPPHHGGLMAPRPDMVAAAVAASGGGGGGGGPTGGTAVRPGSMTERARLAKIPQPEPGLKCPRCESTNTKFCYFNNYSLSQPRHFCKTCRRYWTRGGALRNVPVGGGCRRNKRTKSSKSSSSTSAAGSASATGGTSSSTSSTATGGSSSAAAAAAMMPPQAQLPFLASLHHPLGGGDHYSSGASRLGFPGLSSLDPVDYQLGGGAAAAAAIGLEQWRLPQIQQFPFLSRNDAMPPPMSGIYPFDAEAAADAAGFAGQLLAGTKVPGSSGLITQLASVKMEDSNAQSAAMNSSPREFLGLPGNLQFWGGGNGAGPGGNGDGATGGSGAGVAPGGGGSGGGWADLSGFNSSSSGNIL

>OsDof30

MQEAGRRPAPQFAGVDLRRPKGYPAAAQLTPAAEEAAAGVGDPCPRCESRDTKFCYYNNYNTSQPRHFCKSCRRYWTKGGSLRNVPVGGGSRKSSTSSSSAAAAAASSSSSPSSPAKSPKRSKNSKRRRVSPPPPQPVPAPPPPTTADAADVAAPTAPEATTKKAPEDLTAAAATQPAVALGLGVADGGGGGKEHLDTSPFEWPSGCDLGPYWPTGVFADTDPSLFLNLP

>SbDof1

MEAPLHQFPVPPPLPQDALLRQQAVAARALMVAATAGNKAAGREQCPRCASRDTKFCYYNNYNTAQPRHFCRACRRYWTLGGSLRNVPVGGSTRKRPRPRPARHTRAMAAAAFGAAAMATPSTTTTASDGGSPFASPATFQGVGGGGGLLLSSLLLGSVSASSSASPLLALGAAPLLEGRLGFDLGFGDAALGGGGGGGAHAADLPHHHQLPLGGGPPLLPWPAATTRILEGDRAETTTVFPFPPAGAVWQELAATAPVVEAAGLHHGGAPHLLL

>SbDof2

MAECQGGGGGDFLIKLFGKTIPVPESGDAKDLQQSSSSSSSTEQDHQDAHALDQENPHPDSSDPSPQPEVVDAEDPKSSPETTHQKPGQGNGSGDAASQREKLKKPDKVLPCPRCNSMDTKFCYFNNYNVNQPRHFCKNCQRYWTAGGAMRNVPVGAGRRKNKNAVAASHFLHRVGAACGGGGDTLKTTNGTVLSFGGHGGGCVPPGPACLDLVEQLSHHLAAPVIRNAGNNPGPCSEGSSNCRDDNKTINDRSCVDEAAAANGDDGSVQHPASMNNGGATVWPPPYSCAPSPAAYFSSGIAIPIYPAAPGYWGCMVPGAWSLPWPVQQPPSSQSQGPAAGLSSSTSPTTTSAPSVSSSGAADSHTLGLGKHPRDREEGDDGRNAKVWAPKTIRIDDVDEVARSSIWSLIGIKGDKAKQQDDDAAGGHKQKQLVGMVFEPKREATKKPAAMMTSSPLLHANPVALTRSVAFQEGS

>SbDof3

MAERARLARMPQPEPALKCPRCESTNTKFCYYNNYSLSQPRHFCKTCRRYWTRGGSLRNVPVGGGCRRNKRSSKSSSAAAAGSSSSSKTSSSGRLLGGPSATPSTTPGVTGAIITPGLSSFSHHHLPFLGSMHPPGPNLGLAFSAGLPLLGMQHLDTVDQFPVASGGGTTIGASLEQWRVQQQQQRQFPFMTGGILDLPQPPTYQLGLEANRGGGGGSAAAAFTLGQPTTTSATATTGRQEGSPKKMEDSKGQDMSLQRQYMAALRHGSGAHGGWDGNAAGGSGSDGGGTGSAGSTWPMNIIPGFHSSSTTGGNGGGSL

>SbDof4

MAAVVAASGGGSTPGAGGGPTAGGSAIRPGSMTERARLAKIPQPEPGLKCPRCESTNTKFCYFNNYSLSQPRHFCKTCRRYWTRGGALRNVPVGGGCRRNKRTKSSKSNSSSAASASGAGGTSSSTSSTATGGSSAGGASGAGIMPSHQGHGHLPFLASLHHPLAGGDHYSTGASRLGFPGLSSLDPVDYHQFGASAGAGGAALGLEQWRLPQIQHQFPFLSGRPDAVPPTMSGIYPFDVEGHGGDGTGFAGHMLGASKVPGSAGLITQLASVKMEDNPASAAMANSSPREFLGLPGNLQFWGGGSNGGANGNNGGGAGNAGGGGSGGGGGGGGGTVAPGSSWVDLSGFNSSSSGNVL

>SbDof5

MASATTAATGDDAVGTRKGGTGGATAGGGTAPQTQQQTPPPPEQGLRCPRCDSPNTKFCYYNNYSLSQPRHFCKTCRRYWTKGGALRNVPVGGGCRKNKRSRSAAAVAVAAAAAASRLSLNLPPAEAAAADQQQAAARLGFLGAAHHHPAVASSPIGGAGAPAADYHHQQAGMMALQPRLHGPGAVVGQYVPFGDWPSSGDVSSGGGCHAVNGGAAAAVSMSSSIASSIESLSFINQDLHWKLQQQRLATMFLGTPTTSSGAAAGAHVDGAAAAAGAAAPHVVGGTFLHMAAGPPPPHGMVEATTMAPPAATSWFMDSSCYVLPSPTTAHHANTAAAAVVATSNCNVVNSSGGGDDDNATSNNNNCGPGSAIPSWGDMSTFAMLP

>SbDof6

MAGAGGATAVQQPAAGAPAAAGGAARSGVGAGAAGAPVADPRAEALRCPRCDSANTKFCYYNNYSLSQPRHFCKACKRYWTRGGTLRNVPVGGGCRKNKRSRSSGGAGGRNGSSSSASAAAAAVTSSSAASTLSLPPPTGSLPSLTSALGLPGGASLASLLLGTAGSGGDHLGLFQAAMQSVVSSEATAYEMQQQQSQVDHLLGLGYGGATGAGAQIHLKPWMQEAAGAGGIMDSFYAPLLSSSLVPGLEELHVKAEVAGAGDHQQKPAPGDQQSASWELPTPSSSNVDANVIASDALMAAAAASMNPAVSSSTSTAPTTVPSSFMYWGNGGIGGAAAAWPDLANCGSSIATLF

>SbDof7

MQEHGRRPVTPFAGVDLRRPKGYPAPAAVAAAKEAAPARAPVVGDPCPRCGARDTKFCYYNNYNTSQPRHFCKSCRRYWTKGGSLRNVPVGGGTRKSSSSSTSSPSATTSASPGGAAPKNTKRSKNSKRRRVAPAPDPAAPGTDAPTVTATATADVANTAPSTEAAAATVAASEKPVTMTEEEPAAAVVVATETKPPAAPGLGLADAGSGGGGKELLPDPSHFEWPSGCDLGSYWGTSVFADTDPALFLNLP

>SbDof8

MRVTGEKPCTHQELDVDQTNSSSSFNNSSECENLAPSNDEISGSESNLEIAKTEGDVPSGEKVLKKPDKILPCPRCNSMDTKFCYYNNYNIKQPRHFCKSCQRYWTAGGSMRNIPVGAGRRKSKSSSANCRSILIPGSSVATPGGEASLFPLSINGNQAAVSFGPDSPLCNSMASVLKIGGEQIKSSNPASAAQPRNGENQMCPPCTTSSDGPRNESQKETANAHQNGIIGQSNGVTSVHPIPFFPGPPFVYPWSPAWNGIPTMAAAVCPAPAEAANSSENCTTSSNVQWNVPPIVPVLPPGFCGPIPVPVMPPSVWPFITPWPNGAWNAPWLGPSSTVPSSSPTSSSTCSESGSPVLGKHSRDSKPQGDEKAERCLWIPKTLRIDDPVEAAKSSIWTTLGIEPGDRGMFRPFQSKPERQEQISGAARALQANPAALSRSRSFQETT

>SbDof9

MAGQVMEAQAARLQPPTMLAPPFAPLPHSTTCKHDVHHHHLTTTTTTATMAATSGTTTNNVVTRTGGAAAADMAAYLQQLQDAAEAAAKSSGGTGGAARGEQCPRCASHDTKFCYYNNYNTSQPRHFCRACRRYWTLGGSLRNVPIGGSTRKRPRLAHHQHQQHARRAPAAAAAAHVFGLGLGLGGAAPPPMMMMPPLPCSSSSSSLQGQGQGQGQGGGGLLGSLFALGAAGAPPLLEGRGAGSSSSFDFDLGLGLPTAGPLHLGEAAAAVQMQGLGLRGGGGNGNAAGSSSSFLWPAGLLADNDDSVDTWTKMPPGAGAGSMWPDFFSSPPAAAAPQTGGMMLHGGAHLM

>SbDof10

MDQLKSVNDVNAASLPLLLHPVISNPSPTSSSSTSSRSSAQQQQRSTSATSSPQGQGPQQQGQGQGAEQTPLRCPRCNSSNTKFCYYNNYNLTQPRHFCKTCRRYWTKGGALRNVPIGGGCRKPRPMPTPVAKPVISSCKAVGGGVPSLGLGVGLGMGMGAGPGPWASSQQAAAAQLMALLNSARGVHQGGGNMHRLLGLDAMAHLPLHVLPGAGNNNAGGTAPSLWPQSAPRAIPTPPHMDSQLGMGPLGQHDVLSSLGLKLPPPSSSPAAASYYSDQLHAVVSSAAGRGGHEYEAAASGAMSLPCTTALTSLPPAASSVSAALTSCATVGLDLPPVSVPASEMQYWAAGPAAMSVAWPDLPTPNGAFP

>SbDof11

MLSSHHEAMLPYAPGPPPSLLVDRRYKQGGEAAPNCPRCDSPNTKFCYYNNYSLSQPRYFCKGCRRYWTKGGSLRNVPVGGGCRKNRRGKSSLRSAADAAIASGGGRDAAFGHHRFPGPVRPDLVLEGMVGNPSNPAGQAMPGGVPAATDGSTIDLAMLYAKFLNHPPAEEGVNAVTPESAGQVVDEAFDTFSASSDLSPGVLAPLQFDPCHDGFGEWSAGGPVSSTGPSSTASTTAATTMLCADVSVQAAFGELNFAMDQSCFDSLGLPTDDVVGNLSSSWCSIVPGLSTLEDTKYDSLDSFPDDALSLHEDMISGTDHDWSVDCQGLEALYMP

>SbDof12

MAPAAGGFKLFGKVITRTQCAAETAPPAVPTQEQEPAAAASRSTAAFARERDDPDERDQPMVKREAAAAAASDHDFVVVADKQQQQHSAAAGGPAPASESDDSKGQQQHPRPRQHHQQQHQDTVEARAAAAASSAPPLPCPRCRSRNTKFCYFNNYNVNQPRHFCKDCHRYWTAGGALRNVPVGAGRRKNRPLGPVGAVPVPVPVPVPVPAHLHPAAAAGFVLGFPGQHPSSPTTSPSTAVYAERWPVCPDRRF

>SbDof13

MASHLPDADAAGFKLFGKVIQPPDAHHRAADEGGAPPQLPPPTTALPPPPPPPPSPPLPPQPPLPLQQQATGATGGTSGGGGEPLPCPRCGSRETKFCYFNNYNVRQPRHLCRACRRYWTAGGALRRVASASPGRRSPRPTSARSVAAAAAAAAAASSAAAAAAEEVGGER

>SbDof14

MAAPGGGLDQDRGRGRGQDAAIKLFGRSIPVLHSSVVAAAASEVSTKLANDVRSNDGMSCLPNMPLIVKASPFPSKNNMKNGLQAISSQHGKMEADSKSEEVKNSLQAIISQPGKTDDSKSEETKTESGGSGQEKVLKKPDKILPCPRCHSMETKFCYFNNYNVNQPRHFCRNCKRYWTAGGTMRNVPIGSGRRRNKDPSHHHHVTKPCDHIVTANGDVSDATQRQSLAVKPSVLQGSGKQNETACKSVSPVLNIKEQNNADLISLVSGDNKEEKSCASSVVSGSSENWMPENTVKKEEDSTSAYGNGVKEPDPNTQSHHAGPISVFSGNPAAVMVTNQSSADGIHGPGNGTVSPLSLPPPPMVPTPGICAPAVPFPLVPAFVSCIPGWPSAVWGAAWPGSSGPTLLSLPPNSLAFSGSNSRVLGKHTRVANLQEEQKAEKKFWVPKALRIDNPEEAAKSSIWASLGIKPDERIIFKSFQSKDLKNSETKTPESLQANPAAFSRSQTFQERT

>SbDof15

MSDQKDPGIKLFGRVIPLEPEPAPGTTEAEDPPPSHDQPPDELQPRAPELAAAADEDQHNEKEEKPASEMVNMPQEKDKEIKVDTPQVEKDNEMKVDAPQKEHDDEMKIDAQQEKKDEQMEVNGSPMHENIEPANLPPSEHKKEDEDLMNSTEDKAASDPKGENEKTSNEESGQDKALKKPDKILPCPRCNSMDTKFCYYNNYNVNQPRHFCKNCQRYWTAGGTMRNVPVGAGRRKSKNSSLHYRQLLMAPDCMLGSRVDISKSVLPEALVSPPAPIQPTSRNETVLKFGPEVPLCESMVSALNIDEQNVNNSGSAPRGENREDNPGPGTSYNGVPENMVHVDKNGAPVHCNGVAPVPQYYLGTPFMYPWNVGWNNVPVMAPASRFPSPAFPYPLVPPALWGCLSGWPVTTWNIPWIRANGCVSPSSSSNSSCSGNGSPTLGKHSRDSNPMKEEKREKSLWVPKTLRIDDPDEAAKSSIWATLGIKPGDPGTFKPFQSKVESKGQRSDAAQVLQANPAALSRSQTFQESS

>SbDof16

MEEMLMAGNANPNQNPNPPPPAPSAPGAQRAGAPAAGAAAAPSAGATGGPAGAGTERRARPQKEKALNCPRCNSTNTKFCYYNNYSLQQPRYFCKTCRRYWTEGGSLRNVPVGGGSRKNKRSSSAVSSAAAASTSAAMSGTVSVGLPAKNPKLMHEGAHDLNLAFPHHNGRALQPPEFPAFPSLESSSVCNPGAAGMVGNGAAGRGMGALSAMELLRSTGCYVPLQHVQLGMPAEYAAAGFALGEFRMPPPPQSHSVLGFSLDTHGTGGVGGAGGYSAGLQDSAAGRLLFPFEDLKPAVSAAAGGGGASNGADHHQYEHSKDQAAGDGGSGPSGVTGGHETPGFWSNSLIGNGSSNGGGGPW

>SbDof17

DPIKSTREPLYTYPNREKLNETLGVLIKDEAKHKGIVPEEPGMGSSSPSAELIACPRPMHAVAAAAAADRRLRPQHDQPLKCPRCESTHTKFCYYNNYSLSQPRYFCKTCRRYWTKGGSLRNVPVGGGCRKNKRASAKKPPAPPMQPRHMAETGLHLSFSGMPQLPPPSADPLCSLGLLDWKYDPILTGSGGGAAAGSLDGASSEAHFAGAGMLGIPGGGGSGGECHALSALRYAAGLGEHLQLQGLPFGGRAEHDGMEVKPPATERLLSLDWYSETSRAPESAISSLGALGLWSGMLGGAHQHHGSSAAI

>SbDof18

MQEFQSIPGLAGRLFGGAGAGDLRRAQAHAQQGPGARCGGVSPAAPPEVVKCPRCESTNTKFCYYNNYNLSQPRHFCKSCRRYWTKGGVLRNVPVGGGCRKTKRASSSSSATSSVPSTPTSGSGDAAANKNPRRASASSPRSTNSGSASPTAAAATTPTPAPTTPATPSSNSSVAVFTTSSHHHSSPFSTIDVVAPPAPIFADQAAARASLFAPPPPPPLPVFTFAAQPKQEEAPTTTSELQLVAGLSAAAPSSSSVVSEDMAPFASLDAAGIFELGDAASAAAYWNAGSCWTDVVQDPSMYLP

>SbDof19

EAIVSSPIIKEEARSPKQAQVTQQASGSGERKPRPQLAEALRCPRCNSNNTKFCYYNNYSTSQPRYFCKGCRRYWTHGGALRNVPVGGGCRKNKRTSGSISASGTSSSSSAAYAPLSPSTNTSSSKMSINTQLMMVPNMMMSTSSMTGLFPNVLPTLMSATEGGEFNFTMDNQHASLPFTPMSLSNQASVPVLAAGESGTMPSFLEMLRKGLLHGSSSYDTGLAMSDGNNGMDMSFPLPAYGAMHGHGLSGSTTNDARQLVGTQQGVNTGGGFVGSTGVQEEEEEGDNKAMVKSNKNNNGGSLLDRYWIKPNNNNNKRQQG

>SbDof20

MEEMLMAANAGAANPSQGSNNPNPPAPAPGGALRGGGAPAAPLAGAGSTERRARPQKEKALNCPRCNSTNTKFCYYNNYSLQQPRYFCKTCRRYWTEGGSLRNVPVGGGSRKNKRSSSSASASASTSASVTSSSMASAEGAAASKNPKLAHEGAHDLNLAFPHHGGLHAPEFAAFPSLESSNVCNPGGGMTSNGRGGGAGPAVGALSAMELLRSSGCYMPLQMPMQMQGDYTAAEFALGDFRTPPPPPSQSVLGFSLDAHGPGSGAAAAGYGSSAGLQGVTENAGRLLFPFEDLKPPVSSGGGGVAGGATGGAGDGNSNHNQFDHNKEQDGGGGPGAGHDTPGFWSGMIGGSGASW

>SbDof21

MAPAASILSATAAAAGASKRPADSDAELSLDSSALQQQGDEAVRKGRQTRQQQQLECPRCRSTNTKFCYYNNYSTAQPRHFCRACRRYWTHGGTLRDVPVGGASRRSSGGGGKRRRVSAEPSSAASSSPPPLPAASLADACLPDLTSAFPFLSDGSFFPQFDVGSGVALAPAAFSSSWQSVVPDFYDGLAPWDDGAAAGTAAGGFVGAWGDIAGLDLSWTPPGN

>SbDof22

MDGVSTSPSSVTATATIREKPKPAPAATAPASLYLAVLRPSGSPSPSLSLYATGPRASQPEPGTTAPSRMQEVSVEPGRRPAQQPHHQFAGVDLRRPKGYATAPVQEATPAVKVAEGGDPCPRCASRDTKFCYYNNYNTSQPRHFCKGCRRYWTKGGTLRNVPVGGGTRKKPSSSSYAAAAAAAADADNKPPKKKPASKKRRVEAPVPEPAAAADASGVTDTVAAADSAKTTTTTTTTTTDGASEITTETEAAVAVAVPVAEEDSESLAHLLLQPGAEEAVSLGLGLSDFPSAAGKAVLDDDSFVWPAAFDLGTCWASAGFADTDPASLFLNLP

>SbDof23

MGEGRAGDGLIKLFGKTIPVPETPAAGDAAKDIQQSGSSGTTDLKGQENTLQDSTGSPPQQEVADTEDSSAAKNSSADKQQGEAANQKEKLKKPDKILPCPRCNSMDTKFCYYNNYNINQPRHFCKNCQRYWTAGGAMRNVPVGAGRRKSKSASAASHFLQRVRAALPMDPLCTAAKTNGTVLSFGSDMSSLDLTEQMKHLKEKLIPIAGIKNGDERSVGSCTEGPAKAEDSNQKENVTAEKSAKLVQHPCMNGVAMWPFSCAPPPACYTPGSIAIPFYPAPAAYWGCMVPGAWNAPWPPQSPSETGSTLSTASPASTKSNCFTPGKRPRDCNEEGDTKGNGKVWVPKTIRIDDVDEVARSSILSLIGINGDKAGKDGKGCKLARVFEQKEEARTATHSVINGLPFLQGNPAALSRSLTFQEGS

>SbDof24

MVSSPNIKEEARSPKQAEATQQPSGSGERKPRPQLAEALRCPRCNSNNTKFCYYNNYNTMQPRYFCKGCRRYWTHGGTLRNVPVGGGCRKNKRASRSVSGSGSSSSSAAYAPLSPDTNTSSSKMSINTQPMMVPNMMMPTPTMTGLFPNVLPTLMSTGGGSHFNFTMDNQHASMPFTPMPLSNQASVPMLAAGGSGTMPSFLEMLRRGLLHGSSSYDAGLVMSGGNNEMDMSFPLPAYGAMHGHGLSGSTTDDARQLVGTQQGMNTDSGFAGSTRVQEEEEEKGDNKAMVKSSNNNNAQQQQQQQQQQQQQQQQQQQQGAAGVVCQAVEVLLLQTI

>SbDof25

MKLSSSSHHLLLESSNLTLSPHHRNTAVVTRMLSSHCENMLPYAPGRRAAVLLDHRRYRPNVVEVAPSCPRCDSPNTKFCYYNNYSLSQPRYFCKGCRRYWTKGGSLRNVPVGGGCRKNRRGKPVVRAVAVDAAVAAASGGASALANRSSSSSPATLRPDLLLEGMIGSPVGLCQPTDDDAAEIPAVVAPEGSTIDLALLYAKFLNHQPAAAAEPCAAAVVPESLDTLSGSSTSGDVSPVVVPPRDQHQPFTTQDHGGFGELSATTASAEPSAAPPQRPADDACACAAEALIGALSVDPRCYDSLGLPPDGGDLVLPSTWHLGTKYEPFDPLPEDAMSLQDGFAGDEDVWSSALACQGLEAALCRP

>SbDof26

MGGPLPDGGGGGGGGAGGQVVGGPAKPMSMAERARLARIPLPEPGLKCPRCDSTNTKFCYFNNYSLSQPRHFCRACRRYWTRGGALRNVPVGGGYRRHAKRAKPKQQQAAGAGAGGGSAGTGNTATANAALQHAPAGSTASSAAACTATTTNALPGGMLGGGGLSMLPPLLRLADFDAMSLGSTFSGISSMGKPGSIDAYSHSVGGGGAPAGLEQWRVQQMQSFPFLHAMDQGPLGPPLAMAMAAPGMFQLGLDTTSSDNGHGRGGGGGEDGSTGGELHVMHQQAATKRESYPAPRGMYGDHHHHLAAAGGYTSAYSTNAAT

>SbDof27

MEAGQVPDGRALMAAVTTTGGGGREPEGLPCPRCESVNTKFCYYNNYNLSQPRYFCKTCRRYWTRGGALRNVPVGGNTRKATPATGRRKRSTPAPVNVTVPAPATASPPPPPALHGGSLLRPYGGGGGSGLLSFAAPALASPLAAADPDRRLLDFGGSFTSLIAPGVADVGVHFSAGFLMGGLAPAALPRAPGSVAALPPPPPQQQPTVSQALPEGMVWSMGWPDLSI

>SbDof28

MQMQQQPPLQCLLGGGGGGSDHHHLMPPPSGLAPLPGGPADTAASAPAGGGSSTSMQAAAGAGTAAAQPRPVVSMAERARLARVPLPEPGTLRCPRCDSTNTKFCYFNNYSLSQPRHFCKACRRYWTRGGALRNVPVGGGCRRNTKRSSKKSSRGGGGAGATAATSSSSTTSTSTTATTTTATTTSAAMAAAEAIASMQAQLPHLGLPPAAAAAALEASLEGYHHYLPLQMQPQFLQQAGLHGYHFADDGTGVLADGFPRGVVASGLLAQLAAVKMEEHSSNGGGAVAAHHEQSYWPGSTGGGSGWPAEFLSGFSSSSSGNVL

>SlDof1

MESTQWSTHEEIGVVKSSMGAEIMNKKVRPVKDGAINCPRCNSINTKFCYYNNYSLTQPRYFCKTCRRYWTEGGTLRNVPVGGGSRKNKRLSSSSSSSQKLPDLNPNPTSHHQNPNNIVIGSNQDLTLGFRTVPQDHHTSFHGVIPQFLEFPKMDGSNNHLGSRTGIASRGFTSFISSASTPDLNALYNSGFPFQEIKPSAGNADHAASLSNSNYSSGGPGGLENGSGARIMFPLGGLKQLSSTNQVDHLTKGQENNNSTAGLYWSGMISGTGGSW

>SlDof2

MTLESSEKLVTKQQTGGVQAPPTQEPDHHLPCPRCDSINTKFCYYNNYNLSQPRHFCKSCRRYWTQGGTLRDIPIGGGSRKNAKRSRIYTNTPFSSTIASVSSHAVPGNSPFMLPLPAANQLLFGTDVKPINNFTSLLSSHGPGVLALGGIEDMGFGIGRGNVWPFTGAPDSYSRNYNNGGGAGMWQFSGGEGGFVGSGDYFN

>SlDof3

MTDPAIKLFGRTIQLPDIPDSSGAQGDDSLPGDNNGEEDEEADKDDFGGNLDDDEEEMEILTGKELQDQNSEPTKTDSIKELPVDNDCSTRPSKSEEEQGEASNSQEKILKKPDKIIPCPRCNSMETKFCYFNNYNVNQPRHFCKSCQRYWTAGGTMRNVPVGAGRRKNKNSIPHYRQISVSETLSNAQTDYPNGIQQPILAFGSPTPLCESMASVLNIADKTMHNCSQNGFHKPQEPGVPVSYVVGDNGDDHSRRSSVTSANSEDEVNKTVPDLLKKNCHNFPPYMTYYPGAPWPYPCSPVPWNSAIPPPGYCPPGFPMPFYPAASYWGYTVAGSWNVPWMSPATVSLIQTPTTSGPISPALGKHSRDENIQKPLSSMEEPSNESNPEKCLWVPKTLRIDDPGEAAKSSIWATLGIKHDTVDSVGGSPFSAFQPKNDDNNRVSENSTVLQANPAALSRSVNFNESL

>SlDof4

MAEVQESPISQGIKLFGATIEIQEKQAKATHQPTNKVVVDDDDDNDQEKRPDKIIPCPRCKSMETKFCYFNNYNVNQPRHFCKGCQRYWTAGGALRNVPVGAGRRKAKPPCGPGPHGDLADGCNLFDVANQLDFDGSVVAHEDQWHLFPAAKRRRSTSDSQSY

>SlDof5

MDSNGTNAATSNNMEKPIQDPSQQQQQQPPPHLKCPRCDSSNTKFCYYNNYSLSQPRHFCKACKRYWTRGGTLRNVPVGGGCRKNKRIKRPSVNSSSSTTTTSHDIITTSTPNIVNPSHHHQLHHGVVHNNIDHLSTTNSQNHLNPLFYGLTHERSDLNIPFARLFNSRVSSHAGGVDPEGQVYSLTDNIPGLMDRRMGLGFSNSSGGVVNMGHENNNNNNNSSSSNYGHGGFNPIKQIQDVHVMSTSNCTTSTTSLLSSYPNMFGSSSSTSTMASLIASSLQQQKFMSNINGNSFHSLLTPNNYEELQMSRGENNNTNINNVHEGGGNGITMLKEEKMDLSNHQIHEQIINSSDPSLSWNGAWLDPSNMGSNSVPSLI

>SlDof6

MEDQLGGRSSGENDRNQQQRRMKMPENNSASPAQPPPQKCPRCDSNNTKFCYYNNYSLTQPRYFCKTCRRYWTQGGTLRNVPVGGGCRKGKRTMKGGGSSSSAGESSSSRSHHQVLYPPQIPNLSAAAAAFFSGNNSRSQPPPLPSMSSLYTGAAGGGGFLSSLAAMQSMSQLSQGINNQSQLGVISGTNNNNNQFGNFNIPTPPPKVQIDQQMESTGFYQKPNLESSFFPSDQTLQFQPARPLGSWTQRFINNNNNNIWPNNSASNTSSGAASSSAANASLGPNDHQWPDLPGFGPSP

>SlDof7

MGLSTKLVSIDDDGLDNWTTTTHNTRPEPPSIRRQLPSKSESLKCPRCDSINTKFCYYNNYNKSQPRHYCKGCKRHWTEGGTLRNVPVGGGRKNKRVRPTDPVDHINGRKHVRLEVNDQRCPLITTTSMTNSIITSSILPSVTLIRGNSTITSAIDEDIKNLTSSSLPYDIFSNISQDHGNTHFSLIPNSSTNTQLSSNVYYNYEHMGKFDSTILEESTITTIMPITSNNDLHSYEPWKVPETSNNDLIIDENMSNNYWNWNEFETLSNAADLNISWDDLEIKP

>SlDof8

MSEAIAIKDPAIKLFGWTIQLPDFPAPAPEDSSFLAGEVEQELKGLYDDCIDDNEHLTTEDSQDQNPIQQRCDIINYYESSTAKTSKSKEEHGETSNSHERNLKKPEKTLPCPRCNSMETKFCYFNNYNASQPRHFCKNCQRYWTAGGTMRNVPVGAGRRKHKNSVLHYSHVSVSEALSNVRTNFPTETQHPPLTLNGTILSFDTDKPVSESMVSVLNVADKGMQNCSGNGFQKYKELRIQAGDNGDDHSDGSSVTAISSKDSDNGLPNTPRKNYNSFPTHLPCFTGAPWPYIWSSVHCRNAVPPPGYSLPGIPMSFIPATTYWGCTIPGSWNVPWMSPPTASHNQMPLTPDPNSPTSRKHSRDENVLKSTGTEEEQRKESDPGKRLWFPKTLRIDDPGEAAKSPIWATLGIKHEVVNSVGGGLLSDFLPKNDERSCVSENSTLLQVNPAAMSRSLNFNESS

>SlDof9

MPSDVRATKQQQGGAPAPEPEHLPCPRCDSTNTKFCYYNNYNFSQPRHFCKSCRRYWTHGGTLRDIPIGGGSRKNAKRSRTITTNSMNSSCLSSTLSPRDYHHAPHPSHVSPFLVPLTADHGGSLPFDVKPSVNMCGSFTSLLSSAQGPGGLLALGGFGLGVEDMGFGLGRPIWPFPGVSHNTSVDNNSNGAGASMYGSTWQLASGGEGGFVGAGGEIFNFPDLAISTHGNGMK

>SlDof10

MIQELFAGNTTLIGDDNISNITPSSSPISCTTSNSNIAPASANANSENLRCPRCDSPNTKFCYYNNYNLTQPRHFCKTCRRYWTKGGALRNVPIGGGCRKNKSIATSKSTAAKFKNSLPFEFIGKSGIFGGFEQEIIPSNYDNNNPFLFSSPHQNHNPILSLLKGNLHKSIGVNQFPSNNGIWKNNYEENVGEVQNSRGFQELYQRLKASTNRCYTDNMHGPSSSSMILDSAPVTGGELGCWNPTLSTWLDLPTANGAYL

>SlDof11

MDTSPQWPQGIGIVKGVDEAKLDQRKPRPQKEQAVNCPRCNSTNTKFCYYNNYSLTQPRYFCKTCRRYWTEGGSLRNVPVGGGSRKNKRSNSISISSSSTTSSLSLSSSSKKLFTDLANPNDLNLTYNPIPSGGTTTTTATNFSNFSDFMALPLLHPSANSASTFMTSSNLYPSSTTGISNLHDLKSSNGINFSLDGFENGYGSLPSHQEAKLFFPMDDLKINVSTVGDEQFEENRGQAADQSNGFWNGMLGGGGTSW

>SlDof12

MGITSLQVCMDSSDWLQGTIHEDCAGGMDSSSSPSGGDHINNLMTCSRPIIVDQRRLRPPHDHSIKCPRCDSTHTKFCYYNNYSLTQPRYFCKTCRRYWTKGGTLRNIPVGGGCRKNKKVSSKKSSTNTNESIPLSTTTTTNNNNIPEMPFPHHFMSSTNFGHHGNFMLENQAPIIDFMESKYEALVGSSSSTTTNSRLLNQDLFLGNGDNNNNIGMMMMSGSTSTNGFGHDHDNIVATNYPFGITSIMDSSNNGNSFGMLLPYENHHEEVQNINAVEMKPNPKILSLEWHDDQLGSNKESSFGYYSGNGGLGSWTGLMNGNCYGSSATNPLV

>SlDof13

MREVKEPEIKLFGKKIVLPENGMILPVIVTGEDSDVGKSMSASEVVTADESSTGSDRDPCLVDKEGNSSQQDESDDGSEYEKDEADKDRMTRELSEAKLEEKDQNLMMEESENLKSPSENKTKTHTIDDDSPTVKSSKTEDDQNDASNSQQKTLKKPDKILPCPRCNSMDTKFCYYNNYNVNQPRHFCKSCQRYWTAGGTMRNVPVGAGRRKNKNSASHCRHIMISEALEAARIDPPNGFSHPVFKPNGTVLSFGPDLPLCDSMASVLNLAENKTPNGIRNGLYRPENPSGIGGENGDDCSSGSSVTTSNSMAEGVKNCPPEAVMQTINAFPSPVPCLPGVPWPIPYAAVPFPAISPAGYPMPFCPPPYWNCNVPGPWSLPWLTPPSPTANQKGSVSAPNSPLGKHSRDGELLKPNNPEGQKNSEGSVLVPKTLRIDDPDEAAKSSIWSTLGIKYDSVSRGGLFKSLQPKNSEKDHPTTTSPSLQANPAAFSRSLSFQERV

>SlDof14

MQDPSIYSQIKPQFPEQEHLKCPRCDSPNTKFCYYNNYNLSQPRHYCKSCRRYWTKGGTLRNIPVGGGSRKSTKRSSSSSSSSKKSSSTSTTSPATPPLTSSSSSTNPKPEPFGIPAIPSFDVMTTSTGPFSSLLASTEPQFGNFLEALNPNNNNGSTLQLGNPVSSSGHQNGNTSYLGVQNGGESNNCWNGGNNGWPDLAIFTPGSNFQ

>SlDof15

MLPYHPRPMIMMERTRKSNIEQAPNCPRCASTNTKFCYYNNYSLSQPRYFCKACRRYWTKGGSLRNVPVGGGCRKSRRSRSTRKDDNTLQTSSPALEGAPGAHDIDLADVFAKYLNQGTNNDHDDNIIIQESQDYSSIGASLSNSPSSDSLVNNPTSFENESLLDNFQDYPCGNFLQEEQGAQINQDFLDFNASFLEMQAILGQEEDQFDHYNTSNFEWQPMMQFQDFGSILELDDHQLTKNSTTNLASHDNNNNNNYSSFDLSNYI

>SlDof16

MSSEIGDRRPARLPAPVNGTRPSEPENLPCPRCDSTNTKFCYYNNYNLSQPRHFCKSCRRYWTRGGTLRNVPVGGGTRKNSSHKRPRINSGAGTVQEQTNPITMMGSGSGHVSGSGSMSLMGCEVNLNESVHEGGNGTSSFTSLLTAPVGVGVGGFVPLGGFGLGLGGFGLGNLDWPMEQVSGGGNGGDGGENDKWQLSGGEMEGGGGGGGSGGGGIGGDDDCFGWPDLAISAPGTSLK

>SlDof17

MSEVRDPSIKLFGKTIGMTQQETNCVYLHDDHTTSSPLSIDDEKINLEGEVTQSKQVDELVDPAADSSIEPETSSGISDDIKMQDADKETLSSKSVEEEDSSEEKALKKPDKLIPCPRCNSMETKFCYYNNYNVNQPRYFCKNCQRYWTAGGTMRNVPVGSGRRKNKSSSISNYPLQAGRVEAAAHGMHLPASRTNGTVLTFGSDKPLCDSMVSALNLAENSHNMHRNEYHGSEQRMPTIGNDQSNGSCSTASSVTDKESSAGTHDLANWSNFQPFPPQVPYFQGAPWPYSGFPVSFYPAAPYWGCTVSNPWNVPWLSSNQSVHNNSPTSPTLGKHSRDESKLDPSQSRRRDTTLQDREGERCVLIPKTLRIHDPNEAAKSSIWSTLGIRNEKIDSTRGTMLFSAFNPKADHRNRELDTSFALQANPAALSRSLHFRESTR

>SlDof18

MAFSSIPFYQDPPNWHHEQGNHHQQQQHLGITNENSSELSPTVLPPPGAAPGGGGGPVGSTRSGSITERARLPKITQPDVALKCPRCESTNTKFCYFNNYNLSQPRHFCKTCRRYWTIGGTLRNVPVGGGCRRNNKRSSSKRSRKSPIRSERSRNVPISTSNSTNTITTPSHFPPSSTHLSFFNTPFHNFNNFNSTQNCLNFGEIQPHEGDPTFVDQFRHQQMEKFSFFSPLEQPSNLYPIYSEFRINHHDLENVKVEENKSSTNSTQGMNLQRNNNLGVNQFWTNYNISSTSTSQLL

>SlDof19

MDSSNAQNYHQEMSSQTLESMLACTKPQQEKKPRPQDEVQKCPRCDSSNTKFCYYNNYSLTQPRYFCKSCRRYWTKGGTLRNVPVGGGCRKKQRSSSKRSSPDNQSLMTTTTYPNNQISPLTQFSYDPNDLSLAFSRLQNQESGQLVCPKLQRTVRTESIFSDSLYLTNFGNSVCHDVFENRDMCTSSSEAFSRAGFLDTLSDGLLDASNGFLHHNLYYYGSNGNNGNNMGHVESASGEMITNFDQEVRSGALKEEIMCSDNKILLGYPWQINGDGNVADFEYSNRQNWNGLGVPSGHGLLNSPLM

>SlDof20

MQDPSSIYSQINPQFPDQQVLKCPRCDSINTKFCYYNNYNLSQPRHFCKNCKRYWTKGGILRNIPVGGSSRKNTKRSSSNSCKRSSTMTISSSTSSEQNSKTEHFDTPVVRNSPIVDANGPFGSLLASNGPEIGNFLNVLNPNGPDSGSDAAAAQSGNSNNNHEFLGEDSNCWNGTNGWADLAIYTPGSTFQ

>SlDof21

MQDIHPIGGGGGRFLGGAGDRRLRPNNHQNHQALKCPRCDSLNTKFCYYNNYNLSQPRHFCKSCRRYWTKGGVLRNVPVGGGCRKSKRSKPKSTTADDTPEEPKSDTNSSSESSSLTATTTAAAAATANTPGAATTEDVSATSSNSASTYLNFPDSNFFIPHSTNQTFDDQPLMENSVEDQFQDIGNFTNMMTSSNDPFNMVDIPAYRLPENQNSNEQWNTETKMVETLPTSGEMKMEQMSTDFLNQTGRVDEYPGLHQSNSELTPLNWQTGGDHGLYDLTGTVDHQSYWSQTQWGENDNSLNFLP

>SlDof22

MTCDSEIKLFGKILPVVVSGVGRGLSGSDGVIYDGNRNGSDLDRCLEGSKASSVEKDEGSEYEKQEAEKDNITGELSEAKSEEGDQNQMIEESENPKTPSESESSPKSSTEEDPQAVKSSKTENEPTNVTNSEQNNLKKPDKILPCPRCNSLDTKFCYYNNNNVNQPRHFCRSCQRYWTAGGTMRNLPVGAGRRKNKNLASQYRNISIPEGLLAAGIESPNGLIHHPLFKPNGTILSFGPDLPLCEPMASALNQAEKRVSTGIQNGSHKSEVKNSSCKGGDSGDECCRGINIPTPNMMVEEGKGEPHKAVMHSINGIPSPVPCLHGVPWPFPWNAAVPVSAICPIPFPMPFFPTPYWNCSVPPWSNPWLSPPLRAANEKTSGSDPTSSLGKHSREGDLLKPSNPGGKEQSEQKYSEGSILVPKTLRIDDPDEAAKSSIWSTLGIKYDSTNRGEFFKALQPKSNDKHNKANTFPVLHTNPAALSRSITFQQGA

>SlDof23

MCFYCQDTIPEENEFDSSSSPSGGDIFTCSRPLMERKLRPQHDKPINCPRCDSTHTKFCYYNNYSLSQPRYFCKSCRRYWTKGGTLRNIPVGGGCRKNKKVSSKNSKLPNDNITTPHVESSNNYPEMSFSHFGNFMGNNNMNHNFMHHAPIDFMDSSKYQALVGTTSTRNQDFFGNVNVGTTGLINGYGEMDISRIGPHYCSSAFGLPNMDGNIINYEGQNITMDVKPNPKILSLEWHDQGYFNGGLGTWSGLMNNGYGSTATTNSLV

>SlDof24

MDPSSAQHQHHQELSSQTLESMLVSTKPQQQDPKKPKPPEQAINCPRCDSSNTKFCYYNNYSLSQPRYFCKSCRRYWTKGGTLRNVPVGGGCRKNKRSSSSRSSSISSQDQHSIVNTPNNPFPYDSSDLSLAFARLQKQGNGQLGFENHGNLSMMCNENPSGIFLDALRGNTGFLENNNPMNGLIHQQNLYYGVGNIINGDIGLHNVENGGLGVNNNDQEVGLMHNYDQEISSGVTTSTTMTTVKQEMCNMAKDQGDHNRVLWGFPWQINGEGINMADFDSTRRMWNGVGGSSWHGLLNSPLM

>SlDof25

MVFSSFPVYLDHPNLHQLQQADGHQQGNTGLELPTVQPPPPMQVGASPGSIRPGSMVDRARLAKIPLPEAGLKCPRCDSTNTKFCYFNNYNLSQPRHFCKTCRRYWTRGGALRSVPVGGGCRRNKRSKSSSNNNNNNSSKSTGGSNVNTNKTIASGTSTSASPSSCSTEIMNGRHHFPHEQPTQLTPLMAAFQNLNHHYGGFQPNLVSTHHGNGSALSSHHHEMGFQIGNSTNTNNTNNLPVPSGGGSDHQWRLPSLAANTNLYPFQHGSDQGIHESSAGNNNNINGHHDEQGLNSTKQFLGTMENNSNQYWGGNAWTGGFSGLNSSSSASHLL

>SlDof26

MEEMKIPKNTNTRKIRPQQQQNDEALKNCPRCNSTNTKFCYYNNYSLSQPRFFCKNCRRYWTDGGSLRNIPIGGVSRKSKKSSSINIMKNNIISPKVQDIINNNNNKGVNQDLNLDFSSDFKIISELIQVPNNNSFMPIMPNISDPNSIYLFSSNLDHGLIGSSSISDGGYECNNIIQDLQVCTSTTTSGGILFPFEDLKQVSNTSEQSRDGESSTNGYWDVILGGN

>SlDof27

MDTSQWPQEIVMKPMEDIIGNTSKPTNCVERKLVRPQKDQVVNCPRCNSTNTKFCYYNNYSLSQPRYFCKTCRRYWTEGGSLRNIPVGGGSRKNKKSSSPYNHHVIVNNKKLPDLVVPPPQPDNIEEYPERHFGNRPHDQNPSKIILEGSQDLNLGFSSDFKTITDLIQVPNYDGSNKDNNNISTILPPPSSSSSASSPSQLSVMELINGITNNNNNNNNNSFMTMPNSVYNSSGFSLMPSLNFSLDHGIGNDVHHSSSYGNNNNNLQDTNANGRFLFPFVGLKQVSNNTSDGGANEPSLGDQSTNGYWNGMLGGGSW

>SlDof28

MADRARMANITMPETALKCPRCDSTNTKFCYFNNYSLSQPRHFCKACKRYWTRGGALRSVPVGGGCRRNKRSNNNNKNSTNNNNNNNSSKSPASSTSTDGRQGTNNSGSSTTISSHSNSFSGPTSAASLLGLMSPQIPPLRFMSPLGQFSSDHHHHHHFTPSNHMNLNFSTSSCGNILGGTTEGMMVSNNNLLGTGTGAGVGGHVASLLSSGNLEHWRMQQQFPNFLGGFDPSNSPSSYPFQGGVHEAVQYLGGESTSQISRPKISTSMLNQMASVKMEDNNNNNSNQDQSALSRQLLGIQGNNENWNTSASAWSDLSASFSSSSTSNAL

>SlDof29

MATQIAPPNVTCSRTSTTMEKKVRPQKDQVVNCPRCNSTNTKFCYYNNYSLTQPRYFCKTCRRYWTEGGTLRNVPVGGGSRKNRRCSISSSSLSSISSSQKLLDLNPNPSLSSLQNPNYNLNLGSNQDLNLGFPSFNIHNHNNYFRGMPQFLDFPKMDKGNNGINHFSTSTSNTSPVSALDLLQKGIASRGLTASISSSSSPSTPDLNALYTSSEVQENGAKMMLPFGCLNNHNNSESKGQENSSSVGFWNDGMLGGGGTW

>SlDof30

MVFSSISAYLDPSNWQQQVGYSIPNPQLPSGLSQPTPPRPLASTPPPPPPPPPPPQPHHVGGGGSIRPGSMADRARLANIPMSEATQKCPRCESTNTKFCYFNNYSLSQPRHFCKTCRRYWTRGGALRSVPVGGGCRRNKRSSTSNSSTSAKSSNNNTKSQGSSQTTNSGSTSNNNSSPSSAASLLGLMNPPIHPLRFMSPLGPLTDQHFTPNEMNYTSISSPSPAPIVMGTNENMNFQLGMGSNLEQWRLHQQLVNQFPYNLYGGLDSSPASGSGSASASGLYPFHQAHYDASGGGVISQIRPKVSNPMLTQLALMKMEDDQDHVASMPRQFLGNENWTSNANWNELSASFSSSSTSNNVL

>SlDof31

MSQDNKGESQSSGGGDDGGGGPMGARPKEQALNCPRCDSPNTKFCYYNNYSLSQPRHFCKTCRRYWTKGGALRNVPIGGGCRKNKKMKTSSSSTRFSGDSKDTISGSSDIGGLKFFHGLSPAMDFQLGGLNFPRLSNNSSTSTVGGNIFNQFSSFGENSTPNIGSTSCFSLDPSGSSSLLGFNNNFPFSSSMLKQGNEGVQEMGSMGVHHGTMASSIESLSSINQDLHWKLQQQRLSMLFGGENQKENIISSSIPLHDDQNQNQNQIQIQKPQPILFQNLEISSSKQQEDHQETFGNNNVDSRKDCSTTIGNHHGNNLSTEWFFDNSFGLNSNSTHSNNNNNNGNGNANDDQNVNNWNSTIQAWSNLNQYSTLP

>SlDof32

MVNRARIAKLPLPEAGLKCPRCDSSNTKFCYFNNYSLSQPRHFCKNCRRYWTRGGALRNVPVGGGCRRNKRNKSSSNNNSAKSGGGGGLMGNSNNASTSGIPSINCSMEMIGHHFSQSSTQFTSLMGAFQNLNNYGGGLIQPPTHQLVGEMGFQIGSNNLLPSLVASNNFEHPTNLYPNFQNEGTTTTTTTTIEASNGVTQQVKMEDNNRQGMNSSTKQFLGTLENNNQYWDVANANANANVNSWIGFSSDLNNNSLSTTNHLL

>SlDof33

MDHHQQEMTSQTLESMLVCAKPDQDQKKPRPAAAEQQPQKCPRCDSANTKFCYYNNYSLTQPRYFCKSCRRYWTKGGTLRNVPVGGGCRKNRKLSSAKRSSQDNISPNSSNSSTDLSLAFARLQKQTNAIDQEQDTNNNMSMMYNTNNDNTSTTFLDALRGGFLENHHGLFQHNMYNYANMGQLVENGEMGLSYDQDQMSIGTMMTTTMKQEMCNVARSTEGHDLNDNNKVLWGFPWQQMSGDHHVNNNMNTNDFEYSTNKQSWNGFGGSSNWHGLINSPLM

>SlDof34

MVFSSIQAYLDSSNWQQAPPSNYNHDGTGASANGGHVLRPQLQPQQQPHPNGSGGGGGGGGGSIRAGSMVDRARQANVALPEAALKCPRCESTNTKFCYFNNYSLTQPRHFCKTCRRYWTRGGALRNVPVGGGCRRGASQFSSVKVEDNPYKWVNVNGNCSSWTDLSAFGSSR

>CrDof

MVDGGSRAASGQLDDWAAGVAADLDQGEGDRAGARRRPARDASPAPDARKVTTFTNKKRPASDRDSSPEEDDEEQAQKGSLKADGTRPKLPRPDKKEACPRCNSMDTKFCYYNNYNIKQPRFYCKTCQRYWTAGGTLRNIAPGSGRRKSKSKAAREKNSPSLAEQLTAVAAGQGMFGLGGGGGYNGISPALALAAATDPTGLLAANSAAAYGLGGHGTISGLKLGGVGGLPAQFNSELALREHLAGQHSLETRLLLNGHLSAEDLPNGMSAAALAQASAQLHALHGQGSGIAQSLAAGNGHTGSPSPSPPPAGNGGQQHPLSSSPQHGGGSQASQQPSPPQQGSDDAEGGGEERYVAQGRRVRVKAELDGNAVSSSLAMGGGGGSGAYANGASIASSIANAQLAASLSMPPSMGALAAVMGPGGGPSGLHPLLAQDNGGSLLDAGLTRQQLLVLQQHQAMQQAQQQESLQQLSSLQQLQGLAALHGQHSAAGLAGLDPLQRSALLHSAAGLGGVGVGGWLQGGGGGNSLAAAAALESLQAQHLLQAQQVHPSAAAALIGGGGSSAAAQMLQAQAAAAAAGGGGGWQGVASAANWPSAWSSYSGPSSGSYAGYALQAAAAYSGAR

> PpDof1

MSKLRKWMMKEERENSAGSADTSAIKLFGKTIAVNSGVSDSQETSTKAGTSADAAVVHPCASQAGSEEVDGASTQSEADEMGSSICNEIRNVEAPKDAPQESQVSSEKLASETMTVSTSIQAPAAAVAQEKQRPLTVEEKKEQCVRKPDKLIPCPRCDSLDTKFCYYNNYNINQPRHFCKSCQRYWTAGGTLRNVLVGAGRRKNKYGSSQMKQEASEGVNSGITTVRLDHHEGAQPMLPCAINPYGLKVPPMNIPQLAASCASRQASEAQSTVTSCGKESNKVFTSASAFKPAGPDVSNDSVSKLRQRTRTDTPEGKDVSSPGSSPFVSESPTSHVLTSRVQPQQQEVQQQAMWTKSNPQLGFFNGAWPYGYSIGWSGAGPIHGTPAMCSPHPASSGTTSPPSGMCPTPGNVWTGLWASGMPGMPQGMASMSGMSGVSAMPFGWNMCPGGWTLPWRPVPAVAVQGSADVVVVNSGGFRKRDGANDEGVRKMARIGSPEEVVV

> PpDof2

MNRNKGQQPSLISKDAEGQTTGLELNASEVKTHLSCENPITNDVEDDDDGLSGAADKLQGDVAGVHEIVVTPFPLSSEKQESETEPLDSIHLKSNSGDVVADGSSLELANELQNEVPGVSGLDQSAGTEALGPPQHQTAKLEEGTVGRRPLAVADNEDTVGSVRPPPVDGDLVGPSPKQAKVQHQKPALKKPDKLAPCPRCDSLDTKFCYYNNYNANQPRHFCKNCQRYWTAGGTLRNVPVGAGRRKTKHGNSHVRHGGLLDIPAARQDSDMALSRPCLSRGASVPDQVSPLPASSSSSPSTITSLPSSQDRSSSDAVTQTCSMRPAEAVRKHHIPEPQVNVSGDSGNRLLKAEHGGGCEPVLAGEAENCAEACTSSLPGSPQVDAALASQEVQSMLRGPPTTLIPVCGGIVRPVGILDG

SVWTAAPTPFGLFNGSWPYGYNVGWSGPAPGAPGAPTCPPGGTLVSCQPAVAWSAPPNGLWSNVCWGSTLIPGMPAGLPGPSWMSPGWGAGWPMPWGPDAAAAAAAAASASMQVPAVPMSGNVSPVQSRLPIRPVLNKHPRDRPELERVESSLWVPKMVKIDDAVNTPRSSVWTAVGAENRVETVASGSNFKAFLPVGDNKVSITSGDRKQVKS

> PpDof3

MRRVAVEPPEFVHQIKTCTKCGSVNKCKFRYLNNGKQNQPRYQCWVCNDFFSHVLHGRPKGKKYLKPRGVDPKELQGVVKKCTKCKVLNKAKFMYYNNKNLAQPRYQCLVCLQEFQVHLNVLLQSGGSKQGDTPNAINMLPQSGGSPQDGSPNDVSRMLEVVAALANSFRAQQCGNFSQTDELGNPRQPVDTSGNPTQPADESENPEASELDMLLQEDDLENPMQQASEFDILLQEDDLENPMQAVCIDVLLPTPTLAPADGFYNLLPTDGFDDSKQIDDEFNNFLQSMGFDL

> PpDof4

MESPSTSRRPTEARPRNREAQEPEEFANRSKACQRCGSTDVKFKYLNNKKQPRYECRTCRNGVRGSSLFNPFPMRPLHRKGYLKIPALQTMEFMVQVKVCPRCKSSTHVRFLDHNNKSPKHPQYRFREWKLDFSPFSPFKLRVTGSQKGHE

> PpDof5

MADSGLGSGEGDSPVQSKGNGGAIREKPVKVLPCPRCQSMNTKFCYYNNYNVNQPRHFCRNCQRYWTVGGTLRNVPVGGGSRKKLRMARSRTDPYLRAGEAPQTGPLSSMSGADGLLGASTCVQQLPMLPSTPGLVCFGTQLSGFAPAGSQLPFVQTALEQSQSMYTRVQQKPLAGLQEVPDLQSVYDVPQVPQFPQVGSELLNMSSLMMDDGFFKMPGDLHLPSDSEALASVITKADLWSNPMQQLSSLNGSTWEDSRPSKSSFTSQLQNLSGYEDRRNPMNQVNVNMFLPNTKPQFWGSAIMNQKSLMDDRLVPHTSWDENQSTQTSLSPTESSSLEEERSLPNYFSSQYEDVPQSWAGLLASDILF

GLLASDILF

> PpDof6

MTNRAESSKALLYTLLSSLDSDAQMHVELKSLKNTLHFGTSSRLDDADTKLELKTALGEEDEIRNLCYRASPSSLHSKSADLKAILEEPGEKTLYIEIRKSPSAGGGCGAKSLHTNFLSLSDGHRSCSDVYESTRLADYMLQSDHQFLEIESGAGHSNVDDMQRPGSANEELLETAHAIVNMADLKSNTVLPQEYHEDGTPRRRQKVAVEPKELVGIPKECPRCNSKEVKFKYFNNKKLNQPRYQCLSCKDFFTHGGKKLATEAASPSASVQLMLPDGSFNILPIQSLDGKGKERKRKAKEPPQFAGMEKLCRKCGSKNTAFKYLNNGQLDQPRYKCLDCKGMFQLHNKRVRPSSSGDVNTAALLSDLSKETTSVESASVPLESALLICGPRVAKQTERKRKAMEPEHLVGVVKPCPSCKKTKTRFKYFNNKNMKQPRYECLDCHQYFTYKTEFNQVNSGAFGGDQGFVASITAVVNEGNSEGPNGSDRNGRDKEVEGNTSLTGRSDSERIYEQDLLRLNGEGDANAINSGHLKDRMKDGGSGLASSGFIGTNMRVRDTGEVINVRRGLGQVEREGGQGGGTALEVIGFRRMLDLPGSLE

KHDKVFDSTGVKQQ

>PpDof7

MTNRAESSKALLYTLLSSLDSDAQMHVELKSLKNTLHFGTSSRLDDADTKLELKTALGEEDEIRNLCYRASPSSLHSKSADLKAILEEPGEKTLYIEIRKSPSAGGGCGAKSLHTNFLSLSDGHRSCSDVYESTRLADYMLQSDHQFLEIESGAGHSNVDDMQRPGSANEELLETAHAIVNMADLKSNTVLPQEYHEDGTPRRRQKVAVEPKELVGIPKECPRCNSKEVKFKYFNNKKLNQPRYQCLSCKDFFTHGGKKLATEAASPSASVQLMLPDGSFNILPIQSLDGKGKERKRKAKEPPQFAGMEKLCRKCGSKNTAFKYLNNGQLDQPRYKCLDCKGMFQLHNKRVRPSSSGDVNTAALLSDLSKETTSVESASVPLESALLICGPRVAKQTERKRKAMEPEHLVGVVKPCPSCKKTKTRFKYFNNKNMKQPRYECLDCHQYFTYKTEFNQVNSGAFGGDQGFVASITAVVNEGNSEGPNGSDRNGRDKEVEGNTSLTGRSDSERIYEQDLLRLNGEGDANAINSGHLKDRMKDGGSGLASSGFIGTNMRVRDTGEVINVRRGLGQVEREGGQGGGTALEVIGFRRMLDLPGSLE

KHDKVFDSTGVKQQ

> PpDof8

MVNVEKPHMSVAVLGHGDWAMIPSSSSHEPQQQTQQHQQEKKASQLFAQRALQERRLKPPDQVIACPRCQSLNTKFCYYNNYSLTQPRHFCKSCRRYWTAGGTLRNVPVGGGCRKNKRTKPRTPEGSNSGTGSADVDSMSSSGLQHPGFTPTSIGFLSSSEGSPYGLADPSIRADSSMSQSSKIGLPMPYQPGGQSSALAQHQQLHPFEGGIPGLGFFRNPGRVAETALNLHPTFQMPTGDLSEMFDTSSVSRTGALAALSEGLGTFNVNAEGGRDQQGFVSLERWQQAAKQHSSSGMWGSHEIAQAQSNIGMGRQQDVKGKMTLGLVPITGMDTSKGQQQQRPTPSWQHSNPPENSGLHFEQQPEQNYWNNSGGWPSMDSQAYQSSGGGQAL

> PpDof9

MQLPAQQQNDSAALVATVPHGSSNALNGGGDAPASPSSPGSPSKNAIATREKPDKVLPCPRCESMNTKFCYYNNYSVTQPRHFCRQCQRYWTAGGTLRNVPVGGGSRKKSRHPRGIAEPYLQRSGAFGVASSGEQSAMQNVGFQQMLTQDMVGLNFAQLPGFGPQNPHHYLPFSMDHTQVAYVPMTNKPPCQEDLSNLHAMYGNAPMSAPTGGYYLAPELGGENSGNAVKPEFWVNNMVAPQVPVPGSNPNQTVNRNGCLDSSMLLNLGLNNRPSAQVSGNGKPVWDQQDVKRPLSQQKNGSPPHGSSTIEEEGSTPTNEYSNSGYEEGDTVSSMWNDMQHDMDNIFQ

> PpDof10

MHLPLQHQARLAGSFDDENEELDAQLQANSASHSHSHHSQEDDASQSQGSKGSGGDAAVPDGAPKPKNLPCPRCQSMNTKFCYYNNYSVNQPRHFCRNCQRYWTVGGTLRNVPVGGGSRKKHSRSRSRSDPYYRPEPNPSHDSDGEMEQIGNPLHPGLAAAAMAGIIPQDIASLHSEAGFPDFGELSEAPFHPNKIYSLLQLALLQNEGMYGIPGMPFTVGNPDHEQAELHQLFQGQAASNPGNLLTAAAAMMFMPELDPAAVALLHKATLWAEAQQVQAMARRQTAQSAPPWVEERECKPTVSAHMNREIAAMEELRHRQQAAAGSHRRPGFWETALMHSRPNKRQAWDGTKPSLSPPSVQHGSASTVDEESGNPVNASGFSHNYDDTSSWGSAGFHPGPDLYYQ

> PpDof11

MQLPISHLSEFQETENEEIHAHLAANSGSHSHDEEEGSHSQGSKGSIGPDGNPVKDLPCPRCQSMNTKFCYYNNYSTSQPRHYCHDCQRYWTVGGTLRNVPPGGSCRKRTTTVPQSSSVPHFHPGTPPSSTLTGSEGESERYQHPGSPPMMAALASMLAHPVTSGFGGFEEQFADLLSFYPTQFSSLLQLAIMQQNQQDMLTSTFPDASILASGLLMPEVFEPSHLELMHMATMLAEMQRMQELARRQAAAWAEYHREGQSAVSSGIHRQSPQNAAAEEAAKNRQYGGSSLHRRPGFWETALRHSRPNKRRTTNPGVPESSNLSKEQGGTRAATNQPLRQEHSS

> PpDof12

MHLPVNHQILLGDFEDDSNEELDARIASSGSGSGGGDSPGSSRGNGGCIRSRPVKVIPCPRCQSMNTKFCYYNNYSVNQPRHFCRNCQRYWTVGGTLRNVPVGGGSRKKIRTRNRNDTYLLGGTPQTSPVGSLFGSGGDLLSSSACMQQLSMLPPTGGMLGFGNQTSGFAPAVPPLPYLQFALEYTQGLHPSVQKPLAGLQEVPDIQSLYDTQASQAPQVNSSIFNSSSSLMLNSGVTLPTLHAPSHSDTLPSVMAKTGLWTVAAQQLPSLNGTTQVQSSWQDKQSKPSMSSQLQHRSGYVERMNPINHMNAIMFPHNVKPGSWEAALMHSRPVMNDRLVPHTSWDENQSSTHTNLSPNASSTTDDERCPSNNCFPHTYEDVSQSWVDLQASDALSFQ

> PpDof13

MRLLHWKGYLQIPILEPIEFMVQIKVCPRCKSSTHVRFLDLNNKSPKQPRYRCRKCKLNFSPFKTRGQVPKKVTNEAVQAMDTSTELIDAPDQVSQLLALYEISENLIQGIELLESTLDLLQWKHGLWIVLGDCPMTWVPLLFFLRTKRMVVLQCLGRSLRPAALTHTGFSLETCVSEC

> PpDof14

MSKLRKWMMKEDRESSAGSADTSAIKLFGATIAVSITDSGVSSKAATSMNATVAKPCVSLTGSEEVDGASMQLEADEMGSSLGSTCNESSAGALNTSNAEMAIKDVNGEAQVFATFTRELPQSVMQTIHGMVSQQKQRALTVEEKKEQCSKKPDKLIPCPRCDSLETKFCYYNNYNVNQPRHFCKSCQRYWTAGGTLRNVLVGAGRRKNKYGASQMKQEDAEGANSGITTVRMDHHEGAQPMLPCTINPYVIKVPPMQLPQLATQCSPRQVSDVQSTVTTSCKAVSSASAFQPAVPEMSTDSVSKLRQRSGTESTFEGKETSSSISSVSATETTSKYVLSSRVPPQQQADQQAVWTKNSVPLGYFNGAWPYGYNIGIGGVGPIPDNPAMCGPHPAAALASSNLPNGMSPPPVNVWTGMWASGMPSGMPGMAGVPAVPFGWNVCQGGWTLPWRPVPAVAVQPPAEVMHSGSARKWDSASSEDRVRKMARVEIPEKDVL

> PpDof15

MHLPLQHQARLAGSFDDENEELHAQLQANSVSHSHSHHSQEDDVSQSQGSKGSGGDTTPNGTTKPKDLPCPRCQSMNTKFCYYNNYSVNQPRHFCRNCQRYWTVGGTLRNVPVGGGSRVKHSRPRNRTDPYYRPGANPSNESDAETDQSGISLHPYLAHAMAGMVPPDMAGLLPDAGFPDYPDPAEFPFHPSQISNLLQLALLQNPSMYGMPFPVGNPERDEAETLQAMYEAQAAPNSGNLLAVAAAMMLMPDLDPSTMSLLHKATLWAEAQRAQAMSRRQTTQHAAQAASAWAEERECKPSVSAQMHREIVAREELRHRQQVAGMHRRPGFWETALWYSRPNKRPAWDEASPSPKPAQQGSTRTLDEEAGNTANASSFSHGYDDTSSWGSVGLNAGQDLFFS

> PpDof16

MDSSSADTGGDKPRVLKSATLLSRGLGSSGEGAGFRGQSGPAHVNMANGGSGPQKDYSLHGTQLNRKEGAFADHINLNERREELQPSLHARDVEGRVAGFGGGPSGVGAHISGENPIKRDEEGNHEDDGKGGAADELPGDEANVSSCSPLPFGRQEAKVVPLESIHSKAKGVEIIADGSALKSANNSKNEASGASASVQIASETTERNGPPLSQAVGDEDATVGPPPSHAPGDDADTAGPPRPPAAADEATVVPSPKQGKGQKQKPTLKKPDKLAPCPRCDSLDTKFCYYNNYNVNQPRHFCKNCQRYWTAGGILRNVPVGAGRRKIKHSSSHPRHGAVPDIPAVRLDSLDMAQLRPCLSRGSSVPHQVSPLLTCNASSPSNLNSLLNRQERGSSEAITQTRSMHVAEAVRRHYISESQVNIASDSGNRLLHAEHSDGCEPVLACEAEKDDCVEACTSSATGSPSRTNVTPPTQEAQSMLRGPPTTLIPVCGGVVRPVGILDGSVWTAAPSPFGLFNGSWPYGYNVGWSGPAPGAPGPPICPPGGTLVSCQPAVTWSSPPNGIWSGICWGSTLIPGMPTGMPGPPWMSPGWGGGWPIPWGPDAAAAAAAASASASFQTASAPMSGSGFPPVQSRLPIRPVLNKHPRDRPDLDRVESSLWLPKMVKVDDSGSAPRSSVWTAVGAENRVETVVSGSNFKAFLPVADSKASDTS

> PpDof17

MIAAFGGARVPDNLSSFGRDIRFHPWEQHGQLAFTNQAGPSKAQGYMVNVQTPLMSVAVLGHGDWAMIPSSSNHEQQQQQAQLQQQEKKASQLFAQRALQERRLKPPDQVIACPRCQSLNTKFCYYNNYSLTQPRHFCKSCRRYWTAGGTLRNVPVGGGCRKNKRTKPRTPEGQNGRTGSADGDSMSVPVLQHPGFNSTSIDFLSSSEGSAFGSADPSMRADSPMCQSTEGGLPMFHPGAQSSSIAQHQLPQPFDSGIPGLNFFRNANRVPGTTLNLHPTFQMPTGDLSEMFDTSNAPRVGALSTLSEGLGTFNINPKSGRDQQSLMSLERWQAASKQHAPSGIWGLPEMAQAQANADMSRHQDAKGKMTLAMAPASGMDAPKRSQQQQQQQQHQQRPTPSWEHSNPQENPGLHYEQQSEQNYWRNPGGWPSTEAFQSSGGGQAL

> PpDof18

MYLPMNRQKVLRDFEDDSNDELQAGLNDSRSPSGEGDSPVGTRGNGGCIRDKLVKVIPCPRCQSMNTKFCYYNNYSVNQPRHFCRNCQRYWTVGGTLRNVPVGGGSRKKTRTRSRTDPYLRSGTPQTSPIRTLSGSGGDLLSASACMQQLSMLPPSGGMLGFGDQLSAFGPAGLQLPSSHLALEQSLGTYPPLQKPLSGLQEVPDLQSLYEVPQVPQIPQVSPAMLSSFSPMMMNSGFFLPEMHVPSQSDALASVMAKACMWSNPAQQLPSLNGISQAQRSWEESQSKPPMNSQLQNLSGYEERAAPANQMNASGWPQNLKPGFWEAALMQSHPVMNDRLVPHTSWDENQCSTHTNLSPNASSTTDEEWCPPRNCFSHGYEDASQSWVGLQASDILRYQ

> PpDof19

MVHLPASKDDTSVGSLEKESRKLTLGATGTPASPASPGKKALEKPQKVLPCPRCESMNTKFCYYNNYSVNQPRHFCRQCQRYWTVGGTLRNVPVGGGSRKKNRHQRQEPYLRTGPGSVGSPLAMATAGGQPGMPNMGFQQLLTHDMSGFNFSQLPGFVPQNHYPTYSVDHTQGMYTQPLSSKTAQQEFQSMRPMYGTMIPHSMPPVTGNYYGPDLGSQLQVDNAVNTNGTVGSVKPEFWGNMVPQNPHPQAGNHPVFSMWEQSNPTSATPASAADQGAGKTGVPATLQTIPKNGGYDSSMLLHLGLNSRPQLGGKPAAWNQNQLDSRGGTLPTNGSSPPHGSSTIEEEGSTPTNGYSNSGYEEGDTVSSMWQDMHDMHDIFQ

> PpDof20

MSSEKLCDRREGLLLLSANPKFCAGVKFPGLTNRVVSDSLYQELPSEEGKGVALDVVEQATMPDPFEWTLGVQPGCPKATMDPEIKLFGQTIGVASSRADAGADAGVLKPTSASPPWNTARSSKQDGGDSSLFRNENEGGVVEKLRTARSSSRDEFLQPPKSTELVPGNRSTMIFSLGASDSEGAHSDELKDQGEGDHSQYLGDDKHLQKPDKVVSCPRCDSLETKFCYYNNYNINQPRHFCKNCQRYWTAGGTLRNVPVGAGRRKNKHGVVQRDCTEASVNCSIQSDSSDSASQLLPCALGSPTSQKAGSSLKHSQPKRLVGQGSPMRSPRSTMSFSQDSGITLPPPFSLHRNGPIPYQSFSSSQGFLTSGSEVYTVGSSDSSSVTIGQRIARFPSVNGMDKDCSRSSLSGQLQATHSLRSMTTVYNPSLQLESSSGMHRSVLPPGSSGWPGNGAPAGLHSGEWPHNHLELDGKHSTQVVGTTYNTATTLPATSSWSAANAQWGATQWGAGVSHLSNPAQMPTQVWPGGQQPMAPNATPLDHSVVAVLSTLGKRGLSDEGSSMWPSKALRPHGSRSLNSWSMSGKDQSEVNSSGNALNL

FRPKLEIGVACETKDSFVSRHFTTVPPARPVPFQVTI

> PpDof21

MSSDNLSERREGLLLPSANPKFCQGVGRWILQDQAVSDFVYQRLPLGGEGVALDVVEQASMPHPCDWTLGVQPGCPKASVDPQIKLFGQTIGVVSSRADAGADAGVLKPTSASPPRGTAPLSKQDGGDSVILRDENEGGVVENLRTARSSSRDEFLQPLKSSELVSGSRSLNTGVSLGPSDSEGAHSDELKDQGEVDHSQSVGDDKHLQKPDKVVSCPRCDSLDTKFCYYNNYNINQPRHFCKNCQRYWTAGGTLRNVPVGAGRRKNKHGGMQRDCPEASVNCSIQSDSGDSASQLLSCALGSPTSQKAGSSLKHAQPKRLAGQGSPMRSSGFGQDNGISISPPYSLHQNSRLPFQSFSLSQGFMPSAVEASTVGSSDSASISLGQRLARCPSGNGFDKDCSRSSLNGQTQDTHSLLSKQSAYNSSALQLEATSGMYRFVLPSSSSGWAGNGASVGFHSGEWPHGYHLELGGKHPISHSSQGVSYSTPDVRASLNAIQTLPPTSSWSPTLVQSGATQWGADVGHVHNPPQMAASVWPRSQSMVPSAASVGQAPVAAPTTLGKRGLLEEGSCEWPSKCLRTDDSKNLNTWAMPGKNSTEVMNGGNALSLFRPKLELAVACETKDSFMTRHLTTVAAARSGPFQVTI

> PpDof22

MVLLTASKDGTTEGSLEKKARKLALDGTTIPLTPDSPGKKALEKPQKVLPCPRCESMNTKFCYYNNYSVNQPRHFCRQCQRYWTVGGTLRNVPVGGGSRKKHRHQRQEPYMRTAPGSVTSPLALATAGGQPGMANMGFQQLLTHDMSGFNFSQLPGFVPQTLYPAYPVDQSQGMYAQPLSNKSTQHEFQSMHPACGVMNPQSLPTATGHYCGTDVGAQLQGDNGVNTNGAAGPVKHEFWSNMMPQNPHLQAGNHSVQSLWEECKVTSGTVPTTTADSDARKAGALVDLQAVAKSGGYDSSMLLHLGLNTRPQVSGKPSFSNHNQLDSRGVAHPTNGSSPPHGSSTIEEEGSTPTNGYSNNGYEEGDTVSSMWQDMHDIHDIFQ

> PpDof23

MQLPGHQNDSAALIGSKPQCSPNAALSGGSDAPASPSSPGSPTKIANATREKPAKVIPCPRCESMNTKFCYYNNYSVTQPRHFCRQCQRYWTAGGTLRNVPVGGGSRKKSRHPRGGAAEPYLQRAGSYGVASAGEHSIMQNVGFQQMLTQDMVGLNFAQLPGFVPPNTHHYVPCSMDHSDGVYAPMTNKPPGQEDLSNLNVLYGTAPMGGYYPAPEMGGENSGNAVKPEFWATSMAAPRVVVPGSNPSRDGRLDSSMLLNLGLNNRSPTQGGGNGKPMWDHQDLKPALPQQKNGSPPHGSSTIEEEGSTPTNGYSNNRYEEGDTVSSMWHDMQHDLDNIFQ

> VvDof1

MRRQQQQQQQQQLEPLKCPRCDSTNTKFCYFNNYNKSQPRHFCKSCKRHWTKGGTLRNVPVGGGRKNKRPKTSATATAAAATPTQQRKSSLALGDHEKSSLSDILYQAFIRPPLSVMQQNSIGSSNGMSMASIPSLPPNQNLHFPFRSLSSFDTSPSSIPSSFQPSNVYNYTGEAEAKDGSTTACFMPSTSGTITQPWQMPAASSVVDTTNYWSWDDIDTFVCYYYLIIAIISNRINYKT

> VvDof2

MIHQYPTNFPSSATIKKDVYSGSLCSASSVSDHLLSLSLLSLSPPPKLSNPEKHPQLILYPPLSFPSLPIFLFWVFSSSPCIKSLMMSPDDIPANPVSKDETQGSGGRKTGAHRPPDQVLKCPRCDSPNTKFCYYNNYSLTQPRHFCKTCRRYWTKGGALRNVPIGGGCRKNKKIKSSSRLSAVSTPCFSIDQPGSSSSLMGFNYPLPSVLKQGESGGFGGAIQDMGSMNVHSSLASSIESLSSINQDLHWKLQQQRLAMLFGGENQKDSSVSSVPPENQNQKLQPILFQNLEISKPEVCAVGNSRKDSASGNPATEWFFDTYTPVTPTTTNTSTGGNGNDNTNHWNGIQAWSDLHQYSTLP

> VvDof3

MGKPPITSLQKLLATKIQIHPRIFFLIYFLPHRLREKRWGVFGLLSIHVSTSLYHSHIDLTYTIFKGSKRKKRMIQELLGGGAGLIGGERKITINGGILEASPSPSPSPSPSSSSSGNTTTVTTAAAATTSSASENLRCPRCDSSNTKFCYYNNYNLTQPRHFCKTCRRYWTKGGALRNVPIGGGCRKNKNTSVSTAVGKSSAGKAKSAVSEIGKSGLGGGFDHEIPSNPILWASPQNSHLLALLRATQNPNPNPSPISNSVTVKDDGVMIGSHMTSESGVGTGAVNIARNMGLDHLNQLPSIGLCSSYWRNNQHQTQQHQQNSHHQQQNGFILGEVQNTGIQELYQRLRSSTNYYTDHSPVVLSNAVSSSSSILESAPIAGVLSNAVSSSSSILESAPIAGGELGYWNPTFSWSDLPTTNGAYP

> VvDof4

MGLTFLPVCMDSSEWLQGTAHEENGMDSSSPSGDMLTCSRPLIERRLRPQHDQALKCPRCDSTHTKFCYYNNYSLSQPRYFCKTCRRYWTKGGTLRNIPVGGGCRKNKKVASKKSNDHSINQNPGPSSHNPTDLHLSFPEVQFSHLSNLLGSHGGLGNHSFMESKYNGMLENARPIDFMEGKFEAIVGSSRNHDFMGNCELGMGGLGDMSHGGLAPNFHGLCSPFGVTLDGNGGNFMDTCQRLMLPYEGNVEQNAMDVKPNTKLLSLDWQDQGCSDAGKDSFVYLNSLGSWNGMMSNGYGPSTTNPLHFCQISMLRNI

> VvDof5

MQEPKDPAFKLFGKTIPMLADGDAPVSSGDVGDSGAAVAREDGLEEETEKDALGGKPAETKGEDGSEESRNSETVAESNENPKTPSIAEENVTLKTSKAEKGQSDSPDSQEKTLKKPDKILPCPRCNSMDTKFCYYNNYNINQPRHFCKSCQRYWTAGGTMRNVPVGAGRRKNKNSASHYRHITISEALQTENGDDHSSASSITVSNSSEEGSKYGLHEAKRNSHGVPSRIPCIPGIPWPYPWNSAVPPPFCPPGFPMPFYPAAYWNCHVTAPWNIPWEGDILKPSIVEDEETPKQRSSERCLWVPKTLRIDDPAEAAKSSIWTTLGIKNDKNEEGDWTMNRAYALSPHLAPIIIPCSHRTGIFCHL

> VvDof6

MQDIHSMGAGGGRIFGGDRRLRPHQNQALKCPRCESLNTKFCYYNNYNLSQPRHFCKSCRRYWTKGGVLRNVPVGGGCRKTKRSKAKSSSDAPRERKSNSHSSSESSSLTATTTAAATTATTEAVSAPSSNAASTLMTGSNDPAALGFNISDISPFKYQEQEITAGFLDQTVQVELSALQNRSNHGGFPSLDWQTSGDQGIFDLPGNVDQGYWTQSQCVLFSPLLGFSFFLSNVFPIKLIKYRKNIECFVCLDAS

> VvDof7

MEGGVWKANVEISPNCPRCGSSNTKFCYYNNYSLTQPRYFCKGCRRYWTKGGSLRNVPVGGGCRKNRRGKSFRILTDRLASKGLASDPDPDPSGSALADSATTSSGLHDASASNIDLALVYANFLNQMPEPKSTGFEMPELPCEFHPSFQLSSTLSTSNTSLDSSHIQLPPDTHLSEDNQVYLSGFHPIHTHHDHDSANIYGLPPLPGQEVASPEMLWPISQAMVQNQTLHETQLPALHPEAAQHPNHVAVTAGNWSPFDLSCYETFSRP

> VvDof8

MAEAKDPAIKLFGKTIPLPEVATAAAGNDSPSGATVGGGGEDWVDQNRATNSSPEEDCVRAGEEGREVDKDTSGGKVTDTRQEDGARSSTSEEFTDPDANSGVNENPKTPSADKETATLKCSKNEEEQSETSISQEKTLKKPDKILPCPRCNSMDTKFCYYNNYNVNQPRHFCKNCQRYWTAGGTMRNVPVGAGRRKNKNSTSHYRHITVSEALQSARTDVPNGIHHPALKTNGTVLTFGSDTPLCESMASVLNLAEKTMRNCTPNGFHKPEKLRIPVPYGDQVMRNLPPPAFCPSGFPMPFYPAAAYWGCTVPGAWNIPWVPQPSALNHTAPSSGPNSPTLGKHSRDENMLKASNFGEEELQKENNSERSDEKNHIAETSPVLQANPAALSRSLNFHESS

> VvDof9

MVFSSVPVYLDPPNWQQQANHQPGGSNDNPHLPPQQAPPPLPPVGGGGSGGTGTIRPGSMADRARLAKLPQPETALKCPRCESTNTKFCYFNNYSLSQPRHFCKACRRYWTRGGALRNVPVGGGCRRNKRSKGNRSKSPVTGERQGGSSSASAVPSNSCTSDMIGHLPPPPPPPQLSFLPPLHHLADYGAGDIGLNFGGIQPPVAVSGGGNSGTDIEFPIGSCSGGGGPILSGGLAEHWRLQQVQQFPFLAGLEPPSGLYSFEGEGTEPPSYLGGSGHLRAKALDSGVTQMTAVKIEENQGLNLSRNFLGLPGNDQYWSGNTWNDLSGSVKAKSAQR

>VvDof10

MQDPSTFQPMKTHFPEQEHLKCPRCESTNTKFCYYNNYNLSQPRHFCKNCRRYWTKGGALRNIPVGGGSRKNTKRSSNPKRPSSSSSTTEECSTSAEVSALSWPRTANSEACWRILEIQSRYSSSSMFPHCLKRARQGSTSPSFWILFFIIAMLFFHLFGCQDSHTCLEFIFDLFLCSQMVHPCNLWMDLCMGYTQSIVKIIELVFHVHVGTFACCFFRVVEGIAIFHGVDHIPMVFGC

> VvDof11

MDTAQWPQEIVVKPLEEIVTNTCPKPALEKRARPQKEQALNCPRCNSTNTKFCYYNNYSLSQPRYFCKACRRYWTEGGSLRNIPVGGGSRKNKRSSSSSSSSSSSASSKKLPDLPLKISEVFGAAHWDHFKGVEFLHAHANS

> VvDof12

MLDFKDPAIKLFGKTISLPLNPHLSPTSPPPPPLSSTTSFPDDTSQGLQPPSQDQKPLEGQEFEGKEEDGTSRQTSEELKDPTASPGVSENPETPSADKETSKDGEQSEISGSQEKTLKKPDKILPCPRCNSMDTKFCYYNNYNVNQPRHFCKNCQRYWTAGGTMRNVPVGAGRRKNKNSSASQYRHIMVSEALQTARASAANGIHHPALGNNGTVLNFGSDEPVVKNFQAFSPHVPCFPGASWSYPWNPAQWSSKIPPPAFCPPGFPISFYPAPAYWGSNPGKEDHQKENNPERGVWIPKTLRIDDPNEAAKSSIWTTLGIKNDGSNGGSLLKAFQSKGDEKKRIAEMSPVLQANPAALSRSLNFHERA

> VvDof13

MQQDRDKGGGLEDVDPPDRRLKPAQSENQQQQLPQKCPRCDSINTKFCYYNNYSLSQPRYFCKTCRRYWTQGGTLRNVPVGGGCRKTKRKPSASGDNSRSQLPTQQQQQNLAAPPPNIISTNSGVAVGPALRIAEPANLSPAPAPASAPTLSSVNPYYPAGAFLSSLPAMRPLNQPQPQPFNQPVNVGRDFGGSNLAILQGFNVPSLGSQQQQRQQAEFYQMGNRDRNIESLYGSGEDLIQSSRPTGHQNNWHQTFINPTAPDSNLWSIGGSSNNNSNAASSSFNQNHWADLQGYGPPP

> VvDof14

MSFFLVIIFQEINSETLLRFICNAALSLSSFTSLSCSWILNEGSSRHLLFVCLLFVSTIPLRKEMLGNCEKMVVISSTTNDWPENQIDDKGLMASTGRVMEKPGQEQQALKCPRCDSSNTKFCYYNNYSLSQPRHFCKACKRYWTRGGTLRNVPVGGGCRKNKRVKRPASSVDAPSSAPTAALNPPPPSQIDLSASSNHVTPLFYGLPSNPSELNLPFPRFNSRASNEDTVSGYDLQPQLGALGLGFSSGIIANDTRTDDYRNGFNPAKQIQDLVTSSSLLSNYSIFGSSSSTTTPTMASLINSSLQQQKFMNGDLKESRGANHFQPLWNSVPCQNQIEQIGSSDPSLYWNLTSLGAWSDLPNMGSSVTSLI

> VvDof15

MYNFVLCLCVFVVLGAFVVHAAQDAPGIKLFGATIALHGGEVKEEANNKGDHKEEKRPEKIIPCPRCKSMETKFCYFNNYNVNQPRHFCKGCQRYWTAGGALRNVPVGAGRRKSKPPCRGVAGFLESCLFDASAGVHQFDLEGVVVEEWHMGGFRHAFPAKRRRSSSSGGQTC

>VvDof16

MVFSSVPLYPDPLNWQQQPNRQEGSGNENPQLPPPPPQAGSLGSIRPGSMADRARVAKIPQPETALKCPRCESTNTKFCYFNNYSLTQPRHFCKTCRRYWTRGGALRNVPVGGGCRRNKKSKSSSSKSPASTERHAGSNSTIGLSAEGVEQWRLQQVQQFPFLGSFEPPTGLYPFQNEDLTAVKLLENQGLNLSRQFLDTSENNQYWVGNAWTDFSGLNSSSTSNRL

>VvDof17

MDASSAQRQEMDTQSLESMLVCSKAQQERKPRPPEQALKCPRCDSTNTKFCYYNNYSLSQPRYFCKSCRRYWTKGGTLRNVPVGGGCRKNKRSSSSSSSKRTQDQPLTTNPNPLNVLPPLTYDSNDLTLAFARLQKQTGGQLGYDDHHHDLALMGNPNNTHCDVLGNQCVNTCGTTTPGLLDALRTNFLETPSGYHNLYYGFEMSGATTTAVTVTTMKQELCHGREGENRVLWGFPWQQLNPDGSMGEVDSGRESWNGLASSWHGLLNSPLM

>VvDof18

MKEKENSIGEAKDMEEAISISINSDHTCSKPVLERKARPQKDQAVNCPRCSSTNTKFCYYNNYSLTQPRYFCKTCRRYWTEGGTLRNVPVGGGSRKNKRSITTSSSLSSSLTSSSSFKTHLPDLNPPILSHFSSENPRIHEPHDLNLAFPLPQYYHGISQFAELLKTGITSRGLSSFVPAPMPDSSSTLFTSGFPLQEFKVPHLSFSAHLHGVDPQQNMSTTGRVLFPFGEVKQLPATTSTGVDHDCSGNNKGQGSPATAAYWNNGITSVMNLIEKWSLSQLVTTNLHFMEVLFLSI

>VvDof19

MNGGDLAVSRDPAIKLFGTTIQTQICSNSPQKSLDAPSEITKAEAEDPCAEDSRKPDGSSVSEDGKEEQQTQVQMSGLQVHKDQGETNSSAQEKVLKKPDKILPCPRCNSLETKFCYFNNYNVNQPRHFCKNCQRYWTAGGTMRNVPVGAGRRKNKHLASQYRQIMVSSDGVPTTVIEASDSSNQQILSCGETSTTFRPSTASGTVLKFGPEAPLCKSMETVLSIREQKRCAEMRTVNCGGNGEEPSSCASSVSAPSFPENEFPENVGHKDRPSLPASPPVAPPQCSSDTVYAPNNSNPNSVQWCSRPMLAVPGFCAPTIPLQLVPPSYWGCMPIWGAGTGNISLAGSNDCLSPSSSTSNSCSGNASPTLGKHSRDAQPAEEQKLEKCVLVPKTLRIIDPDEASKSSIWATLGIKPDQKAPISKGGIFKAFEPKSGAKTDLSDATQVLEANPAALSRSQTFKEST

>VvDof20

MQSEPGNHQRPTGPPPPPENHHHLQCPRCNSTNTKFCYYNNYNLSQPRHFCKSCRRYWTHGGALRDIPARDNAPKRSRSRSSPVTPPLASYPPQRVPPDLNVGASGSFTRLLNSHGSEFSGLGAEYGLGSGLHEVGDGGAWQIGIGEEVELVEGNGFTWPDIHVSAPGRILKE

>VvDof21

MVFSSMPSYLDPANWQQPHNHEAGSSGLPNQPPPPPPPPPQPHGGGGTGSIRPGSMADRARLANIPLPEAALKCPRCESTNTKFCYFNNYSLSQPRHFCKTCRRYWTRGGALRNVPVGGGCRRNKRSKGSSSKSPVSGDRQAGSSSTGAIHSNCSTAEILGLAPQIPPMRFMAPLHHLADYGAGDIGLNYSGIPTAVGGTGDMNFQIGSLEQWRLQQTQQFPFLGGLDPPPGLYPLEGGVEPSNYVGGSSQVRPKLTGSGLSQVASVKMEDNHEMNVARQFLGIPGNDQYWGGNAWTDLASFSSSSTTETSGVGRNRKLQLTM

>VvDof22

MDTAQWPQGIGVVKPMESSGPVAERRARPQKDQALNCPRCNSTNTKFCYYNNYSLSQPRYFCKTCRRYWTEGGSLRNVPVGGGSRKNKRSTSSSSSSSSPASSKKLLPDHLITRFPSSASQNPKIHEGQDLNLAFPPPPEDYNNSISEFADLGLGSFMPMSVSDSNSIYSSGFPLQEFKPTLNFSLDGFQSGYGSLQGVQESGARLLFPLEDLKQVSNTTEFEQSRGVQGDSAGYWNGMLGGGSW

>CaDof1

MDTANWPQEIVVKPMEEIIGSSKPNNCVERKLVRPQKDQVVNCPRCNSTNTKFCYYNNYSLSQPRYFCKTCRRYWTEGGSLRNIPVGGGSRKNKKSSSSNNNNNNNNSNINHVVINNPLMKKLPDLIVPPLIQHDIEEYPERHFGNRPDNSTVLPQHHHDQNPRKIIHEGSQDLNLGFSSDFKTITELIQVANYDGGNKDNNNSTNNISTLPPPSEPSSPAFSQLPSLNFSLDHHGLGNNNVRSGYGFFFPFVGLKQVSNASDHVRDQSTNNGYWNGMLGGGGGGSW

>CaDof2

MTCDSEIKLFGKILPVVVSVECDVAGGTSSGGDGDRCLEDGKASSADEGSENENQGADKDDLTGELNEAKFEEGDQSQMMEESENPRTLSESENSSKSPTDEDSQAVKTSGTENEPTNVTNSEQNSLKKPDKILPCPRCNSSDTKFCYYNNNNVNQPRHFCRSCQRYWTAGGTMRNLPVGAGRRKNKNLAAHYRHISISEGLLAAGVESPNGLIHHPMFKPNGTILSFGTDLPLCESMASPLSQAEKRLSNGIQNGFHKAELKNSSCKVGDTGDECYKGSNIPTPNVMVEEGKRELHKAVMHNINGIPSPFPCLHGVPWPFTWNAAVPMPAICPIPFPMPWLGPALQAASEKTSGSDPTSPLGKHSREGDLLKPSNPRGKEQSEQKYSERSILVPKTLRIDDPDEAAKSSIWSTLGIKYDSANRGEFFKALQPKSDDKHNKANTPPVLHANPAALSRSITFQQSA

>CaDof3

MERGTIWKPNVELAPACPRCGSTNTKFCYYNNYSLTQPRYFCKGCRRYWTKGGSLRNVPIGGGCRKSRRGKSSSNTIHIHHHHELISRNLGHGVCLNPTNIDHHNQSTSSSLDHHHGPSIDLALVYSNFLNSTNSKSSQPEDRQNPELPDDLLLPDQGVLVTPSFELSSMIDMEFVNSELGQESRLLGAGAGDGVDFYFSGIHEEKQNGMNHSDVHDDHYTNNMNVNANNSINHDHQLGNNNYMDLPPLPCEEIMWSNSHDHHHHMVFPNDLLRTSHNLTTGGSEPEPEPESAVQNPSHDHSANNANDGSLFNLSNFGNIFRP

>CaDof4

MERGTIWKSNVELAPACPRCGSTNTKFCYYNNYSLTQPRYFCKGCRRYWTKGGSLRNVPIGGGCRKSRRGKSSSNIIHNHHHHELISRNLGHGVCLNPTNIDHHNQSTSSSLDHHHGPSIDLALVYSNFLNSTNSKSSQPEDRQNPELPDDLLLPDQGVLVTPSFELSSMIDMEFVNSELGQESRLLGAGAGDGVDFYFSGIHEEKQNVMNHSDVHHDHYTNINVNANNSINRDHQLGNNNYMELPPLPSEDIMWSNSHDHQYHHMVFPNDLLSTSHNLTTGVSSEAEPEPEPESAIQNPSHDHSANNANDGSLFNLSNFGNIFKP

>CaDof5

MSELKDPAIKLFGRTIQLPDVPDSSETMLEDSLPEEANGEEEDVEDQKDNIGGNLDDEEDEMEILTGKELQDQNSHPTKTDSIKVQPVGSDCSTRPSKSEEEQGEASNSQDKILKKPDKILPCPRCNSMETKFCYFNNYNVNQPRHFCKNCQRYWTAGGTMRNVPVGAGRRKNKNSIPHYRQISVSETLPSAQADYPNGIQQPVLAFGSPTPLCESMASVLNIADKTMHNCSQNGFHKPQDPGVPVSYGAGDNGDDHSRRSSVTTANSEDEVSKIVPDQLKNCHNFPPYVACYPGAPWPYPCNSVPWSSAVPPPGYCPPGFPMPFYPAASYWGYTVAGSWNVPWISPTTGSLIQTPPTSGPNSPTLGKHSRDENILKQLSNKEESSKENNPEKCLWVPKTLRIDDPREAAKSSIWATLGIKHDSVESVGGSPFNAFQPKNDDNISVSENSTVLQANPAALSRSVNFNESL

>CaDof6

MAEVQESRISQGIKLFGATIQVQEIKQAKVHQPTNKVDQDHDNNNNDQEKRPDKIIPCPRCKSMETKFCYFNNYNVNQPRHFCKGCQRYWTAGGALRNVPVGAGRRKAKPPCGPGPHGDMNGLSDGCFFDVTNHHGNNNNIHQLEFDGVVAEEDQWHLFQAAKRRRSTSHTQSC

>CaDof7

MALIPSSTTNEIWPQIDEKNNLMMASNGSSSSNTRDMEKPIPDPSQQPPPPPHLKCPRCDSSNTKFCYYNNYSLSQPRHFCKACKRYWTRGGTLRNVPVGGGCRKNKRIKRPSTNSSSSSSCTAHDIITTSTPNISTLNPSHHVAHNSIDISSTNSINPLFYGLTSERSDLNIPFARLFNSRVSSHATVGEGQVYSLTDSIPGLMDRRMGLGFSNSSVGGVNIMGENNNYGHGGFNPIKQIQDVVMTSNCTTSSTSLLSTYPNMFGSSTSTSTMASLIASSLQQQKFMSNINGNNFHNLAPNYEELQMSRGDNNNNNSNVHEGGGNGITMLKAEKMDLSNHQIHEQIINSSDPSLSWNGAWLDPSNMGSNSVPSLI

>CaDof8

MEQGGRSSGESDRNQQQRRMKMPENNSSQPQPPPQKCPRCDSNNTKFCYYNNYSLTQPRYFCKTCRRYWTQGGTLRNVPVGGGCRKGKRTMKGGSVGVSCGSSSSASESSSRSYQQQSQQIPNLSAAAAAVFFSGNNNSRSQPPPLPSLSSLYTGGVGGGGGFLSTLASMQSMTQLSQGVNNDHSQLGVISASNSSQFGNFNIPSSIPPKVQINQQMESGIYQMVVNREKPMESSFYPSDQISQFQPTRPLGSWTQRFINNNNNNNIWPNASASSSSSGGANSSTTAAGASLNPNQWPDLPGFGPSP

>CaDof9

MGLSTKLVSIDDDGLDDWTCSSQNSLPEPPLIRRQPPSKPEPLKCPRCDSINTKFCYYNNYNKSQPRHYCKGCKRHWTEGGTLRNVPVGGGRKNKRMRMTTDLVDHITGRKRVTLEEMNDQRCPLISTTITNTTTSSMPSTIISNMDEDIKNIPSLASSSLPYDIFSSLKLSSIPQDGNTHFSLIPNSSTTQLSSNVYCNYDYMGKFDSTMEESTITTVMPITSSSDLFSQPWKVPETSNDFIIENMSSNYWNWNEFDTLSTAADLNIQWDDLEIKP

>CaDof10

MIQELFAGNTTLIGGDNNISKLSNITPSSSPLSCTTSNSSIAPAAAAGATATANASSPSNVESLRCPRCDSPNTKFCYYNNYNLTQPRHFCKTCRRYWTKGGALRNVPIGGGCRKNKTITTAKSSAAKLKNSIPFEFIGKSGIFGGFEQEIIPSNNNPFLFSTPHQNHNPILSLLRGNHHNLNLVKDEQKSIEVNLHNQFPSNSLSSLWKNNDTIVGEVQNSTGFQELYQRLKASTSRCYPDIHGPSSSSSSSMILESAPVAGGELGFWSPSFSTWTDLPTANSAYL

>CaDof11

MPSDVNERRVTKQQQGGAPAPEPEHLPCPRCDSINTKFCYYNNYNFSQPRHFCKACRRYWTHGGTLRDIPIGGGSRKNAKRSRTITTNSSLSSTLSPRDYQHASNPSAFLVPLTADHGGSLPFDVKPNVNMCGSFTSLLSSAQGPGGLLALGGFGLGVGVGSGIEDMGFGLGRPIWPFPGVSHSNVENNSANGTGASMLGSTWQLASGGEGGFVGATAGEIFNFPDLAISTHGNRMK

>CaDof12

MSEAIASRDPAIKLFGRTIHLPLFPAPAPENTGYYCSSAGENEQKHEDQNPIQQKCDITKELPDYYDCSTAKTSKSEEEQDETSNSQERNLKKTDKILPCPRCNSMETKFCYFNNYNTSQPRHFCKNCQRYWTAGGTMRNVPVGAGRRKHKNSVLHDSYSSVSEALSKARTNFPNETQQPPLTISGTILTFDTDKPLSESMVSALNVSDKTMQNYSGNGFRKYKELGIQAGDKGDDLSDGSSVTVVSSKDSDNGLPDTLRQNCNSFSNHLPCFSGAPWPYIWSSVPCRNVVPPPGIPVSLFPATTYSGCTILGSWNVPKMPPPTASQNQVPLTSGPNSPTFGKHSRDENVLNSMGTEEEPRKESNPGRRIWFPKTSRIGDLGEAAKSSIWGTLGIKHEVVDSVGGGLLKAFLPRSDERNCVSETSTLLQVNPAAISRSLNFNESS

>CaDof13

MQDPSIYSQIKPQFPEQEHLKCPRCDSPNTKFCYYNNYNLSQPRHYCKSCRRYWTKGGTLRNIPVGGGSRKNTKRSSSASTSKKITSTTTTTPLTSSVSASSSANPKPEPFGIPAIPSFDVTTGPFSSLLASNEPQFGNLLEALNPNNSNNNGSNIQLSEFSRNPISSSGLGLGSGSGSGQNHSNGGESNNCWNGGSNGWPDLAIYTPGSNFQ

>CaDof14

MREVKDGEIKLFGKKIALPENGKMLPVIVSGEDSDVGKSVSGSEVVTGEESSTGSDRGDPCLVDKEGNTSSESDGGSEYEKEDADKDQMTRELSEANLEEKYQSQIMEESENPKSPSENKSKTTTDDDSPTAKSSRTEGDQNDAAANSQQKPLKKPDKILPCPRCNSMDTKFCYYNNYNINQPRHFCKSCQRYWTAGGTMRNVPVGAGRRKNKNSASHCRHIMISEALEAARIDPPNGFHHPAFKPNGTVLSFGPDSPLCDSMASVLNLAENKTPNGIRNGFYRPEHKNPSGLGGENGDDCSSGSSVTTSNSMAEGVKNRAPEAVMQTINAFPSPVPCIPGVPWPFPFAAVPFPAVSPSGYPMPFCPPPPYWNCSVPGPWSLPWLTAPSPTANQNGSGSAPNSPLGKHSRDGELLKPNNPEGQKNSEGFVIVPKTLRIDDPDEAAKSSIWSTLGIKYDSVSRGGLFKALQPKSSEKDHPATTFPALQANPAAFSRSLSFQERV

>CaDof15

MDSSSSPSGGDHHINLTCSRPIIVDQRRLRPPHDHSIKCPRCDSTHTKFCYYNNYSLTQPRYFCKTCRRYWTKGGTLRNIPVGGGCRKNKKVSSKKSNTNETLATTSTNNNNQNLPEPEMPFPLHNHHFMSGTSSFVHHGNFMLDQNQAPIIDFMESKYEALVGSSSRNQHLFLGNGDNNIGMMSNAGFGHDNIIAPNFPFGMASMNMDNVNNFGMLLPYENNHHHHEELQSMNNAVDVKPNPKILSLEWHDQAGNKESSFGYNYSGSSTGGLGSWTGLMNGCYGSSTTNPLV

>CaDof16

MDTSQWPQGIGVVKGVEPSSKAVVLPDQRKPRPQKEQAINCPRCNSTNTKFCYYNNYSLSQPRYFCKTCRRYWTEGGSLRNVPVGGGSRKNKRSNNNSNNNNNSSSSNSSSTSSSLSSSKKLLSDLANPNDLNLTYNPISATAAVATTSTAGNFSNFSEFMALPLIHPANSTSSFMPNNLYTSSTGLPNLHDLKSSSLNFSLDGFENGYGSLQGGDQEAKLFFPMDDLKINVSTANDQFEENREQAAADQSNGFWNGMLGGGGSSW

>CaDof17

MSSEIGDRRPARLPAPVNGTRPSEPENLPCPRCDSTNTKFCYYNNYNLSQPRHFCKSCRRYWTRGGTLRNVPVGGGTRKNSSHKRPRTTTGAAVQEHTNPGLGSGSGSVSLMGCEVNLNESVQEGGGNGTASFTSLLTAGPVGGGFGPLGGFGLGLSGFGLGNLDWPMEQVVVGGVGGNGGDGGENDKWQLSGGEVEGGGGGGDDDCFGWPDLAISAPGTSLK

>CaDof18

MERTRKSNIEQAPNCPRCASTNTKFCYYNNYSLSQPRYFCKACRRYWTKGGSLRNVPVGGGCRKSRRSRSLRKDDNTLQSPSPAFETPGANIDLADVFAKYLNQGTANDHDDDDQDNNIILQESQDYSSIGASLSESPSSDSLVNNPTSFENESLFDETIMASFQDYPCGNFLQEEQGGPIDQVGNQDFLDFNTSFLEMQAMLGDEIIGQGEEFDHYNTSNFSWQSMMQFQDFGSILELDDQLKNSTSNLASDNNNYSSFDLSN

>CaDof19

MSEIRDPAIKLFGKTIGMTQQETNCVHDHHTSASFDNDKIALGGELTQSKQDDVLVDPTADSSVEPETSSGISDDLKMQDAEKEILSSKSIEEEDSSEEKTLKKPDKIIPCPRCNSMETKFCYYNNYNVNQPRYFCKNCQRYWTAGGTMRNVPVGSGRRKNKNSSTSIYPLQAGRVEAAHGMHLPALRTNGTILTFGSDKPLCDSMASALNIAENSHNMNRNEYCGSERRMPAIANDQSNGTCSTASSITDKESNSGPHDLANWSNFQPFSPQVPYFHGAPWPYSGFPVSFYPATPYWGCTVASPWNVPWLSSDQSVHNTSPASPTLGKHSRDESKFDPSQSRRRDATLQDREGERCVLIPKTLRIHDPNEAAKSSIWSTLGIKNEKIHSTHGTMLFSSFNPKADLRNHERDASLLLQANPAALSRSLKFRESTQ

>CaDof20

MAFSSIPLYLDPSNWQHEQENQQQQQLGVTNHEMNYPSELSPAVLPPPAATGSGGPAGSVRPGSMTERARLAKIPQPENALKCPRCASTNTKFCYYNNYNLSQPRHFCKTCRRYWTRGGALRNVPVGGGCRRNNKRSSKRSRSTKSPNRSDQRSRNNVPTISTSTITFPSHLPLPNTSTHLSFLNTPFHNLNDFNSTQNDMNFGEIQSHEGDGRFIDQFRLQQMQQFSFFPPLEQQPSNLYPISEFGISHDLENVKVEENKSSINSQGMNLQRNNTLGVNQFWIDYNISSTSTSQLL

> CaDof21

MSEVRDPGIKLFGKTIILPIDDLRSSSINTTSHDDQITSEGELTQSKRDDFTNSTADESVEPEISSGISDDPKAQDAYKITLSPKSTEKDDPNEASGTQDKVLKKPDKILPCPRCNSMETKFCYYNNYNVNQPRYFCKKCQRYWTAGGTMRNVPVGSGRRKNKSSSTSSYRHIMVSDALQAARFEAANGMNLPSYRTNGTVLAFGSDKPLCDSMASILNIAEKSHNSIQNGFNGSEQRMIASCGGKEIGNDRSSEACSTTSNSTEKGNDSTARDLAWKNFQAFPPQVHHFPGPPWPYTCNAAPWTSAVPPPTLAPSGFPVSFYPPPPYWSCTMASPWNVPWVSPPPSSASCSVHGNNPNSPTLGKHSRDESSFNPSNMAKEDTLQDKDGERCVLIPKTLRIDDLDEAAKSSMWSTLGIKNDKNDSANGTRLFKAFNTKVDERNNESDTNLVLQANPAALSRSHNFQEST

> CaDof22

MDPSSAQHHHQELSSQTLESMLVSTKPQQDQKKPKPPEQAIKCPRCDSSNTKFCYYNNYSLSQPRYFCKSCRRYWTKGGTLRNVPVGGGCRKNKRSSSSSRSSSQEQHSINIPNCPTNPFSYDSSDLSLAFARLQRQESGPLGFENHSNISMIMCNENPSGFLDALKGSTGFLENNNPNGFHHQNLFYGVGNIINGDMGLHNVENGGMGVINNNVSDQEMGLMHNYDQEISSGTVTTTTATTMTTVKQEMCNMARDQGDNKVLWGFPWQINGEGHNMSDFDSTRRMWNGVGGSSWHGLLNSPLM

> CaDof23

MVFSSFPVYLDHPNLHQLQQPDGHQQVGNPGLENPQLTALQPPPVQMGASPGSIRPGSMVDRARLAKIPLPEAGLKCPRCDSTNTKFCYFNNYNLSQPRHFCKTCRRYWTRGGALRSVPVGGGCRRNKRSKSSTNNNSSKTTGSNVNSTTTADPRQIGTSTSASPSSCNTEIITGRHHFPHEQSPVQFTPLMAAFQNLNHHYGGFQPPPLVSTQGAATLGHPEMGFQIGSTTNSTNNLSAPSGVSDHQWRLPSLAANTNLYPFHQGEGIESSSGNSIAHDDQGLNSTKQFLGTMENNTNQYWGGNAWTGFAGLNSSSSASHLL

> CaDof24

MGITSLQVCMDSSNWLQDTINEETEFDSSSSPSGGDIFTCARPLIERKLRPQHDQPLKCPRCDSTHTKFCYYNNYSLSQPRYFCKSCRRYWTKGGTLRNIPVGGGCRKNKKVSSKKLSNDNNITPHNVVVSSNSSPISNYPDMALSHFGNFMGINTSNNNNNINHNFMLENHHHHVPIDFMESKYEALVGTITSSRNQDFLGNVDVTAGMINGYGEMDNSGIVGPNFHGGFCSTFGLPMDGNLNYEGQNITMDVKPNPKILSLEWLDQGCSNAGNIKESFGYLNGGLGSWTGLMNNGYGSSATHPLV

> CaDof25

METTQWSTQVEIGAVKSSMGAEIGSRPGGEIINKKARPVKDGAINCPRCNSTNTKFCYYNNYSLTQPRYFCKTCRRYWTEGGTLRNVPVGGGSRKNKRSSSSQKVLDLNPNLNSHVPDHQLHQNPNKINIVGSTSQDLSLGFRTVPHDHQTSFHGVLPQFLELPKMDASNNHLGSTQISALELLRTGIASRGFTSFISSPSTPDLNALYSTSGFPFQELKLSGGNTHDHTASLSNYPSGGPGVQENGGARIMFPLGGLKQLSSTNEADHHHHQTKGQENNNSNAGLYWNGMLGAGGSW

> CaDof26

MNFSSIPYLDPANWQQQGGGSIQNHHHHQLTSPPSQTAPPPQVPPPPPVPLQPHGGGGGAGTIRPGSMADRARMANIPMPEAALKCPRCDSTNTKFCYFNNYSLSQPRHFCKACKRYWTRGGALRNVPVGGGCRRNKRSSNSKSGNNNNNNNNSKSPASSTSTDGRQATNNSGSTSTISSHSNSFTGPTSAASLLGLMTPQIPPLRFMSPLGQFSSDHHHHHHHFTPSNHMNLNFSTSTCGNILGGTTEGMMVNTNNNLLGVGVGVGVGVGAGAGAGVGGVASLLSSGNLEHWRMPQQFPNFLGGFDPSNSPSSGVSNYPFQGGVHEAVQFLGGESTSQISRPKISTSMLNQMASVKMEDSNNNNNNQDQSALSRQLLGIQGNNENWNTASAWSDLSASFSSSSTSNAL

> CaDof27

MVFSFISAYLDPSNWQQQVGYGIPNTQLPSAPPQPTPPHPLATTPPPPRPHGVVGEDSIRPGSMADRARLANIPMSEAVQKCPRCESTNTKFCYFNNYSLSQPRHFCKTCRRYWTRGGAIRSVPVGGGCRRNKRSSTNSTTTSATKSSNNNNTSKSPASSQATNSGSTSNNSCTFSSQSSAASLLGLMNPQIHPLRFMSPLGQLTDQHFTQNDNVTMNYSSFSSSSPAPVIVESTIESTNFQLGMSNNLEQWRLHQQLASQFPYNLYGGLDSSSASGSGLYHFHPTHYSSNEVGGGGGVISQIRSKVSNPMLTQLALMKMEDNQDHLATMPRQFLGHENWPSNGSHANWNELSVSFSSSSTSNVL

> CaDof28

MSSQTLESMLVCTKPEQEKKPRPAEQQPQKCPRCDSANTKFCYYNNYSLSQPRYFCKSCRRYWTKGGTLRNVPVGGGCRKNKRPSSSSKRSQDNSPNSNNPISPLSIPTISSYDHSSTDLSLAFARLQKQASGHLGIDQEHDDDDNNNNNMSMLYNPDNTSTTVPSSFLDALRGGSFLENAPNGFHHKMYYGNYGGMLGANSEEMGMNYDQEMSIGTTSASTIMTTTVKQEMCGIARSSEGDQNNNSKVLWGFPWQQMNEDHLNKMNMSDFESTNRQNWSAFGVSSNWHGLINSPLM

> CaDof29

MTSQDNKDDSQSSGGGPAGARPKEPALNCPRCDSPNTKFCYYNNYSLSQPRHFCKTCRRYWTKGGALRNVPIGGGCRKNKKIKTCSSSSRDSKDTTSGSSDIGGLKFFHGLSPAMDFQLGGLNFPRLNNTSTSTGGSIFNQFSTFGDISTTTTNCATNIGSSSCFNLDPLGSCSGSLLGFNNFPFSSSILKQGTTAVQEMGSMGVHHGTMASSIESLSSINQDLHWKLQQQRLAMLFGGENQKENNIVSSSIPFDDQNQNQNQNQNQIQIQKPQPILFQNLEISSSKQQEDHHHQEATTFGNDNINSRKDVNITIGSHGNNLATEWFFDDSFGVNPNSTNTINENEDQNGNNWNSTIQAWNNLNQYSTLP

> CaDof30

MVFSSFPVYLDHPNLHHLQQEPDHHQQGSPGLDNPQLPPVQPPTLVGGGAGSIRPGSMVDRARIARLPLPEPGLKCPRCDSSNTKFCYYNNYNLSQPRHFCKNCRRYWTKGGALRNVPVGGGCRRNKRNKSTSSSVSWGNGISCRE

> CaDof31

MQDPSSLIYSQIKPQFPDQEVLKCPRCDSINTKFCYYNNYNLSQPRHFCKNCKRYWTKGGILRNIPVGGSSRKNSKRSSSSSSSSKQSLRTSSLSPSSAQNGKIEVFPTPAVPAFDQESPILDANGPFGPFLVSNGSEIGNLLEGLNPNGPNYSGSDDAAAQSENNSTRDDEYLDVQKGEDSNCWNVNTNSWADLAIYTPGSRFQ

> CaDof32

MTLEASEKRVTKQQTAGGVLPPAQEPDQQLPCPRCDSTNTKFCYYNNYNLSQPRHFCKSCRRYWTHGGTLRDIPIGGGSRKNAKRCRIYTSTPFSSSTAVSPHGNSPFLVPLPTANQLLYGSDVKPCLNMSGNNFTSLLSSHGPTGVLALGGIEDVSFSIGRAAVWPFPGAPDSFIRNYDSGVGASMWQFSG

> CaDof33

MQDIHPIGGGGGRFYGGGGDRRLRPNNHQNHQALKCPRCGSLNTKFCYYNNYNLSQPRHFCKSCRRYWTKGGVLRNVPVGGGCRKSKRAKLKSSSINAVAEVAEAQEEQKSDTNSSSESSSLTATTTAATAAAAVAEVTATGNTSGGATTEDVSATSSNSASTYLNFPDSSNFFIPHSTTDNHHTFDDQPLTENFSSMMTSSNDPSMVGFNIAEIPAYRLPENQSSMIETLPSGDLKMEQTGTGYLNQTDRVEFPGLQQNRIDNSELASLDWQTGGGGDHGLYDLTGTVDQSYWNQTQWGENDNSLNFLP
