# Supplementary figures and images for: Genome-Wide Identification and Expression Profile of Dof Transcription Factor Gene Family in Pepper (Capsicum annuum L.)
Source: Front Plant Sci. 2016 Apr 29;7:574. doi: 10.3389/fpls.2016.00574 (PMC4850169; doi:10.3389/fpls.2016.00574)

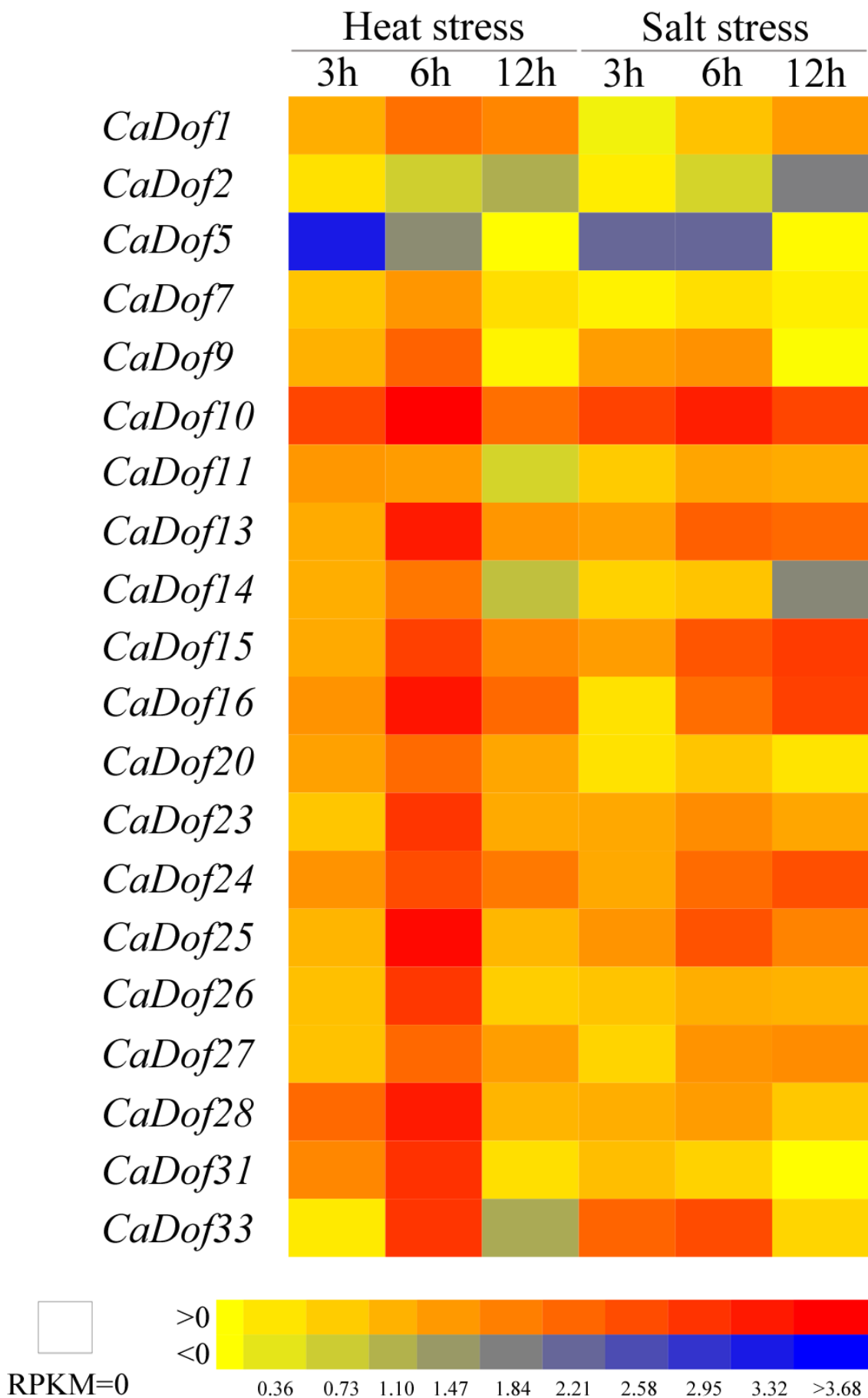

Supplement: Additional file 6 — The heat map representation of CaDof genes in response to heat and salt stress. [file Image1.PDF]
